# Halogenated and Nonhalogenated Metabolites from the Marine-Alga-Endophytic Fungus *Trichoderma asperellum* cf44-2

**DOI:** 10.3390/md16080266

**Published:** 2018-08-02

**Authors:** Yin-Ping Song, Feng-Ping Miao, Sheng-Tao Fang, Xiu-Li Yin, Nai-Yun Ji

**Affiliations:** 1 Yantai Institute of Coastal Zone Research, Chinese Academy of Sciences, Yantai 264003, China; ypsong@yic.ac.cn (Y.-P.S.); fpmiao@yic.ac.cn (F.-P.M.); stfang@yic.ac.cn (S.-T.F.); xlyin@yic.ac.cn (X.-L.Y.); 2 University of Chinese Academy of Sciences, Beijing 100049, China

**Keywords:** *Trichoderma*, terpene, trichodenone, diketopiperazine, oxazole

## Abstract

One new bisabolane sesquiterpene, bisabolan-1,10,11-triol (**1**), one new norbisabolane sesquiterpene, 12-nor-11-acetoxybisabolen-3,6,7-triol (**2**), two new naturally occurring monoterpenes, (7*S*)- and (7*R*)-1-hydroxy-3-*p*-menthen-9-oic acids (**3** and **4**), one new naturally occurring trichodenone, dechlorotrichodenone C (**5**), one new chlorine-containing trichodenone, 3-hydroxytrichodenone C (**6**), one new diketopiperazine, methylcordysinin A (**7**), and one new naturally occurring oxazole derivative, 4-oxazolepropanoic acid (**8**), were isolated from the culture of a marine brown alga-endophytic strain (cf44-2) of *Trichoderma asperellum*. Their structures and relative configurations were determined by extensive 1D/2D NMR and mass spectrometric data, and the absolute configurations of **3**–**6** were assigned by analysis of the ECD spectra aided by quantum chemical computations. Compounds **1**, **2**, **5**, and **6** showed growth inhibition of some marine phytoplankton species and pathogenic bacteria.

## 1. Introduction

**Figure 1:**
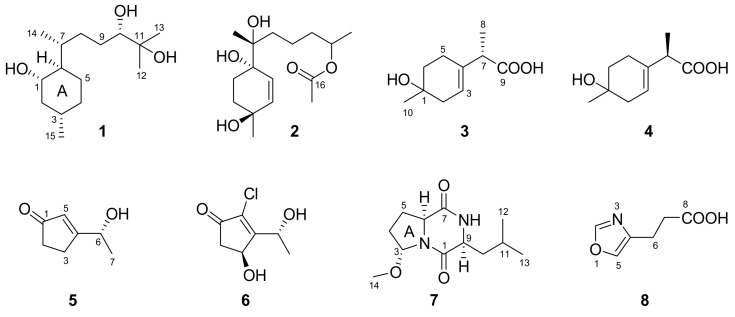
Structures of compounds **1**–**8**.

Marine organisms are known for the production of halogenated secondary metabolites, attracting much attention for natural product research during the last seven decades [1]. To date, a large number of halogenated compounds, such as monoterpenes, sesquiterpenes, diterpenes, triterpenes, acetogenins, and phenols, with various biological activities and ecological functions, have been discovered in marine red, brown, and green algae as well as cyanobacteria [2,3]. Although marine algicolous fungi (MAF), including endophytes and epiphytes, have also been proven to be a large reservoir of structurally unique and biologically active compounds, less halogenated structures were obtained from them [4]. However, *Trichoderma* strains associated with marine algae have an excellent ability to produce halogenated organic molecules [5,6], which greatly encouraged our further survey on them. As a result, one new bisabolane sesquiterpene (**1**), one new norbisabolane sesquiterpene (**2**), two new naturally occurring monoterpenes (**3** and **4**) [7], one new naturally occurring trichodenone (**5**) [8], one new chlorine-containing trichodenone (**6**), one new diketopiperazine (**7**), and one new naturally occurring oxazole derivative (**8**) [9], were isolated and identified from the culture of *T. asperellum* cf44-2, a marine brown alga-endophytic isolate. Herein, the details of isolation, structure elucidation, and bioactivity of these compounds (Figure 1) are described.

## 2. Results and Discussion

**Figure 2:**
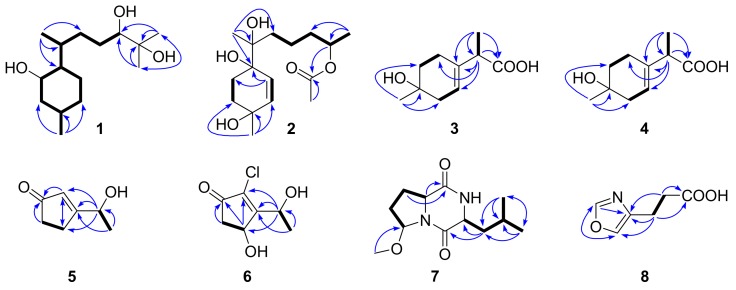
Key HMBC (arrows) and COSY (bold lines) correlations of **1**–**8**.

**Figure 3:**
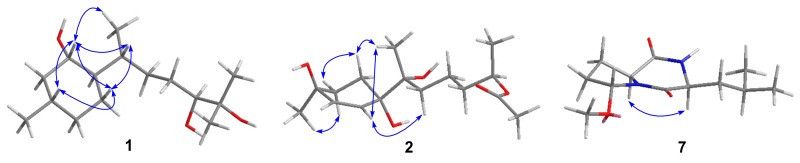
Key NOE correlations (arrows) of **1**, **2**, and **7**.

Compound **1** was isolated as a colorless oil. A molecular formula of C_15_H_30_O_3_ was determined by HREIMS (*m*/*z* 258.2191 [M]^+^), requiring one degree of unsaturation. The ^1^H NMR spectrum (Table 1) in conjunction with HSQC data showed notable signals including two methyl doublets, two methyl singlets, and one double doublet and one double triplet attributable to two oxymethines. The ^13^C NMR spectrum (Table 1) exhibited 15 resonances, classified into four methyls, five methylenes, five methines, and one nonprotonated carbon by DEPT experiments. HMBC correlations from Me-12 and Me-13 to C-10 and C-11 established the connectivity at C-11, which was elongated thoroughly to C-14 and C-15 by analysis of COSY correlations (Figure 2). HMBC correlations from Me-14 to C-6, C-7, and C-8 and from Me-15 to C-2, C-3, and C-4 further validated the planar structure of **1**, a bisabolane derivative. To satisfy the unsaturation requirement, C-1, C-10, and C-11 resonating at *δ*_C_ 70−80 should be hydroxylated. Thus, **1** was deduced to be bisabolan-1,10,11-triol, and its relative configuration was assigned by analysis of coupling constants and NOE correlations (Figure 3) and by comparison of NMR data with those of similar structures. In view of the large coupling constants, H-1 was proposed to be axial and opposite to H-6, whereas H-1 was *syn* to H-3 by their NOE correlation. The relative configurations around ring A was also supported by the identical NMR data with those of an analogue [10]. The relative configurations of exocyclic chiral centers were difficult to be determined. However, the rotation of a single bond between two asymmetric carbons was restricted to some extent, which enabled the assignment of relative configurations by coupling constants or NOE correlations [11,12]. NOE correlations of Me-14 with H-1 and of H-7 with H-1 and H-5b suggested the R* configuration of C-7, and the S* configuration of C-10 was speculated by comparison of NMR data with literature [13,14]. Based on the splitting pattern and coupling constants of H-10, the C-9−C-10 bond rotation was also staggered.

Compound **2** was purified as a colorless oil, and its molecular formula was assigned to be C_16_H_28_O_5_ by HREIMS (*m*/*z* 300.1957 [M]^+^), implying three degrees of unsaturation. The ^1^H NMR spectrum (Table 1) alongside HSQC data displayed one methyl doublet, three methyl singlets, one multiplet ascribable to an oxymethine, and two double doublets attributable to two olefinic protons amongst others. The ^13^C NMR and DEPT spectra (Table 1) demonstrated the presence of four methyls, five methylenes, three methines, and four quaternary carbons. The connectivity at C-3 was established by HMBC correlations from H-15 to C-2, C-3, and C-4 (Figure 2), which was extended to C-1 and C-5, respectively, by COSY correlations of H-1/H-2 and H-4/H-5 and then formed ring A via C-6 by HMBC correlations from H-5 to C-1 and C-6. On the other hand, COSY correlations of H-8/H-9/H-10/H-11/H-13 indicated the linkage of the side chain moiety, which was attached to C-6 through C-7 by HMBC correlations from H-14 to C-6, C-7, and C-8. Additionally, an acetoxy group was bonded to C-11 by HMBC correlations from H-11 and H-17 to C-16. Thus, the structure of **2** was assigned to be 12-nor-11-acetoxybisabolen-3,6,7-triol. The side chain unit was undoubtedly oriented to be equatorial, while the large coupling constants of H-1a and H-2a suggested them to be axial. Additionally, Me-15 was *syn* to H-2a by their NOE correlation, and C-7 was tentatively assigned as the R* configuration by NOE correlations of Me-14 with H-1a and H-5 and of H-5 with H-8 (Figure 3). However, the relative configuration at C-11 remained unresolved.

Compound **3** was obtained as a colorless oil with a molecular formula of C_10_H_16_O_3_ given by interpretation of HREIMS (*m*/*z* 184.1100), suggesting three degrees of unsaturation. The EI mass spectrum showed fragment ion peaks at *m*/*z* 166 and 138, indicating the presence of hydroxy and carboxyl groups. In combination with HSQC data, the ^1^H NMR spectrum (Table 2) exhibited one methyl singlet, one methyl doublet, one methine quartet, and one broad singlet for an olefinic proton as well as six signals at *δ*_H_ 1.5–2.4 due to three methylenes. The ^13^C NMR and DEPT spectra (Table 3) displayed 10 resonances, corresponding to two methyls, three methylenes, two methines, and three quaternary carbons. The four deshielded ^13^C NMR signals between *δ*_C_ 68 and 180 indicated the presence of an oxygenated quaternary carbon, a trisubstituted vinyl group, and a carboxyl group, which were linked together via C-2 and C-7, respectively, and then methylated based on the COSY correlation between H-2 and H-3 and HMBC correlations from H-10 to C-1 and C-2 and from H-8 to C-4, C-7, and C-9 (Figure 2). The remaining two methylene groups were fitted together by their COSY correlations and located between C-1 and C-4 by HMBC correlations from H-7 to C-5 and from H-10 to C-6. Thus, **3** was assigned to be 1-hydroxy-3-*p*-menthen-9-oic acid.

Compound **4** was acquired as a colorless oil and assigned a molecular formula of C_10_H_16_O_3_, the same as for **3**, by HREIMS (*m*/*z* 184.1097). Its ^1^H and ^13^C NMR as well as EIMS data closely resembled those of **3**, and COSY and HMBC correlations (Figure 2) also suggested **4** to be 1-hydroxy-3-*p*-menthen-9-oic acid. Thus, **4** should be an epimer of **3** in view of only two chiral centers in each of them, and the epimerization probably happened at C-7 due to the enolizable α proton next to the carboxyl group. 1-Hydroxy-3-*p*-menthen-9-oic acid was previously obtained by transformation of 3-*p*-menthene using a soil pseudomonad, but its stereochemistry and NMR data were not determined [7].

Compounds **5** and **6** were isolated as colorless oils. The former was identified to be dechlorotrichodenone C, previously obtained as an intermediate in the synthesis of trichodenone C, by the identical NMR data [8,15]. The molecular formula of **6** was assigned to be C_7_H_9_ClO_3_ based on HREIMS (*m/z* 176.0236), one more oxygen atom than trichodenone C [15]. Its ^1^H and ^13^C NMR spectroscopic data (Table 2 and Table 3) appeared similar to those of trichodenone C, except for the presence of signals for a hydroxymethine group and the lack of signals for a methylene group. The hydroxymethine group was situated at C-3 by its HMBC correlation with H-6, and other HMBC and COSY correlations (Figure 2) further confirmed the planar structure of **6**, trivially named 3-hydroxytrichodenone C.

Compound **7** was purified as a white powder. A molecular formula of C_12_H_20_N_2_O_3_ was given by interpretation of HREIMS (*m/z* 240.1488), requiring four degrees of unsaturation. A fragment ion peak at *m*/*z* 209 in the EI mass spectrum corresponded to the presence of a methoxy group. The ^1^H NMR spectrum (Table 2) notably exhibited two methyl doublets, one methoxy singlet, and one double doublet, one doublet, and one triplet assignable to three oxygenated/nitrogenated methines. Aided by DEPT experiments, 12 resonances in the ^13^C NMR spectrum (Table 3) were sorted into three methyls, three methylenes, four methines, and two quaternary carbons. A detailed comparison of NMR data with those of cordysinin A revealed that a methoxy group, rather than a hydroxy group, was present in **7** [16], and its position was supported by its HMBC correlation with C-3. Other HMBC and COSY correlations (Figure 2) further corroborated the gross structure of **7**, trivially named methylcordysinin A. The NOE correlation between H-6 and H-9 (Figure 3) placed them on the same face of the molecule, whereas they were opposite to H-3 by comparison of splitting patterns and coupling constants with those of (2*S*,4*R*)-1-acetyl-4-hydroxyproline ethyl ester and its methoxylated derivatives [17].

Compound **8** was acquired as a colorless oil. Its molecular formula was established as C_6_H_7_NO_3_ on the basis of HREIMS (*m/z* 141.0428), and the base peak at *m*/*z* 96 in the EI mass spectrum suggested the presence of a carboxyl group. The ^1^H NMR spectrum (Table 2) displayed two triplets for two methylenes and two singlets for two aromatic protons, agreeing with those of 4-oxazolepropanoic acid [9]. On the other hand, the COSY correlation between H-6 and H-7 and HMBC correlations from H-6 and H-7 to C-8 (Figure 2) suggested the presence of a propionic acid side chain, and the residual ^13^C NMR data indicated the presence of an oxazole ring [18]. The connectivity of these two moieties was confirmed by HMBC correlations from H-6 to C-4 and C-5 and from H-7 to C-4. Thus, **8** was deduced to be 4-oxazolepropanoic acid, which was previously synthesized from 4-oxazoleacrylic acid by diimide and palladium charcoal reduction methods [9].

**Figure 4:**
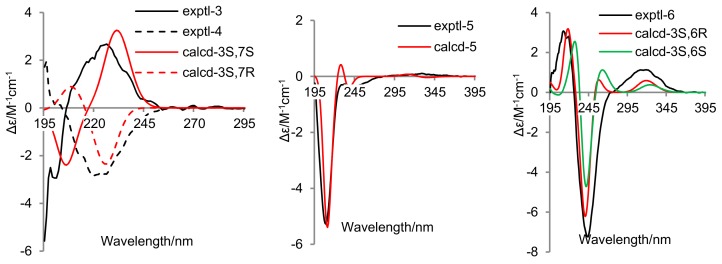
Experimental and calculated ECD spectra of **3**–**6**.

In order to establish the absolute configurations of **3**–**6**, their electronic circular dichroism (ECD) spectra were determined in MeOH and computed at the B3LYP/6–31G(d) level in MeOH with the integral equation formalism variant (IEF) of the polarizable continuum model (PCM) after conformational optimization at the same level through Gaussian 09 software [19]. Based on the comparison of experimental and calculated ECD spectra (Figure 4) depicted by SpecDis software (Version 1.51) with sigma =0.2 [20], the absolute configurations at C-7 of **3** and **4** were assigned as *S* and *R*, respectively. These two compounds should possess the same absolute configuration at C-1 due to their epimeric relationship, but it remained unable to be resolved. As indicated in literature [8], the specific optical rotation value of **5** was too low to be determined. However, the ECD spectrum of **5** showed a negative Cotton effect at 209 nm (Figure 4), and its accordance with the calculated one confirmed the absolute configuration at C-6 to be *R*. The experimental ECD curve of **6** agreed with the calculated ones (Figure 4) of both 3*S*/6*R* and 3*S*/6*S* isomers, which suggested the 3*S* configuration of **6**. In view of the biogenic consideration, the absolute configuration at C-6 was assigned as the same as that of **5**. In addition, the absolute configuration of **7** was deduced to be the same as that of cordysinin A by their similar specific optical rotation data [16]. Unfortunately, the absolute configurations of **1** and **2** remained unresolved, due to the failure in crystallizations and Mosher’s reactions.

Compounds **1**, **2**, **5**, and **6** were evaluated for the inhibition of four marine phytoplankton species (*Chattonella marina*, *Heterosigma akashiwo*, *Karlodinium veneficum*, and *Prorocentrum donghaiense*) and four marine-derived pathogenic bacteria (*Vibrio parahaemolyticus*, *V. anguillarum*, *V. harveyi*, and *V. splendidus*) [21,22], and the results were shown in Table 4. All of them exhibited growth inhibition of the four phytoplankton species tested, and **5** with IC_50_ values ranging from 4.2–8.5 μg/mL was more active than the others. Additionally, **1**, **2**, **5**, and **6** showed weak antibacterial activities against the four *Vibrio* bacteria tested, with the inhibitory zone diameters of 6.2–8.5 mm at 20 μg/disk. Among them, **6** is the most antibacterial compound.

## 3. Materials and Methods

### 3.1. General Experimental Procedures

NMR spectra were measured on a Bruker Avance III 500 NMR spectrometer (Bruker Corp., Billerica, MA, USA). Low and high resolution EI mass spectra were determined on an Autospec Premier P776 mass spectrometer at 70 eV (Waters Corp., Milford, MA, USA). ECD spectra were acquired on a Chirascan CD spectrometer (Applied Photophysics Ltd., Surrey, UK). IR spectra were recorded on a JASCO FT/IR-4100 spectrometer (JASCO, Tokyo, Japan). Optical rotations were obtained on an SGW-3 polarimeter with a 2 mL (length 10 cm) cell (Shanghai Shenguang Instrument Co., Ltd., Shanghai, China). HPLC separation was operated on an Agilent HPLC system (1260 infinity quaternary pump, 1260 infinity diode-array detector) using an Eclipse SB-C18 (5 μm, 9.4 × 250 mm) column (Agilent Technologies Inc., Santa Clara, CA, USA). Column chromatography (CC) was carried out with silica gel (200–300 mesh, Qingdao Haiyang Chemical Co., Qingdao, China), RP-18 (AAG12S50, YMC Co., Ltd., Kyoto, Japan), and Sephadex LH-20 (GE Healthcare, Uppsala, Sweden). Thin-layer chromatography (TLC) was performed with precoated silica gel plates (GF-254, Qingdao Haiyang Chemical Co., Qingdao, China).

### 3.2. Fungal Material and Fermentation

*Trichoderma asperellum* cf44-2 was obtained from the inner tissue of the marine brown alga *Sargassum* sp. collected from Zhoushan Islands in August 2010. It was identified by morphological observation and by analysis of the ITS regions of its rDNA (GenBank accession no. MG696741). The fermentation was performed statically at room temperature for 30 days in 200 × 1 L Erlenmeyer flasks, each filled with 300 mL of media comprising 500 mL potato (200 g) broth, 20 g glucose, 5 g peptone, 5 g yeast extract powder, and 500 mL natural seawater from the coast of Yantai.

### 3.3. Extraction and Isolation

The whole fermented cultures were filtered to separate mycelia from broth. The former were exhaustively extracted with CH_2_Cl_2_ and MeOH (1:1, *v*/*v*) through maceration at room terperature, and the latter was directly extracted with EtOAc and concentrated by evaporation under vacuum to afford an extract (31.3 g). Furthermore, the extract of mycelia was partitioned between EtOAc and H_2_O to give an EtOAc-soluble gum (52.4 g). Based on the similar TLC profiles, these two parts were combined and then subjected to silica gel CC with step-gradient solvent systems consisting of petroleum ether (PE)/EtOAc (50:1, 20:1, 10:1, 5:1, 2:1, 1:1, and 0:1) and CH_2_Cl_2_/MeOH (20:1, 10:1, 5:1, and 0:1) to yield 12 fractions (Frs. 1-12). Fr. 6 eluted with PE/EtOAc (1:1) and was further purified by CC on RP-18 (MeOH/H_2_O, 1:4 to 11:9) and Sephadex LH-20 (MeOH) and preparative TLC (CH_2_Cl_2_/MeOH, 15:1) to give **1** (2.7 mg) and a subfraction that was then purified by semipreparative HPLC (MeOH/H_2_O, 2:5 to 9:20) to produce **3** (2.1 mg) and **4** (2.3 mg). Fr. 8 eluted with PE/EtOAc (1:1) and was further purified by CC on RP-18 (MeOH/H_2_O, 1:4) and Sephadex LH-20 (MeOH) and preparative TLC (CH_2_Cl_2_/MeOH, 10:1) to obtained **5** (4.0 mg), **6** (1.1 mg), and **8** (1.5 mg). Fr. 9 eluted with EtOAc and was further purified by RP-18 CC (MeOH/H_2_O, 1:4 to 3:7) and preparative TLC (CH_2_Cl_2_/MeOH, 12:1) to afford **2** (2.1 mg) and **7** (2.0 mg).

Isabolan-1,10,11-triol (**1**): Colorless oil; ${\left[\alpha \right]}_{D}^{25}$ −11 (*c* 0.14, MeOH); IR (KBr) *v*_max_ 3390, 2924, 2866, 1454, 1377, 1161, 1080, 1041 cm^−1^; ^1^H and ^13^C NMR data, Table 1; EIMS *m/z* (%) 258 [M]^+^ (7), 223 (100), 205 (30), 163 (41), 109 (49), 95 (60), 81 (73), 69 (61), 59 (58), 55 (71); HREIMS *m/z* 258.2191 [M]^+^ (calcd for C_15_H_30_O_3_, 258.2195).

12-nor-11-Acetoxybisabolen-3,6,7-triol (**2**): Colorless oil; ${\left[\alpha \right]}_{D}^{25}$ −8.3 (*c* 0.08, MeOH); IR (KBr) *v*_max_ 3402, 2962, 2931, 1712, 1647, 1381, 1261, 1103, 1030 cm^−1^; ^1^H and ^13^C NMR data, Table 1; EIMS *m/z* (%) 300 [M]^+^ (<1), 153 (20), 128 (16), 113 (58), 110 (100), 109 (21), 95 (32), 86 (27), 71 (17); HREIMS *m/z* 300.1957 [M]^+^ (calcd for C_16_H_28_O_5_, 300.1937).

(7*S*)-1-Hydroxy-3-p-menthen-9-oic acid (**3**): Colorless oil; ${\left[\alpha \right]}_{D}^{25}$ −70 (*c* 0.016, MeOH); ^1^H and ^13^C NMR data, Table 2 and Table 3; EIMS *m/z* (%) 184 [M]^+^ (13); 166 (80), 138 (60), 123 (62), 95 (59), 81 (60), 58 (70), 44 (100); HREIMS *m/z* 184.1100 [M]^+^ (calcd for C_10_H_16_O_3_, 184.1099).

(7*R*)-1-Hydroxy-3-p-menthen-9-oic acid (**4**): Colorless oil; ${\left[\alpha \right]}_{D}^{25}$ −99 (*c* 0.040, MeOH); ^1^H and ^13^C NMR data, Table 2 and Table 3; EIMS *m/z* (%) 184 [M]^+^ (13); 167 (42), 166 (100), 138 (71), 123 (73), 121 (65), 95 (71), 81 (74), 58 (78), 44 (100); HREIMS *m/z* 184.1097 [M]^+^ (calcd for C_10_H_16_O_3_, 184.1099).

Dechlorotrichodenone C (**5**): Colorless oil; IR (KBr) *v*_max_ 3410, 2978, 2927, 1697, 1612, 1435, 1265, 1153, 1080, 864 cm^−1^; ^1^H and ^13^C NMR data, Table 2 and Table 3.

3-Hydroxytrichodenone C (**6**): Colorless oil; ${\left[\alpha \right]}_{D}^{25}$ +7.5 (*c* 0.040, MeOH); IR (KBr) *v*_max_ 3398, 2924, 2854, 1720, 1628, 1388, 1072 cm^−1^; ^1^H and ^13^C NMR data, Table 2 and Table 3; EIMS *m/z* (%) 176 [M]^+^ (4), 177 (58), 175 (55), 132 (31), 121 (100), 57 (40); HREIMS *m/z* 176.0236 [M]^+^ (calcd for C_7_H_9_^35^ClO_3_, 176.0240).

Methylcordysinin A (**7**): White powder; ${\left[\alpha \right]}_{D}^{25}$ −81 (*c* 0.050, MeOH); IR (KBr) *v*_max_ 3278, 2947, 2873, 1655, 1412, 1095 cm^−1^; ^1^H and ^13^C NMR data, Table 2 and Table 3; EIMS *m/z* (%) 240 [M]^+^ (6), 209 (40), 184 (55), 152 (100), 100 (41), 86 (65), 68 (45); HREIMS *m/z* 240.1488 [M]^+^ (calcd for C_12_H_20_N_2_O_3_, 240.1474).

4-Oxazolepropanoic acid (**8**): Colorless oil; ^1^H and ^13^C NMR data, Table 2 and Table 3; EIMS *m/z* (%) 141 [M]^+^ (28), 123 (20), 97 (28), 96 (100), 95 (80), 54 (38), 42 (43), 40 (65); HREIMS *m/z* 141.0428 [M]^+^ (calcd for C_6_H_7_NO_3_, 141.0426).

Detailed NMR data can be found in Supplementary Figures S1–S51.

## 4. Conclusions

A chemical survey on a marine-alga-endophytic isolate (cf44-2) of the fungus *Trichoderma asperellum* has resulted in the isolation and identification of four new compounds (**1**, **2**, **6**, and **7**) and four new naturally occurring ones (**3**–**5** and **8**), involving two sesquiterpenes (**1** and **2**), two monoterpenes (**3** and **4**), two trichodenones (**5** and **6**), and two nitrogen-bearing metabolites (**7** and **8**). It is worth mentioning that 3-hydroxytrichodenone C (**6**) is a new chlorine-containing molecule, and it along with **1**, **2**, and **5** can inhibit the four marine phytoplankton species and four marine-derived *Vibrio* bacteria tested.

## Figures and Tables

**Table 1:** ^1^H and ^13^C NMR data for **1** and **2** (*δ* in ppm, *J* in Hz).

Pos.	1 (in CDCl_3_)		2 (in CD_3_OD)	
	*δ*_H_ (*J* in Hz)	*δ*_C_, Type	*δ*_H_ (*J* in Hz)	*δ*_C_, Type
1a	3.44, td (10.5, 4.3)	71.5, CH	1.97, td (13.4, 2.9)	27.8, CH_2_
1b			1.57, m	
2a	1.97, dddd (12.1, 4.2, 3.6, 2.0)	45.3, CH_2_	1.88, td (13.6, 3.0)	34.3, CH_2_
2b	0.97, ddd (12.1, 12.1, 10.7)		1.68, m	
3	1.42, m	31.8, CH		67.3, C
4a	1.66, dm (12.9)	34.7, CH_2_	5.73, dd (10.2, 1.6)	136.8, CH
4b	0.84, m			
5a	1.59, dq (13.2, 3.3)	23.5, CH_2_	5.90, dd (10.2, 1.6)	131.3, CH
5b	1.02, qd (13.1, 3.5)			
6	1.20, m	49.0, CH		74.9, C
7	2.03, m	30.8, CH		77.1, C
8a	1.53, m	32.7, CH_2_	1.73, m	36.3, CH_2_
8b	1.31, m		1.40, m	
9a	1.46, m	29.8, CH_2_	1.51, m	20.5, CH_2_
9b	1.36, m		1.41, m	
10a	3.36, dd (9.9, 2.0)	78.9, CH	1.60, m	37.7, CH_2_
10b			1.52, m	
11		73.3, C	4.89, m	72.6, CH
12	1.16, s	23.3, CH_3_		
13	1.21, s	26.7, CH_3_	1.22, d (6.3)	20.2, CH_3_
14	0.81, d (6.9)	14.0, CH_3_	1.10, s	21.2, CH_3_
15	0.91, d (6.6)	22.3, CH_3_	1.27, s	30.3, CH_3_
16				172.8, C
17			2.01, s	21.3, CH_3_

**Table 2:** ^1^H NMR data for **3**–**8** (*δ* in ppm, *J* in Hz).

Pos.	3 (in CDCl_3_)	4 (in CDCl_3_)	5 (in CDCl_3_)	6 (in CD_3_OD)	7 (in CD_3_OD)	8 (in CD_3_OD)
2a	2.19, d (18.0)	2.16, brs	2.45, m	2.88, ddd (18.6, 6.3, 0.9)		8.11, s
2b	2.12, d (17.6)			2.30, dd (18.6, 1.7)		
3	5.54, brs	5.54, brs	2.64, m	5.14, dd (6.3, 1.5)	5.24, d (5.0)	
4a					2.05, dt (13.2, 3.3)	
4b					1.91, m	
5a	2.28, m	2.18, m	6.13, q (1.7)		2.15, m	7.69, s
5b	2.04, brd (17.4)	2.12, m			2.15, m	
6a	1.72, dt (13.0, 5.0)	1.73, dt (13.1, 5.2)	4.66, q (6.7)	4.97, q (6.9)	4.26, t (8.8)	2.83, t (7.4)
6b	1.60, ddd (12.9, 8.5, 6.4)	1.60, dt (13.1, 6.7)				
7	3.13, q (6.8)	3.13, q (6.9)	1.45, d (6.7)	1.51, d (6.9)		2.64, t (7.4)
8	1.29, d (7.1)	1.29, d (7.1)				
9					4.09, dd (8.1, 4.0)	
10a	1.26, s	1.25, s			1.93, m	
10b					1.52, m	
11					1.89, m	
12					0.99, d (6.4)	
13					0.97, d (6.3)	
14					3.37, s	

**Table 3:** ^13^C NMR data for **3**–**8** (*δ* in ppm).

Pos.	3 (in CDCl_3_)	4 (in CDCl_3_)	5 (in CDCl_3_)	6 (in CD_3_OD)	7 (in CD_3_OD)	8 (in CD_3_OD)
1	68.6, C	68.7, C	209.7, C	200.1, C	171.1, C	
2	39.8, CH_2_	39.8, CH_2_	35.4, CH_2_	44.0, CH_2_		153.4, CH
3	121.8, CH	122.0, CH	27.9, CH_2_	68.4, CH	88.5, CH	
4	135.6, C	135.6, C	184.4, C	175.4, C	31.8, CH_2_	140.1, C
5	24.3, CH_2_	24.1, CH_2_	128.1, CH	131.5, C	25.3, CH_2_	136.5, CH
6	35.4, CH_2_	35.4, CH_2_	68.0, CH	66.3, CH	60.5, CH	22.4, CH_2_
7	46.3, CH	46.3, CH	22.2, CH_3_	21.8, CH_3_	173.6, C	33.7, CH_2_
8	15.6, CH_3_	15.7, CH_3_				176.2, C
9	179.5, C	179.7, C			55.0, CH	
10	28.6, CH_3_	28.4, CH_3_			38.8, CH_2_	
11					25.9, CH	
12					23.4, CH_3_	
13					22.0, CH_3_	
14					57.4, CH_3_	

**Table 4:** Antimicroalgal and antibacterial activities of **1**, **2**, **5**, and **6**.

	IC_50_ (μg/mL)				Inhibitory Zone Diameter (mm) at 20 μg/disk			
	*Chattonella marina*	*Heterosigma akashiwo*	*Karlodinium veneficum*	*Prorocentrum donghaiense*	*Vibrio parahaemolyticus*	*Vibrio anguillarum*	*Vibrio harveyi*	*Vibrio Splendidus*
**1**	– ^a^	30	27	– ^b^	6.7	6.5	6.5	6.3
**2**	15	8.4	10	14	6.4	7.5	7.0	6.3
**5**	4.2	7.2	8.5	6.9	6.2	7.0	6.5	6.2
**6**	30	35	39	37	6.7	8.5	8.0	6.5
K_2_Cr_2_O_7_	0.46	0.98	0.89	1.92				
chloramphenicol					20	18	18	19

^a^ Inhibition rate was 65% at 100 μg/mL. ^b^ Inhibition rate was 50% at 100 μg/mL.

## Supplementary Materials

The following are available online at http://www.mdpi.com/1660-3397/16/8/266/s1, Figures S1–S32 and S38−S59: 1D/2D NMR and mass spectra of compounds **1**–**4** and **6**–**8**, Figures S33−S37: 1D/2D NMR spectra of compound **5**, Figures S60–S64: Energy-minimized conformers with populations of (3*S*,7*S*)- and (3*S*,7*R*)-1-hydroxy-3-*p*-menthen-9-oic acids, compound **5**, and (3*S*,6*R*)- and (3*S*,6*S*)-3-hydroxytrichodenone C.
