# Supplementary material for: Halogenated and Nonhalogenated Metabolites from the Marine-Alga-Endophytic Fungus Trichoderma asperellum cf44-2
Source: Mar Drugs. 2018 Aug 2;16(8):266. doi: 10.3390/md16080266 (PMC6117674; doi:10.3390/md16080266)
Supplement: Supplementary file 1 [file marinedrugs-16-00266-s001.pdf]

Supporting Information

Halogenated and Nonhalogenated Metabolites from the Marine-Alga-Endophytic Fungus *Trichoderma asperellum* cf44-2

Yin-Ping Song [1,2], Feng-Ping Miao [1], Sheng-Tao Fang [1], Xiu-Li Yin [1] and Nai-Yun Ji [1,*]

[1] Yantai Institute of Coastal Zone Research, Chinese Academy of Sciences, Yantai 264003, China; ypsong@yic.ac.cn (Y.-P.S.); fpmiao@yic.ac.cn (F.-P.M.); stfang@yic.ac.cn (S.-T.F.); xlyin@yic.ac.cn (X.-L.Y.)

[2] University of Chinese Academy of Sciences, Beijing 100049, China

* Correspondence: nyji@yic.ac.cn; Tel.: +86-535-210-9176

List of Supporting Information

Figure S1. ^{1}H NMR spectrum of compound **1** in $CDCl_3$.

Figure S2. ^{13}C NMR and DEPT spectra of compound **1** in $CDCl_3$.

Figure S3. HSQC spectrum of compound **1** in $CDCl_3$.

Figure S4. HMBC spectrum of compound **1** in $CDCl_3$.

Figure S5. COSY spectrum of compound **1** in $CDCl_3$.

Figure S6. NOESY spectrum of compound **1** in $CDCl_3$.

Figure S7. EIMS spectrum of compound **1**.

Figure S8. HREIMS spectrum of compound **1**.

Figure S9. ^{1}H NMR spectrum of compound **2** in CD_3OD.

Figure S10. ^{13}C NMR and DEPT spectra of compound **2** in CD_3OD.

Figure S11. HSQC spectrum of compound **2** in CD_3OD.

Figure S12. HMBC spectrum of compound **2** in CD_3OD.

Figure S13. COSY spectrum of compound **2** in CD_3OD.

Figure S14. NOESY spectrum of compound **2** in CD_3OD.

Figure S15. EIMS spectrum of compound **2**.

Figure S16. HREIMS spectrum of compound **2**.

Figure S17. ^{1}H NMR spectrum of compound **3** in $CDCl_3$.

Figure S18. ^{13}C NMR and DEPT spectra of compound **3** in $CDCl_3$.

Figure S19. HSQC spectrum of compound **3** in $CDCl_3$.

Figure S20. HMBC spectrum of compound **3** in $CDCl_3$.

Figure S21. COSY spectrum of compound **3** in $CDCl_3$.

Figure S22. NOESY spectrum of compound **3** in $CDCl_3$.

Figure S23. EIMS spectrum of compound **3**.

Figure S24. HREIMS spectrum of compound **3**.

Figure S25. ^{1}H NMR spectrum of compound **4** in $CDCl_3$.

Figure S26. ^{13}C NMR and DEPT spectra of compound **4** in $CDCl_3$.

Figure S27. HSQC spectrum of compound **4** in $CDCl_3$.

Figure S28. HMBC spectrum of compound **4** in $CDCl_3$.

Figure S29. COSY spectrum of compound **4** in $CDCl_3$.

Figure S30. NOESY spectrum of compound **4** in $CDCl_3$.

Figure S31. EIMS spectrum of compound **4**.

Figure S32. HREIMS spectrum of compound **4**.

Figure S33. ^{1}H NMR spectrum of compound **5** in $CDCl_3$.

Figure S34. ^{13}C NMR and DEPT spectra of compound **5** in $CDCl_3$.

Figure S35. HSQC spectrum of compound **5** in $CDCl_3$.
Figure S36. HMBC spectrum of compound **5** in $CDCl_3$.
Figure S37. COSY spectrum of compound **5** in $CDCl_3$.
Figure S38. ^{1}H NMR spectrum of compound **6** in CD_3OD.
Figure S39. ^{13}C NMR and DEPT spectra of compound **6** in CD_3OD.
Figure S40. HSQC spectrum of compound **6** in CD_3OD.
Figure S41. HMBC spectrum of compound **6** in CD_3OD.
Figure S42. COSY spectrum of compound **6** in CD_3OD.
Figure S43. EIMS spectrum of compound **6**.
Figure S44. HREIMS spectrum of compound **6**.
Figure S45. ^{1}H NMR spectrum of compound **7** in CD_3OD.
Figure S46. ^{13}C NMR and DEPT spectra of compound **7** in CD_3OD.
Figure S47. HSQC spectrum of compound **7** in CD_3OD.
Figure S48. HMBC spectrum of compound **7** in CD_3OD.
Figure S49. COSY spectrum of compound **7** in CD_3OD.
Figure S50. NOESY spectrum of compound **7** in CD_3OD.
Figure S51. EIMS spectrum of compound **7**.
Figure S52. HREIMS spectrum of compound **7**.
Figure S53. ^{1}H NMR spectrum of compound **8** in CD_3OD.
Figure S54. ^{13}C NMR and DEPT spectra of compound **8** in CD_3OD.
Figure S55. HSQC spectrum of compound **8** in CD_3OD.
Figure S56. HMBC spectrum of compound **8** in CD_3OD.
Figure S57. COSY spectrum of compound **8** in CD_3OD.
Figure S58. EIMS spectrum of compound **8**.
Figure S59. HREIMS spectrum of compound **8**.
Figure S60. Energy-minimized conformers with populations of (3*S*,7*S*)-1-hydroxy-3-*p*-menthen-9-oic acid.
Figure S61. Energy-minimized conformers with populations of (3*S*,7*R*)-1-hydroxy-3-*p*-menthen-9-oic acid.
Figure S62. Energy-minimized conformers with populations of compound **5**.
Figure S63. Energy-minimized conformers with populations of (3*S*,6*R*)-3-hydroxytrichodenone C.
Figure S64. Energy-minimized conformers with populations of (3*S*,6*S*)-3-hydroxytrichodenone C.

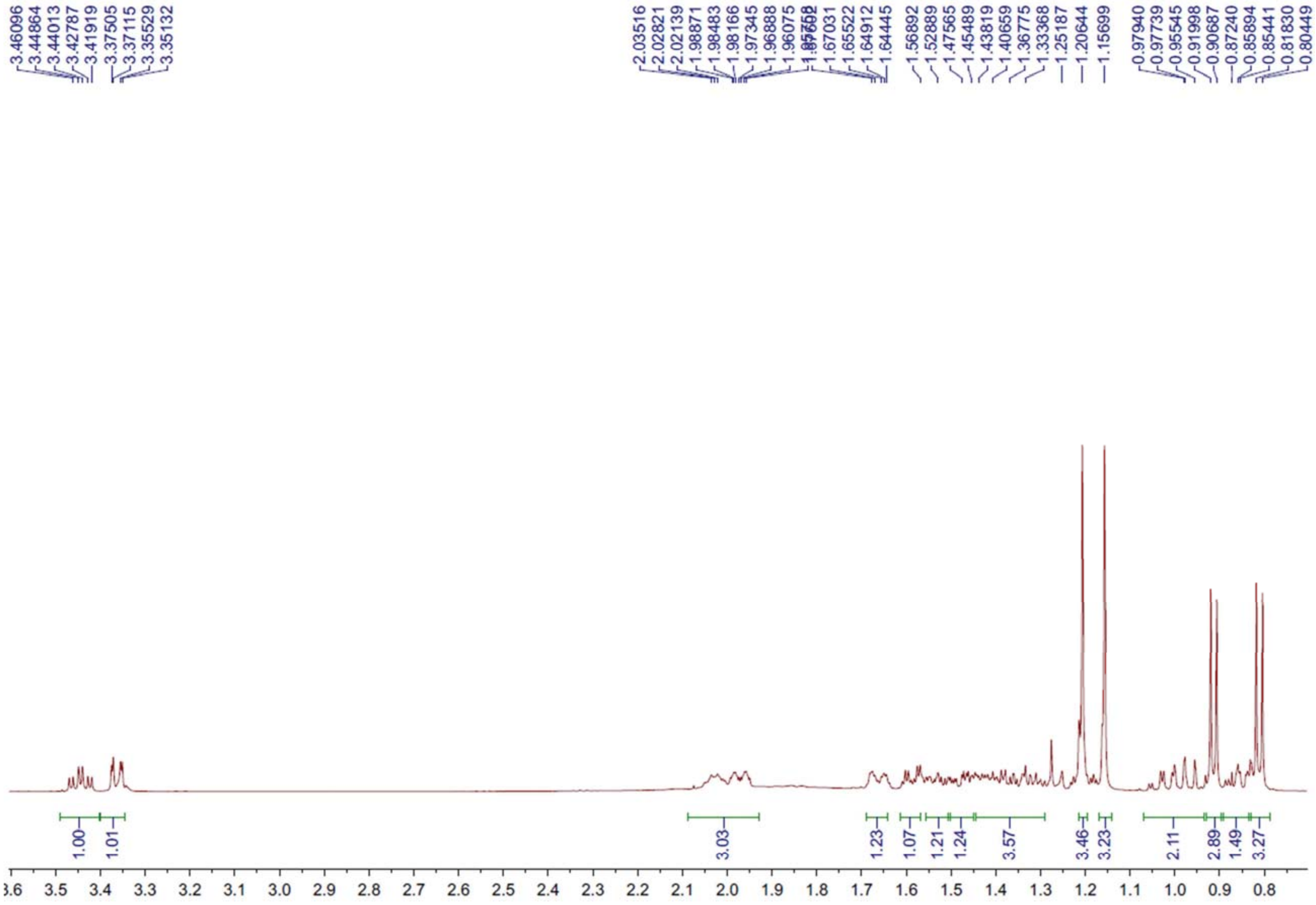

Figure S1. ^{1}H NMR spectrum of compound **1** in $CDCl_3$.

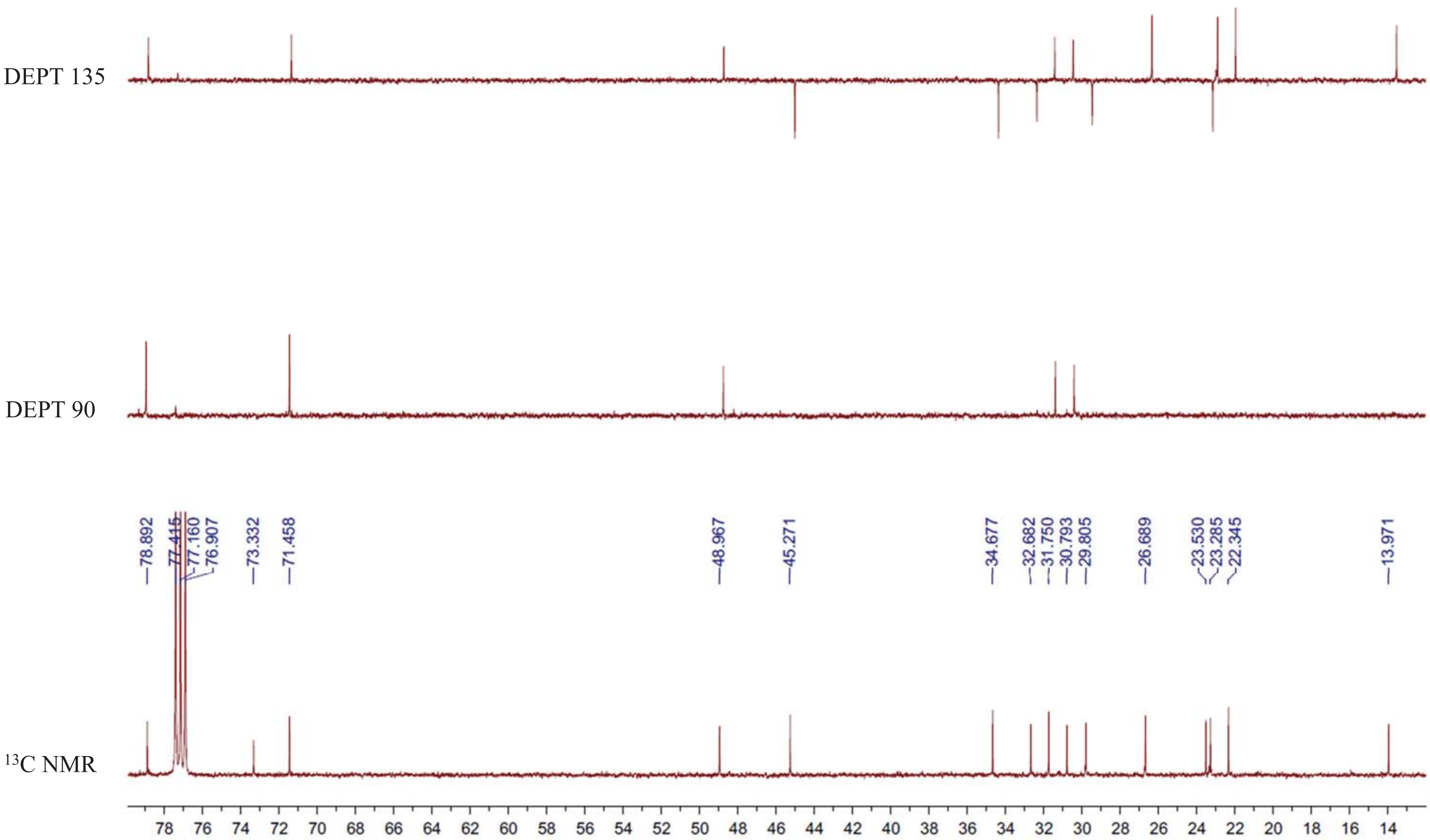

Figure S2. ^{13}C NMR and DEPT spectra of compound **1** in $CDCl_3$.

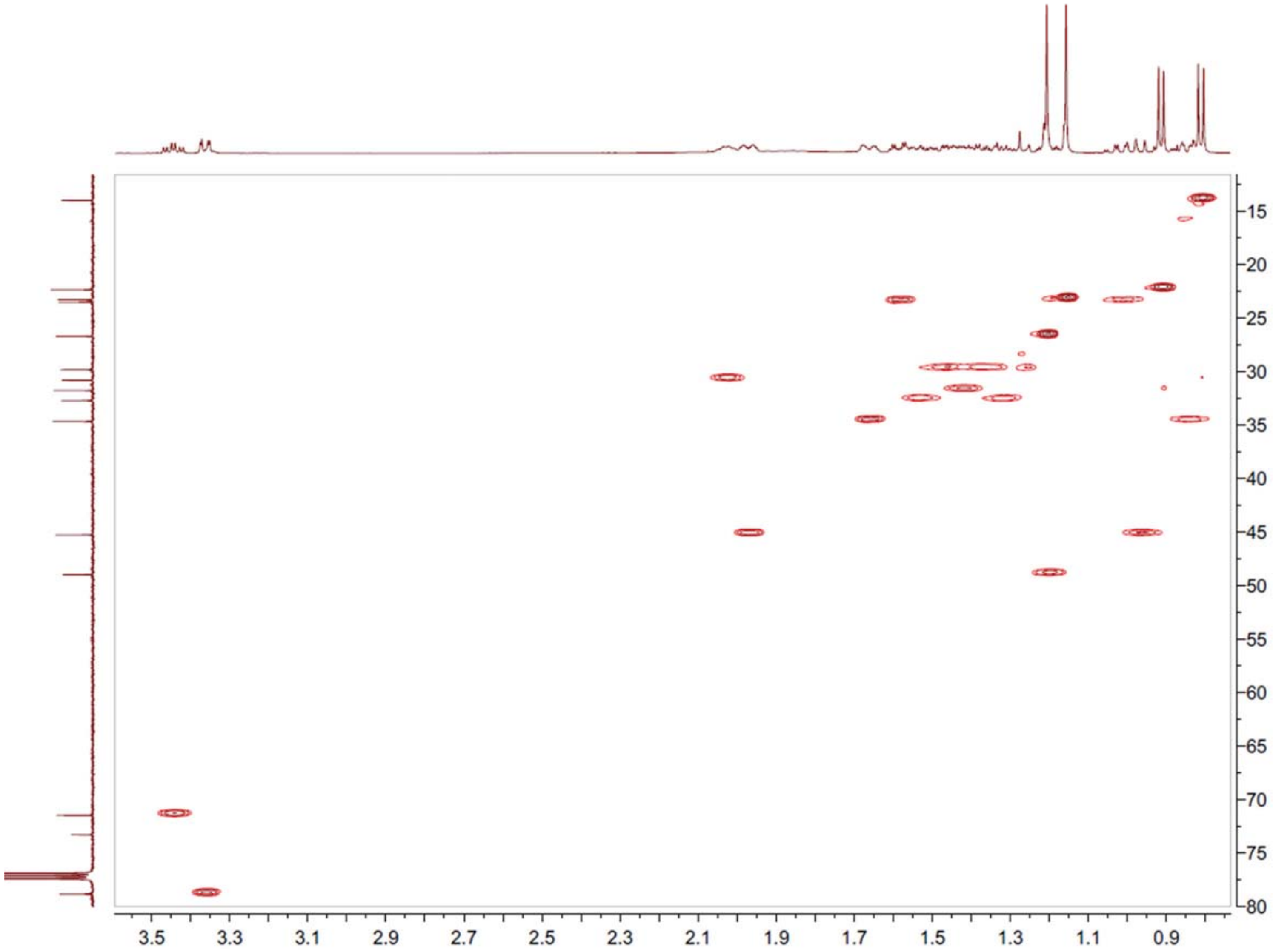

Figure S3. HSQC spectrum of compound **1** in $CDCl_3$.

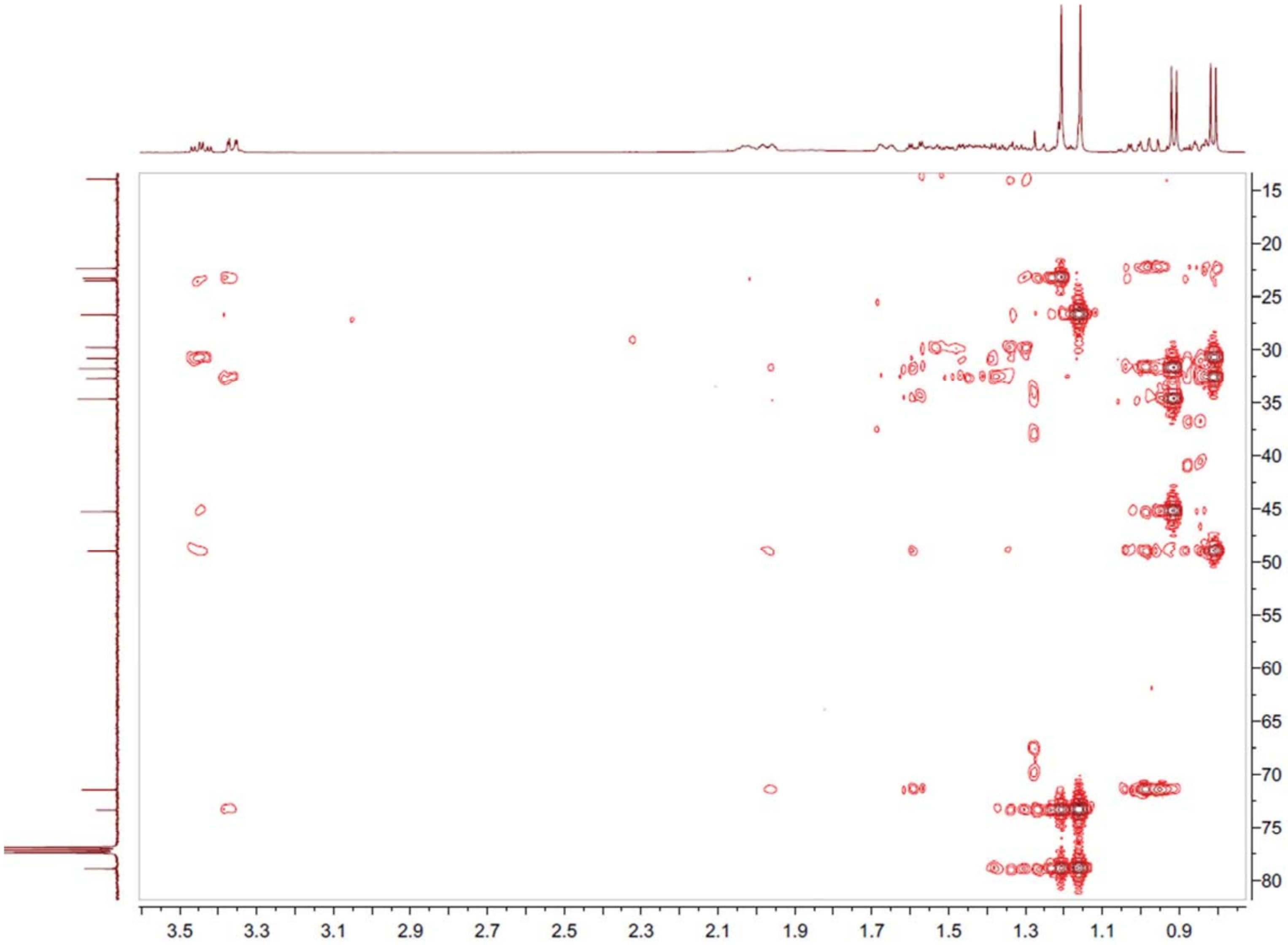

Figure S4. HMBC spectrum of compound **1** in $CDCl_3$.

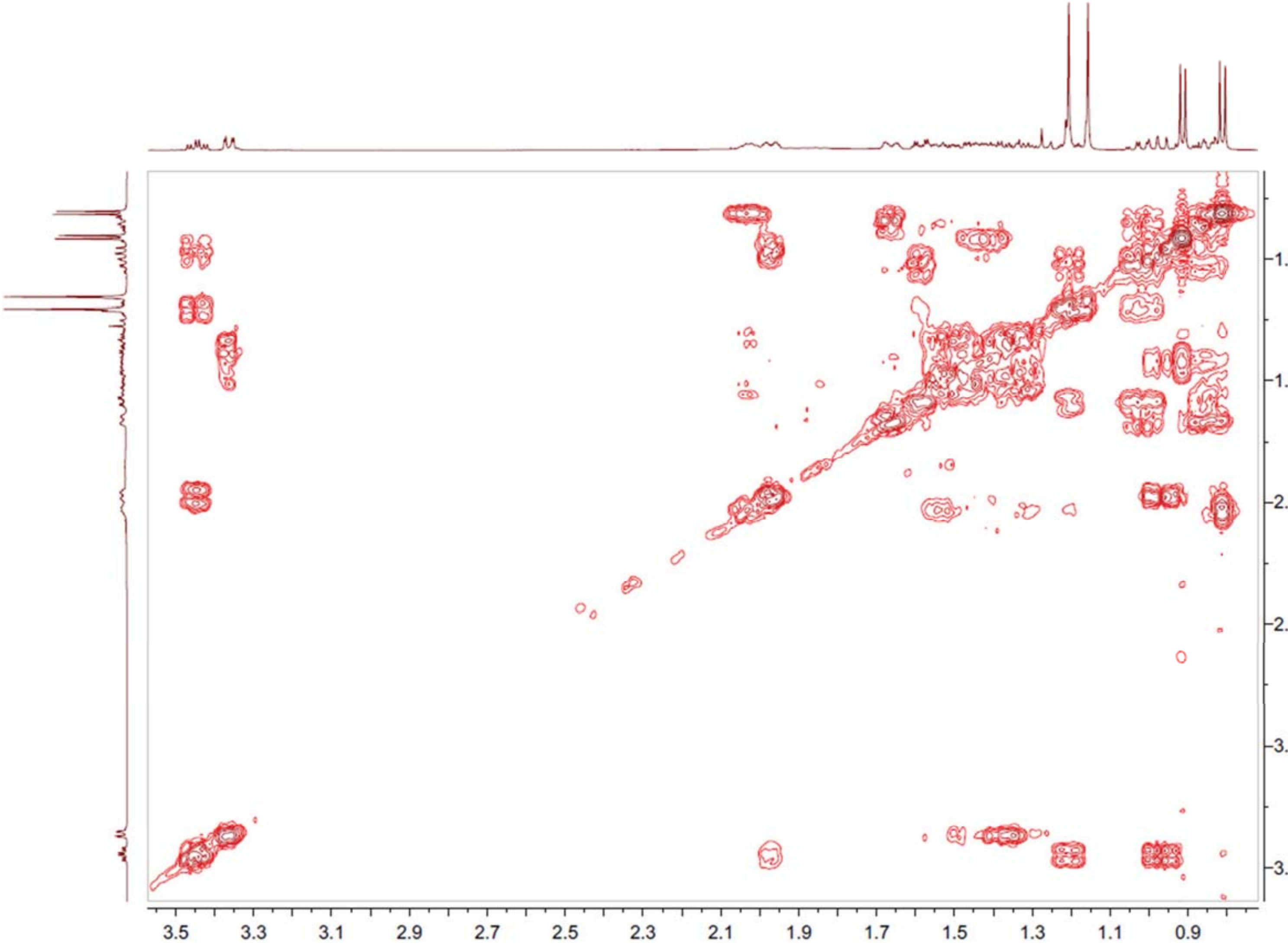

Figure S5. COSY spectrum of compound **1** in $CDCl_3$.

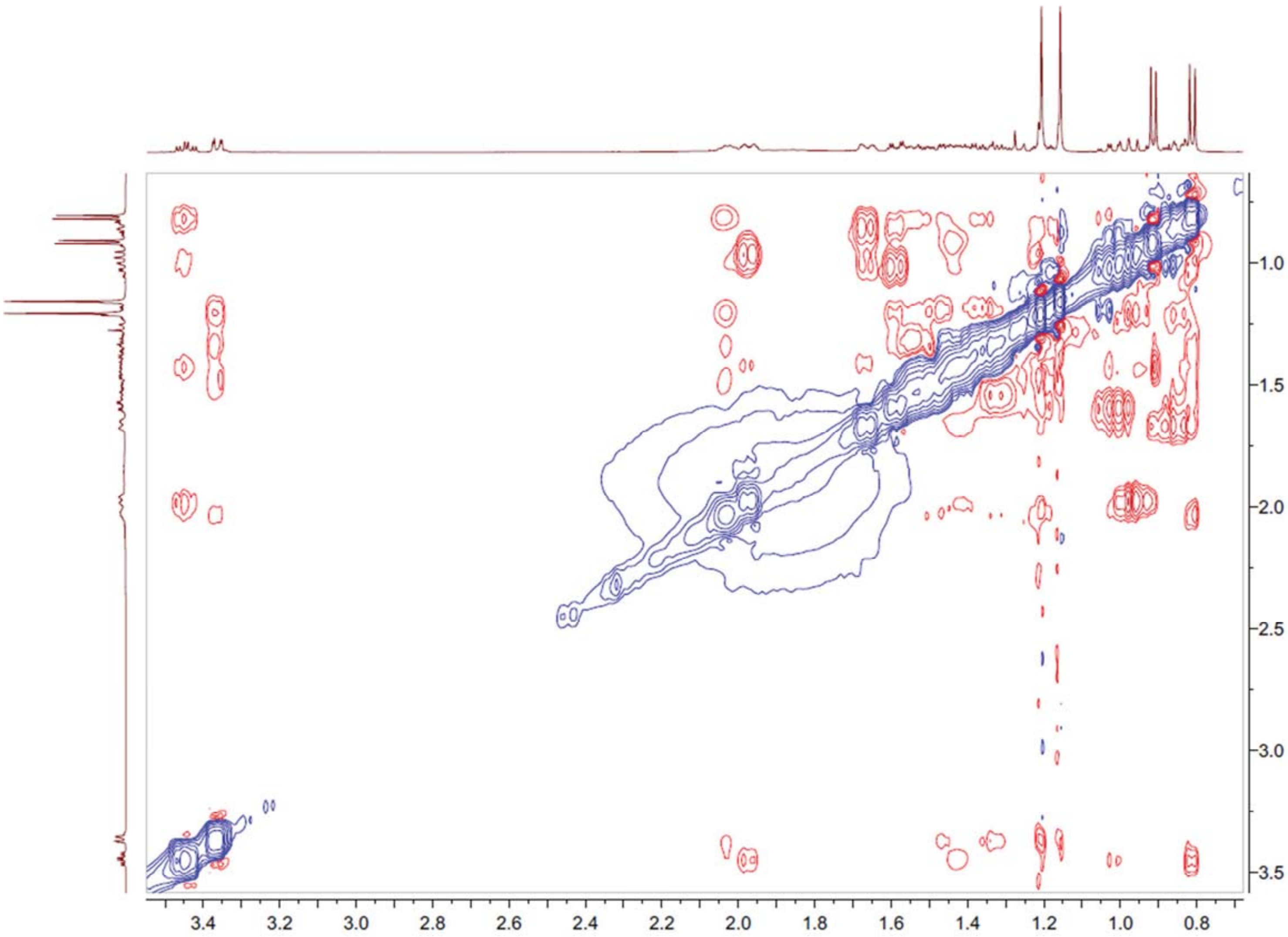

Figure S6. NOESY spectrum of compound **1** in $CDCl_3$.

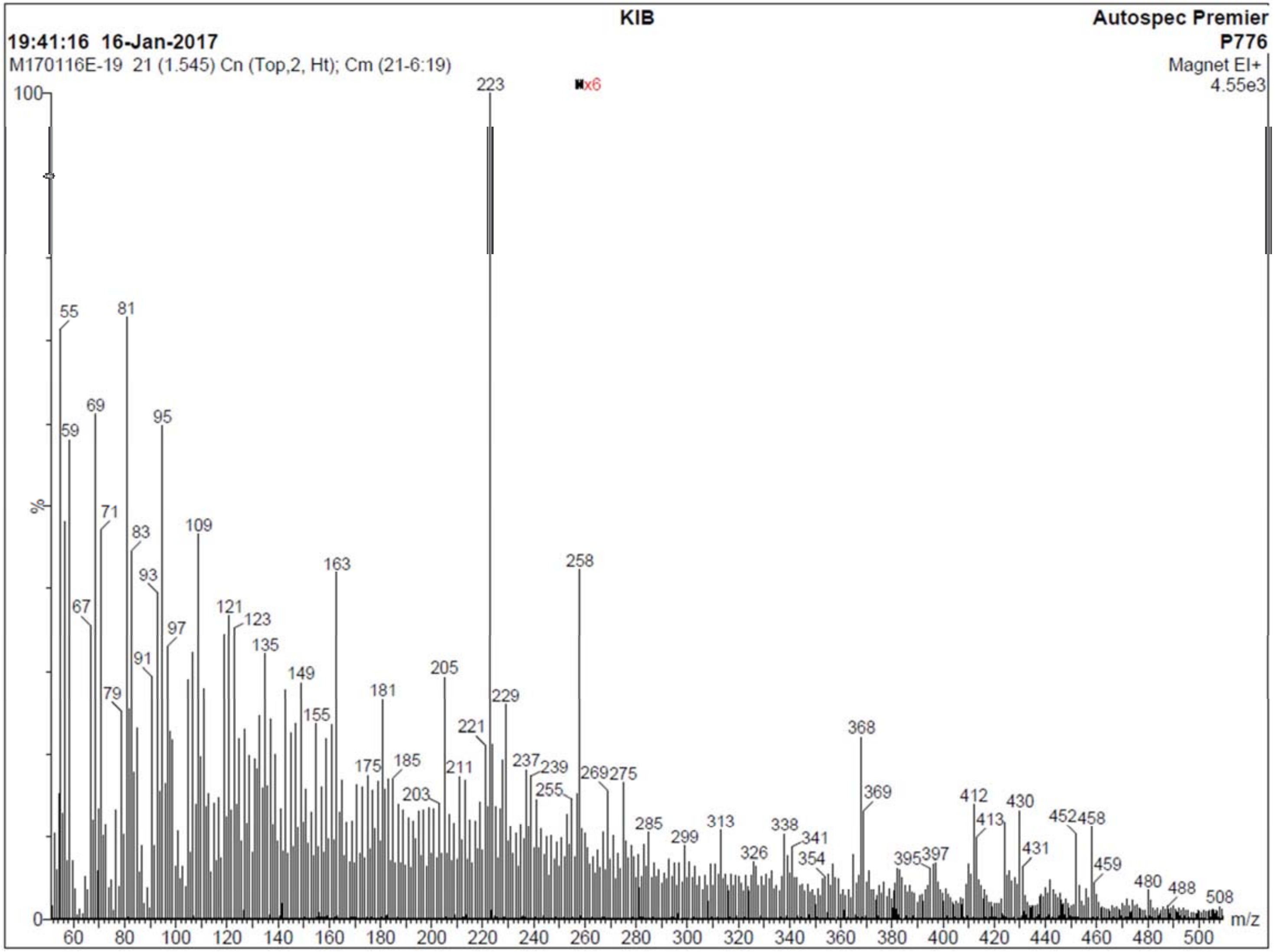

Figure S7. EIMS spectrum of compound **1**.

Single Mass Analysis

Tolerance = 10.0 PPM / DBE: min = -10.0, max = 120.0
Selected filters: None

Monoisotopic Mass, Odd and Even Electron Ions
28 formula(e) evaluated with 1 results within limits (up to 51 closest results for each mass)
Elements Used:
C: 0-200 H: 0-400 O: 0-5

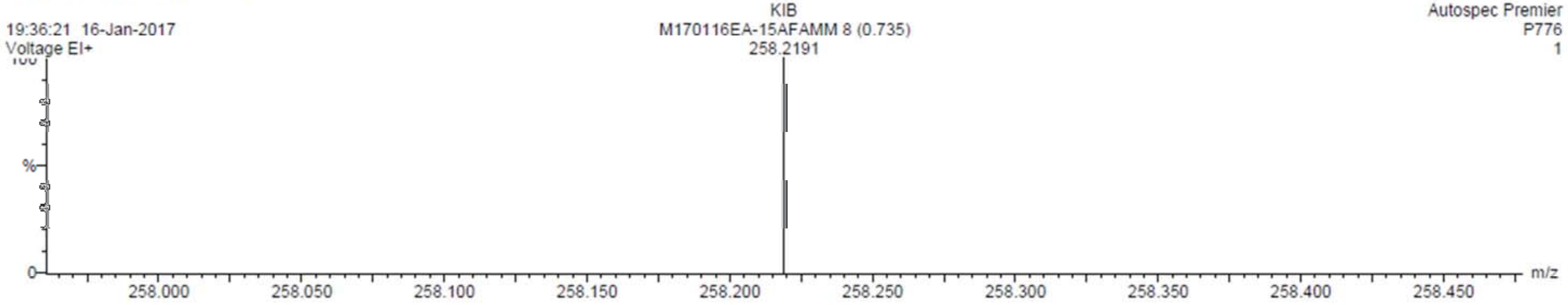

Minimum:				-10.0		
Maximum:		200.0	10.0	120.0		
Mass	Calc. Mass	mDa	PPM	DBE	i-FIT	Formula
258.2191	258.2195	-0.4	-1.5	1.0	5546026.0	C15 H30 O3

Figure S8. HREIMS spectrum of compound **1**.

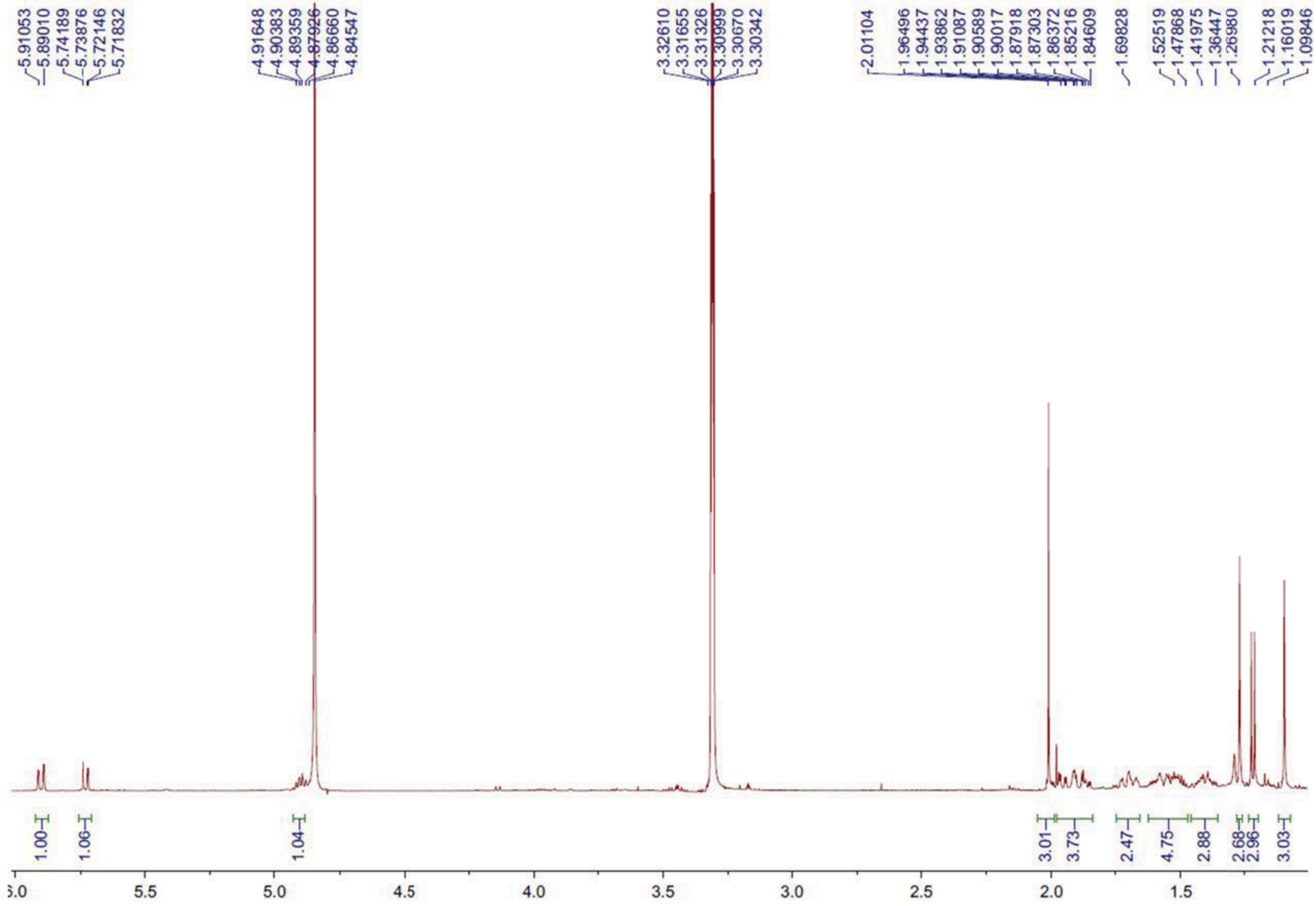

Figure S8. ^{1}H NMR spectrum of compound **2** in CD_3OD.

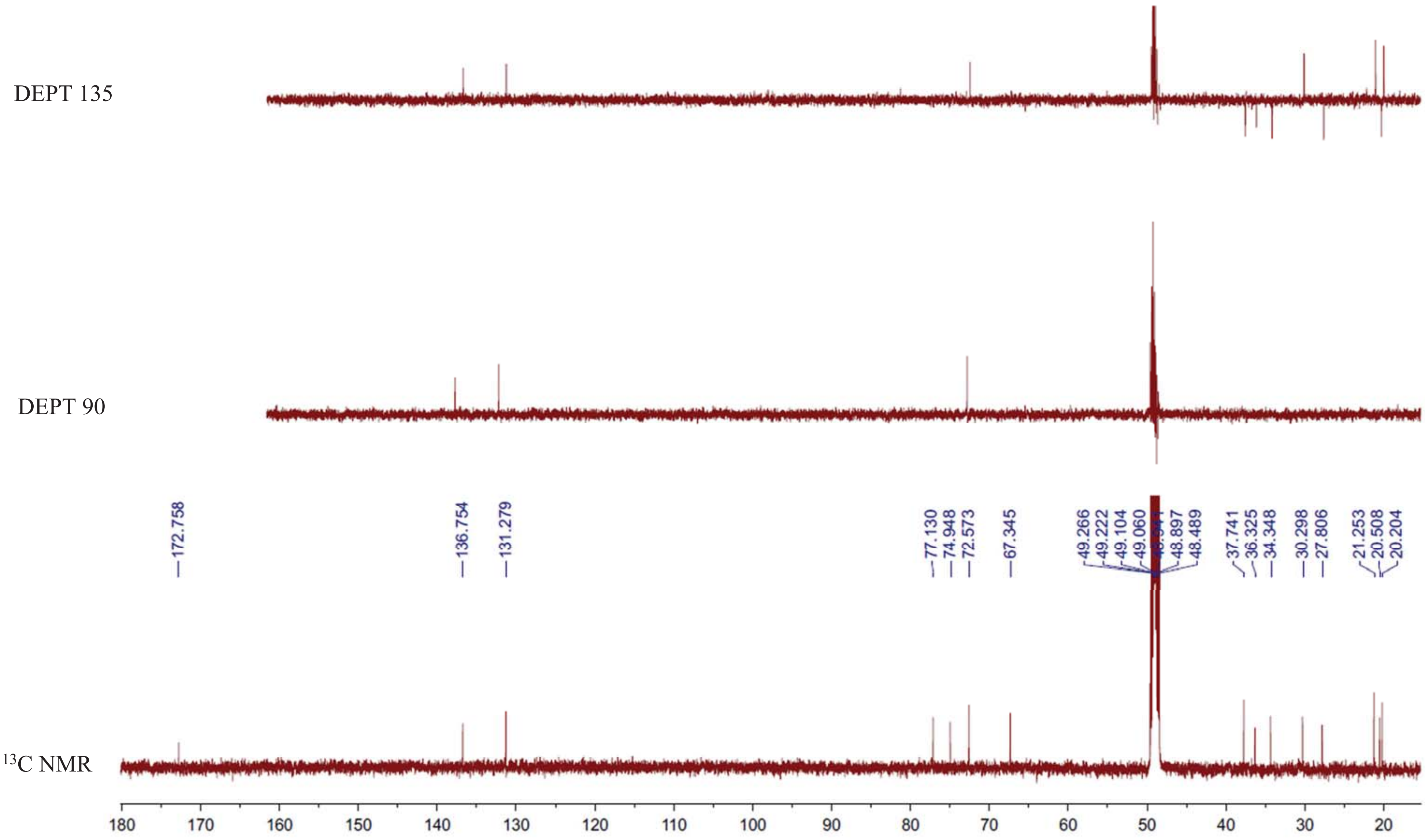

Figure S10. ^{13}C NMR and DEPT spectra of compound **2** in CD_3OD.

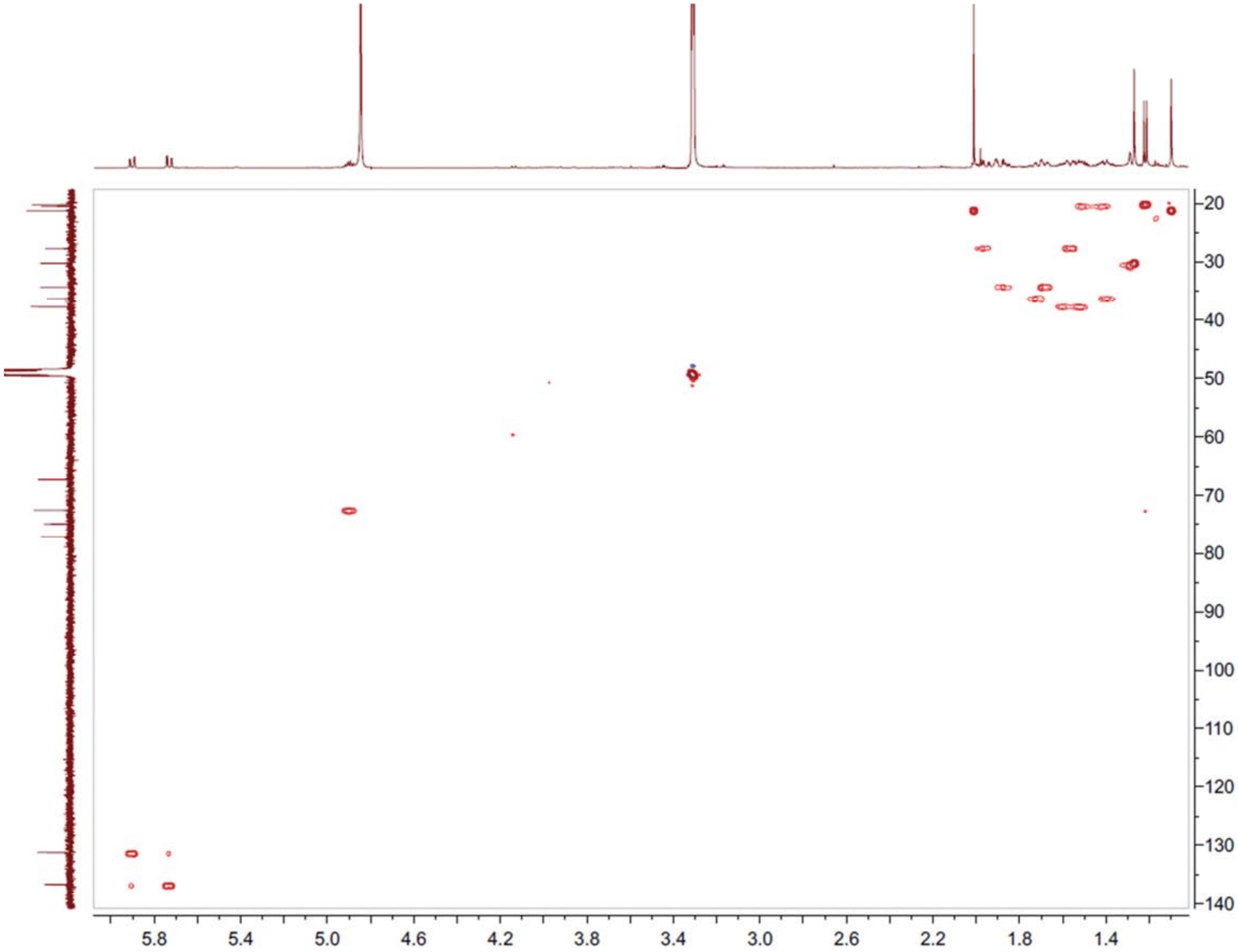

Figure S11. HSQC spectrum of compound **2** in CD_3OD.

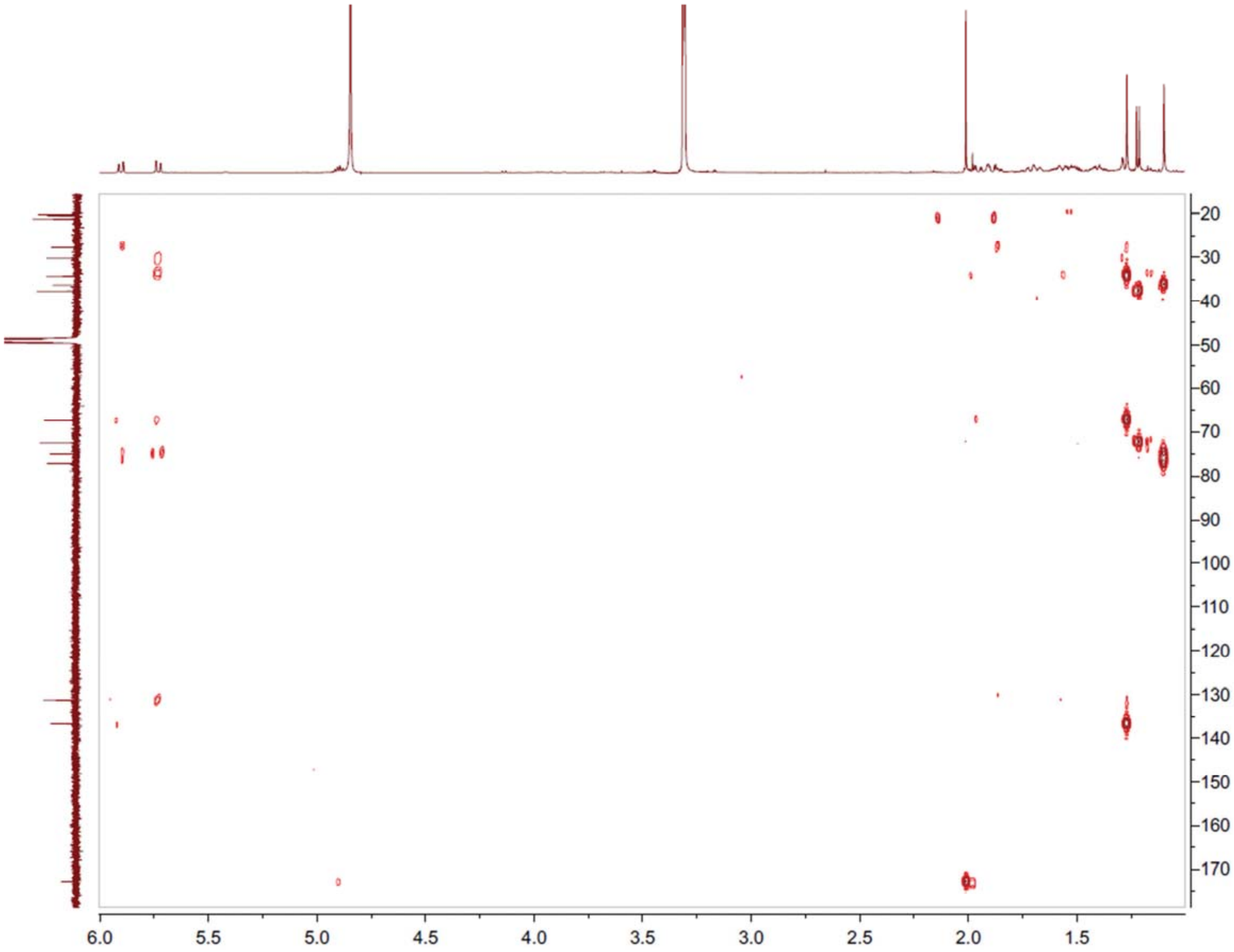

Figure S12. HMBC spectrum of compound **2** in CD_3OD.

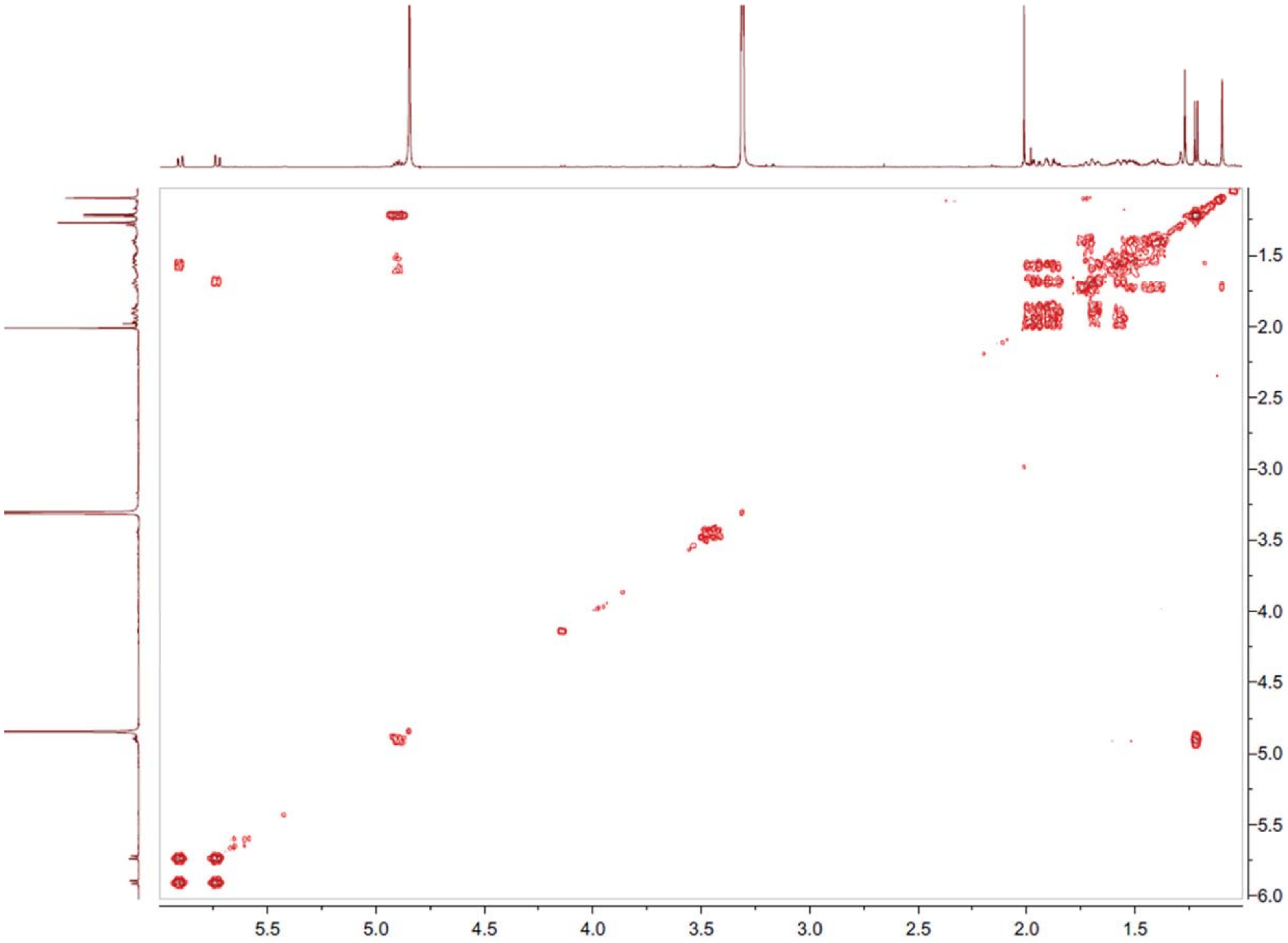

Figure S13. COSY spectrum of compound **2** in CD_3OD.

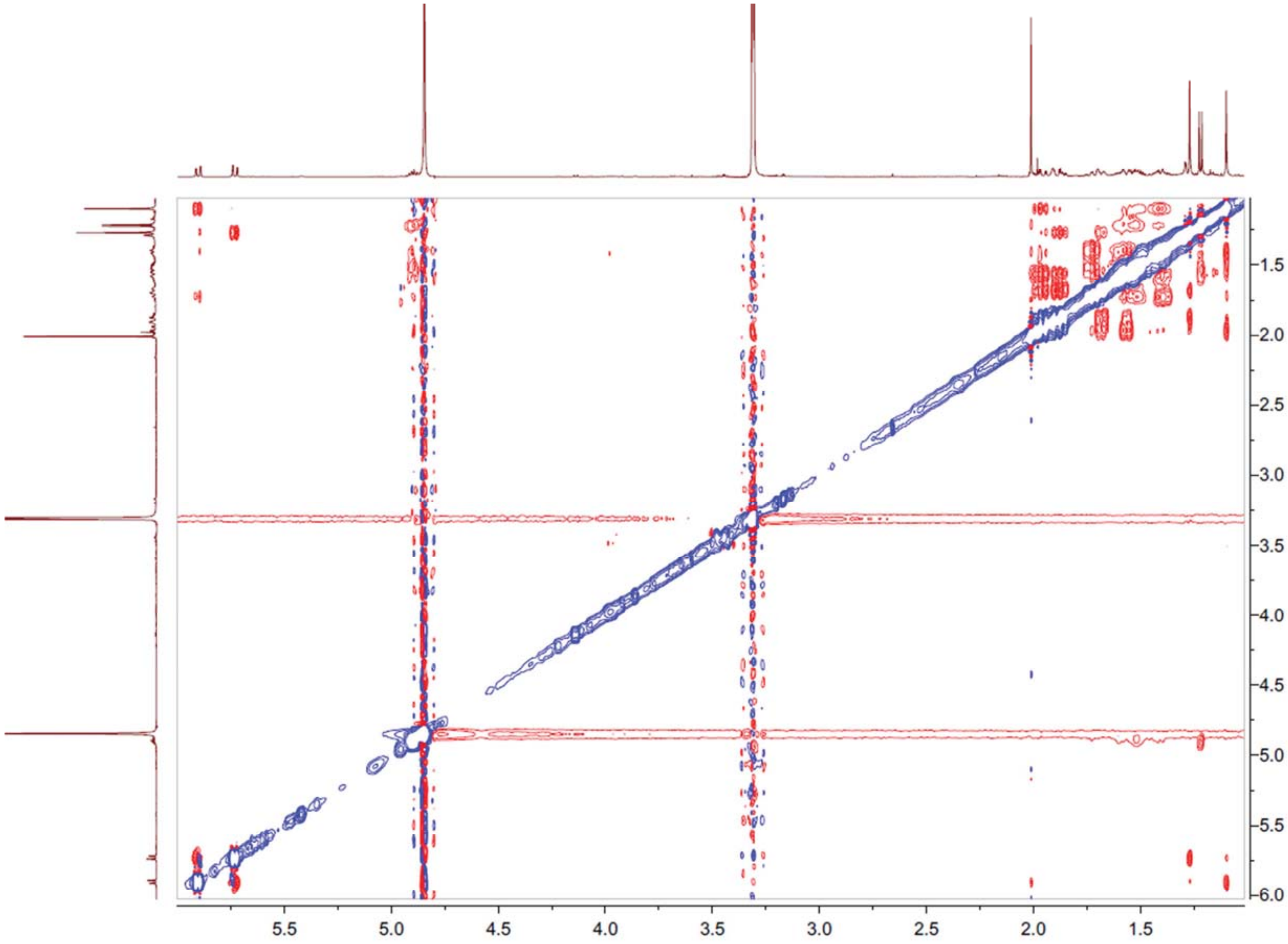

Figure S14. NOESY spectrum of compound **2** in CD_3OD.

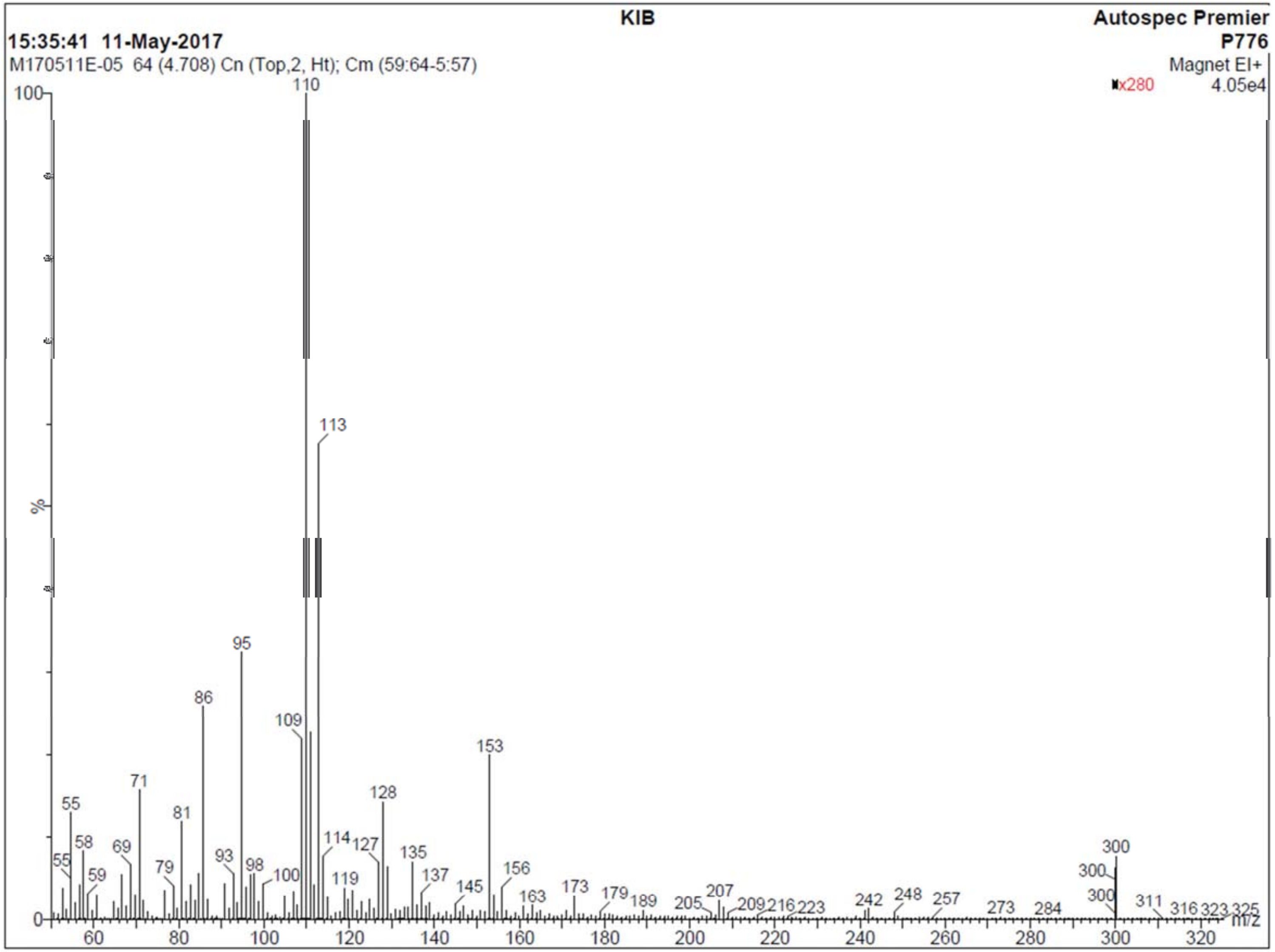

Figure S15. EIMS spectrum of compound **2**.

Single Mass Analysis
Tolerance = 10.0 PPM / DBE: min = -10.0, max = 120.0
Selected filters: None

Monoisotopic Mass, Odd and Even Electron Ions
15 formula(e) evaluated with 1 results within limits (up to 51 closest results for each mass)
Elements Used:
C: 0-200 H: 0-400 O: 4-6

17:38:53 11-May-2017 KIB M170511EA-05AFAMMA 25 (2.296) Autospec Premier P776 1
Voltage EI+

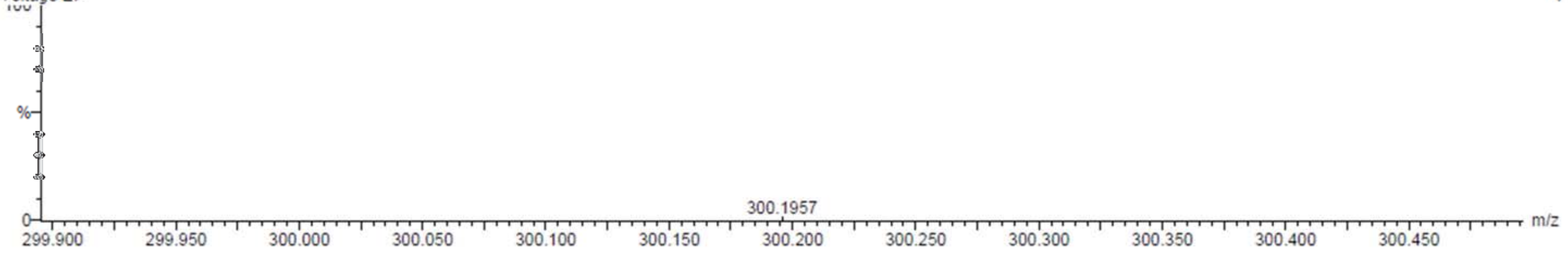

Minimum:				-10.0		
Maximum:		10.0	10.0	120.0		
Mass	Calc. Mass	mDa	PPM	DBE	i-FIT	Formula
300.1957	300.1937	2.0	6.7	3.0	5546026.0	C16 H28 O5

Figure S16. HREIMS spectrum of compound **2**.

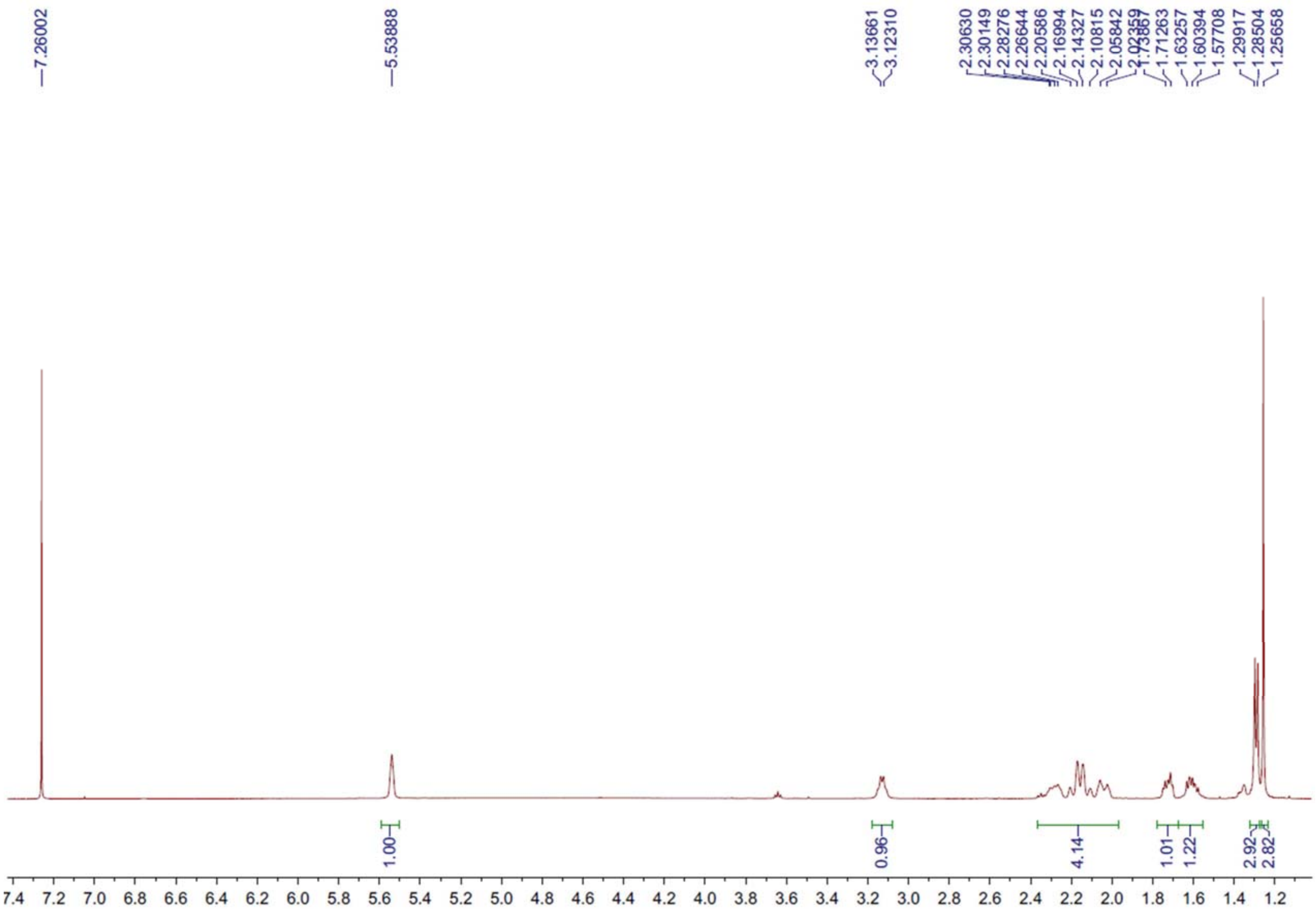

Figure S17. ^{1}H NMR spectrum of compound **3** in $CDCl_3$.

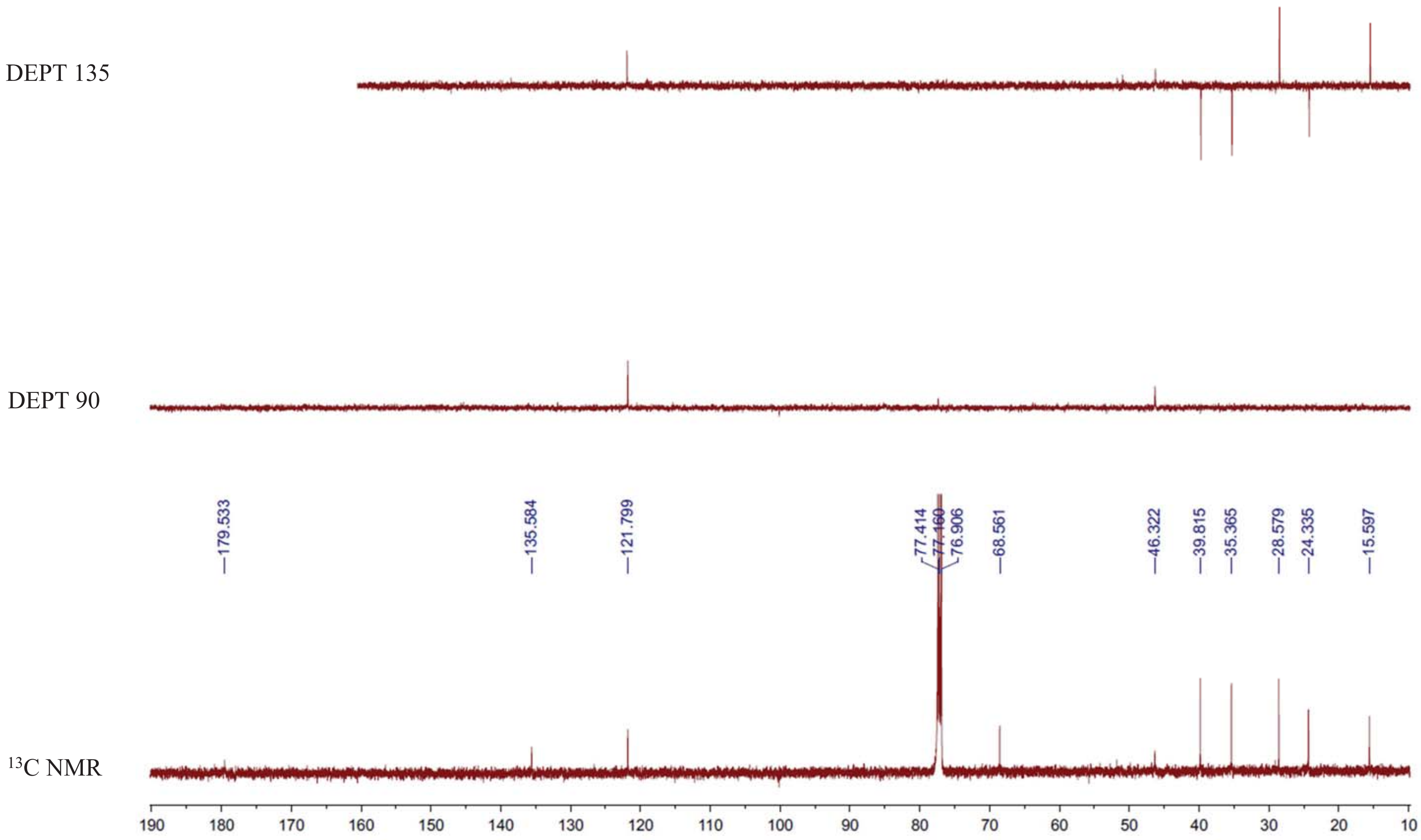

Figure S18. ^{13}C NMR and DEPT spectra of compound **3** in $CDCl_3$.

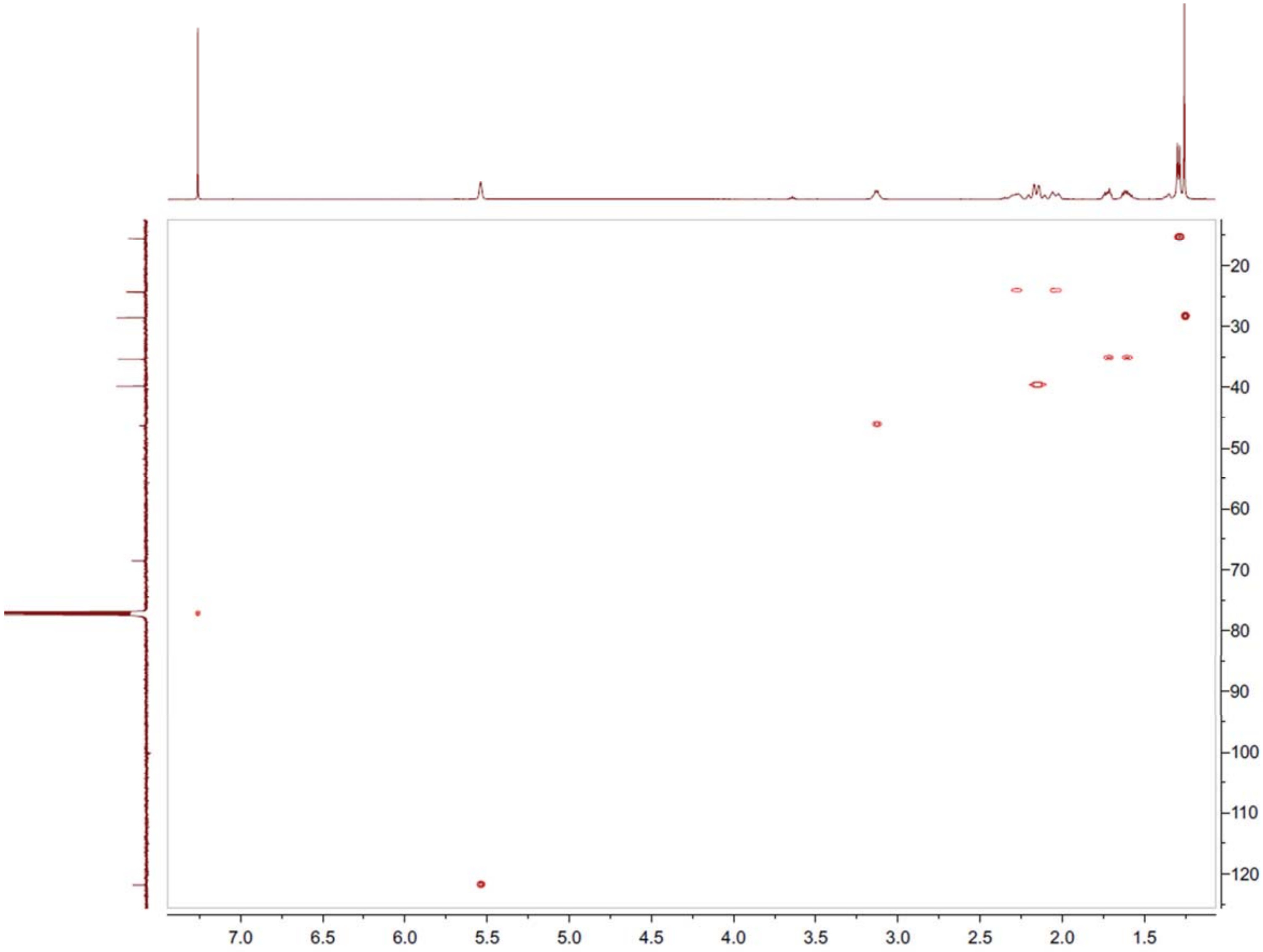

Figure S19. HSQC spectrum of compound **3** in $CDCl_3$.

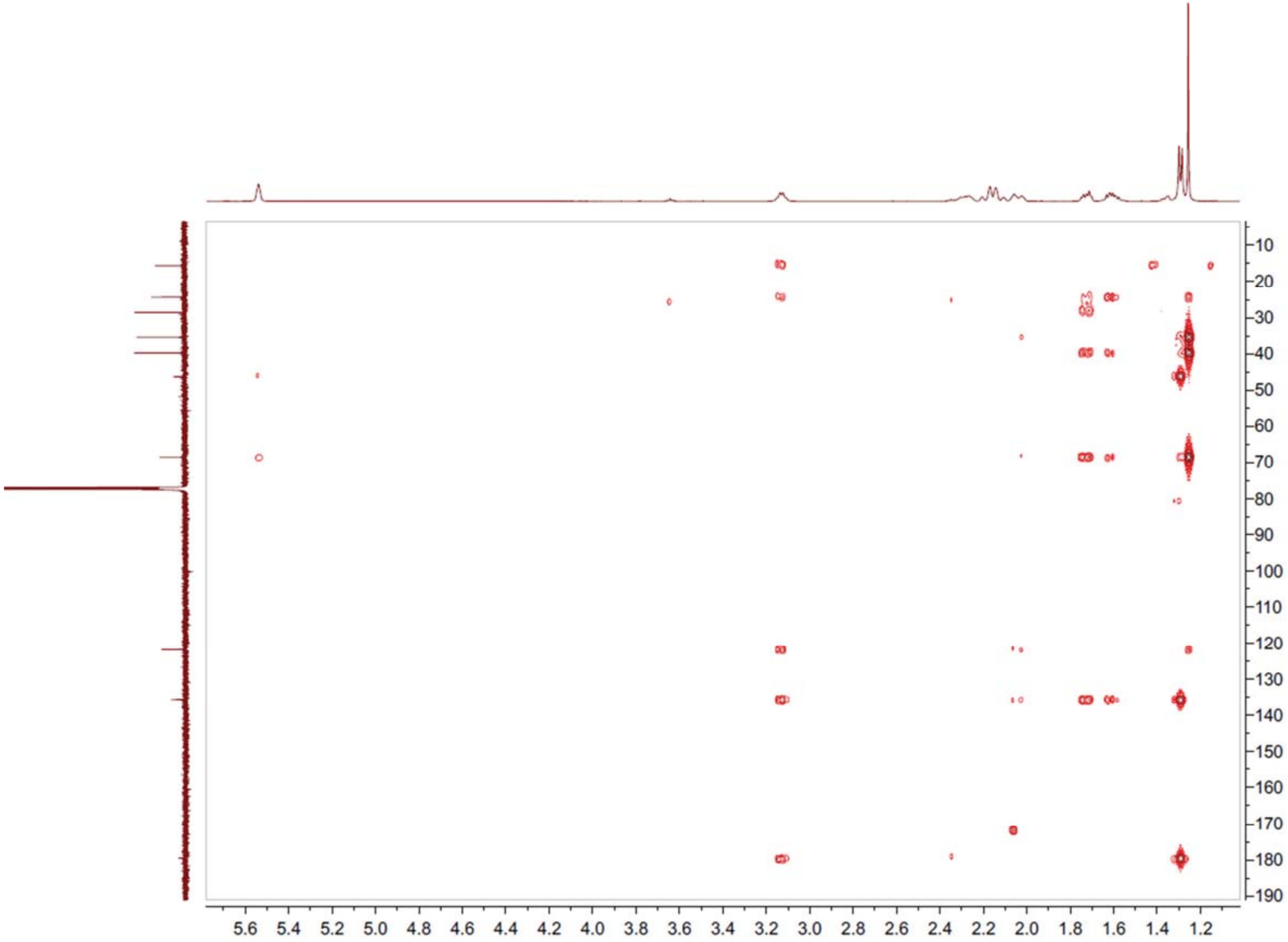

Figure S20. HMBC spectrum of compound **3** in $CDCl_3$.

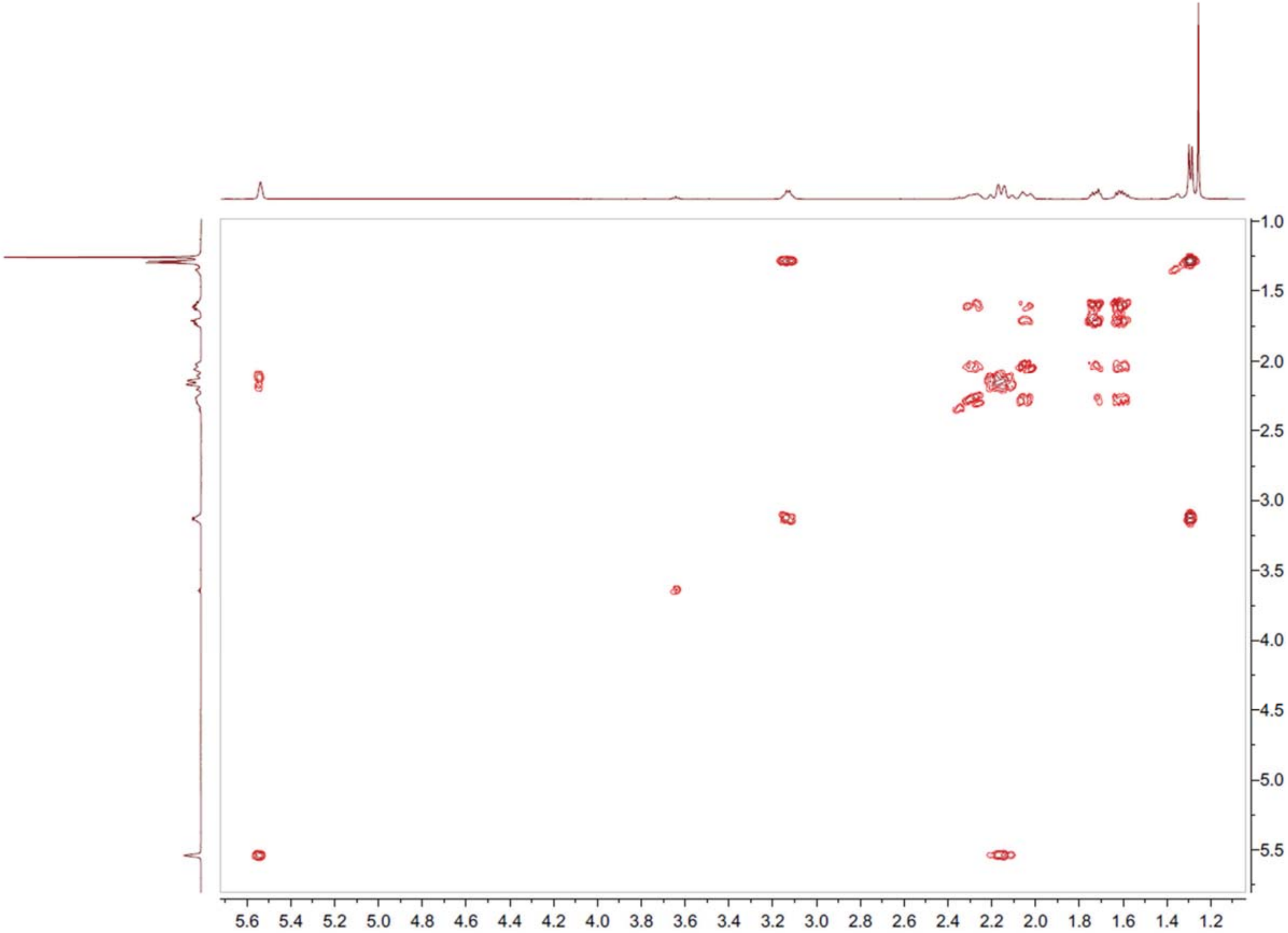

Figure S21. COSY spectrum of compound **3** in $CDCl_3$.

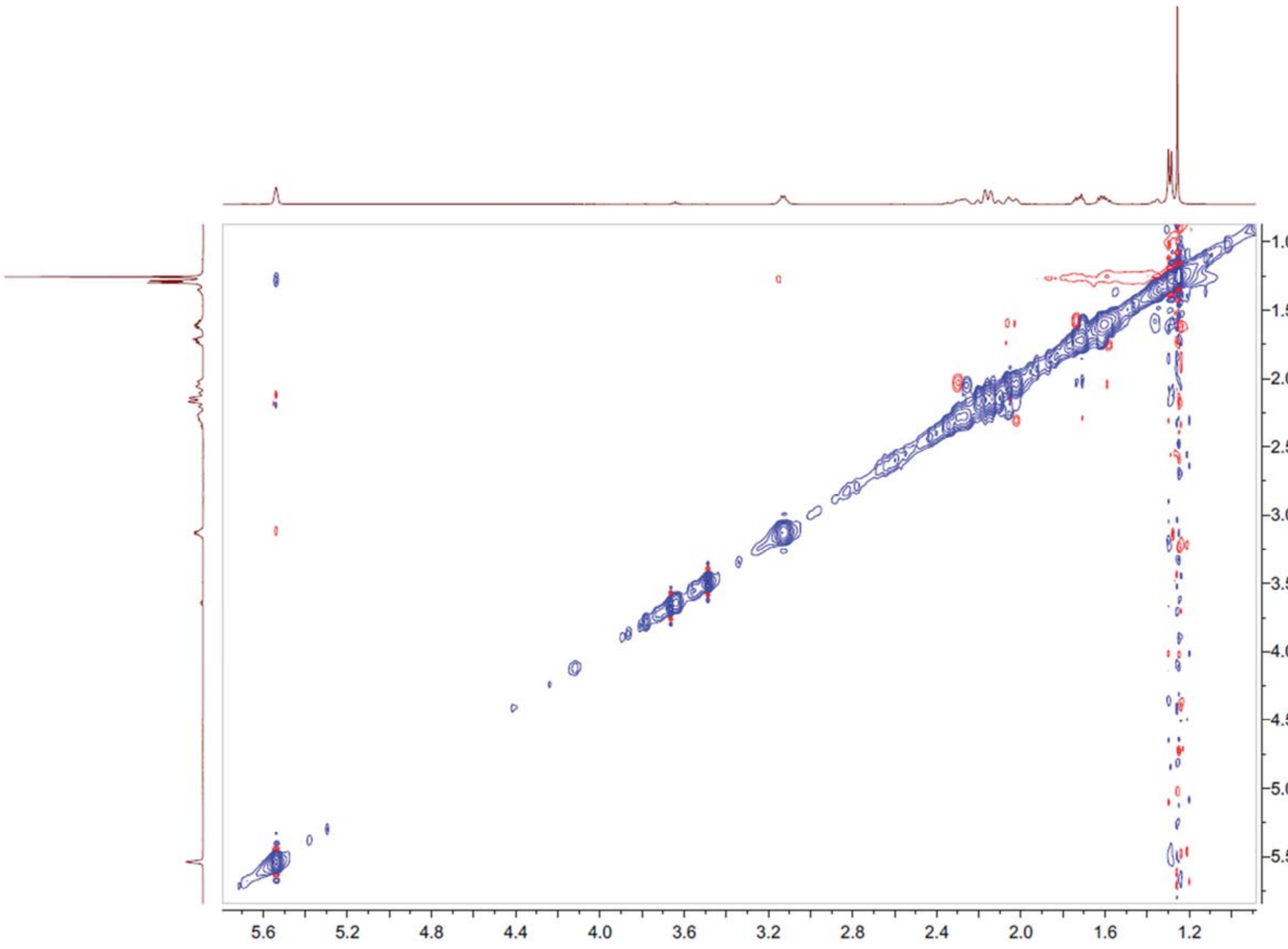

Figure S22. NOESY spectrum of compound **3** in $CDCl_3$.

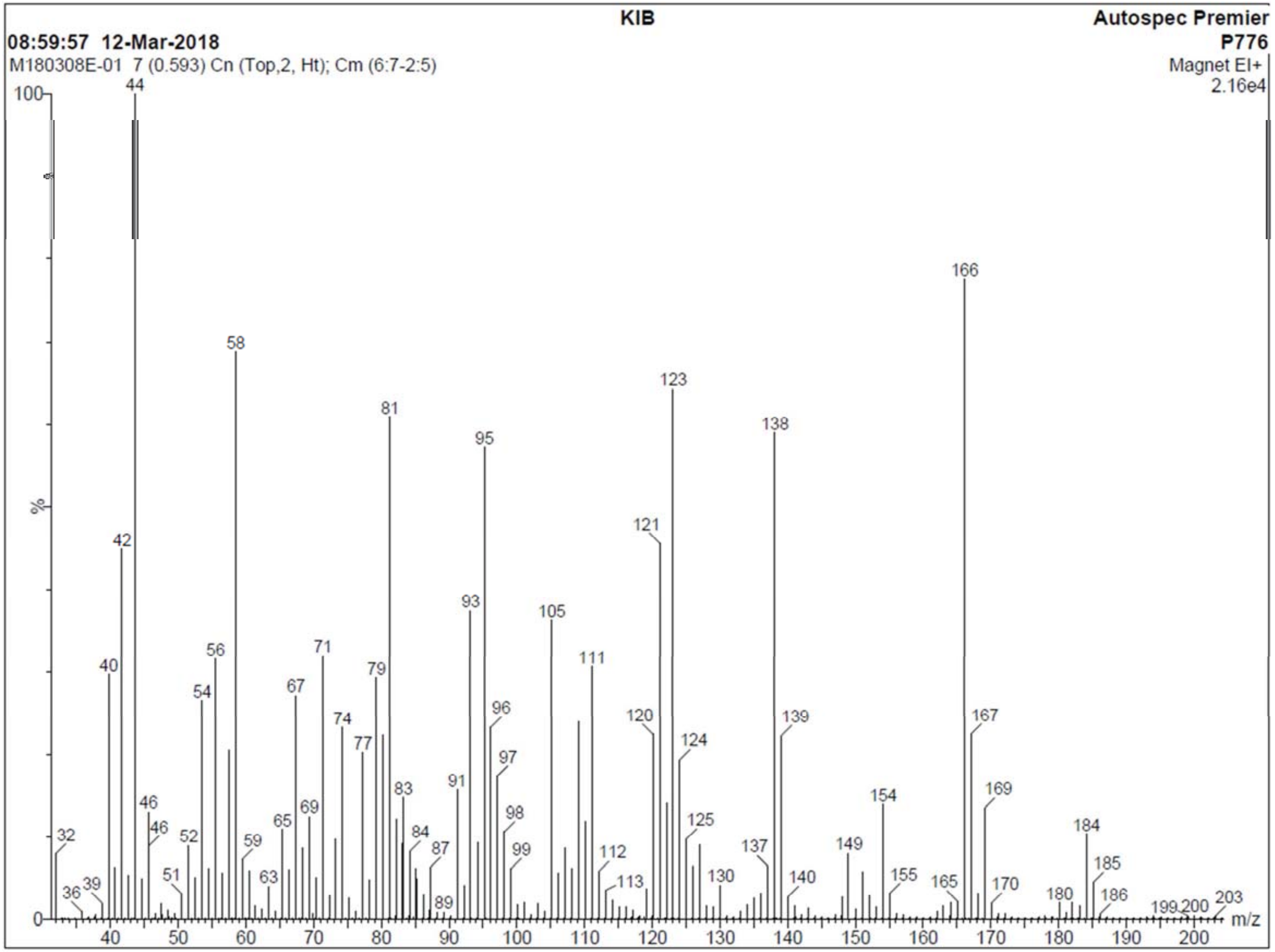

Figure S23. EIMS spectrum of compound **3**.

Single Mass Analysis
Tolerance = 10.0 PPM / DBE: min = -10.0, max = 120.0
Selected filters: None

Monoisotopic Mass, Odd and Even Electron Ions
12 formula(e) evaluated with 1 results within limits (up to 51 closest results for each mass)
Elements Used:
C: 0-200 H: 0-400 O: 2-4

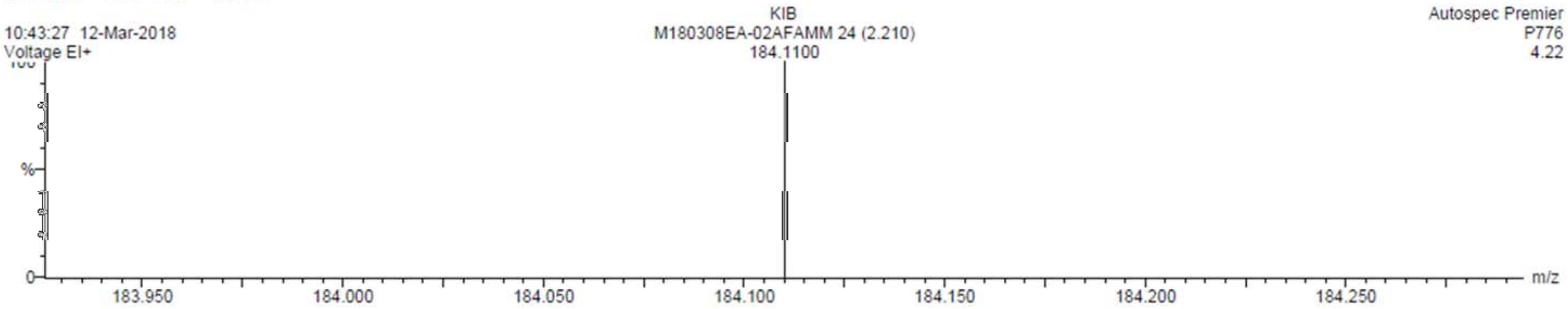

Minimum:				-10.0		
Maximum:		200.0	10.0	120.0		
Mass	Calc. Mass	mDa	PPM	DBE	i-FIT	Formula
184.1100	184.1099	0.1	0.5	3.0	5546025.5	C10 H16 O3

Figure S24. HREIMS spectrum of compound **3**.

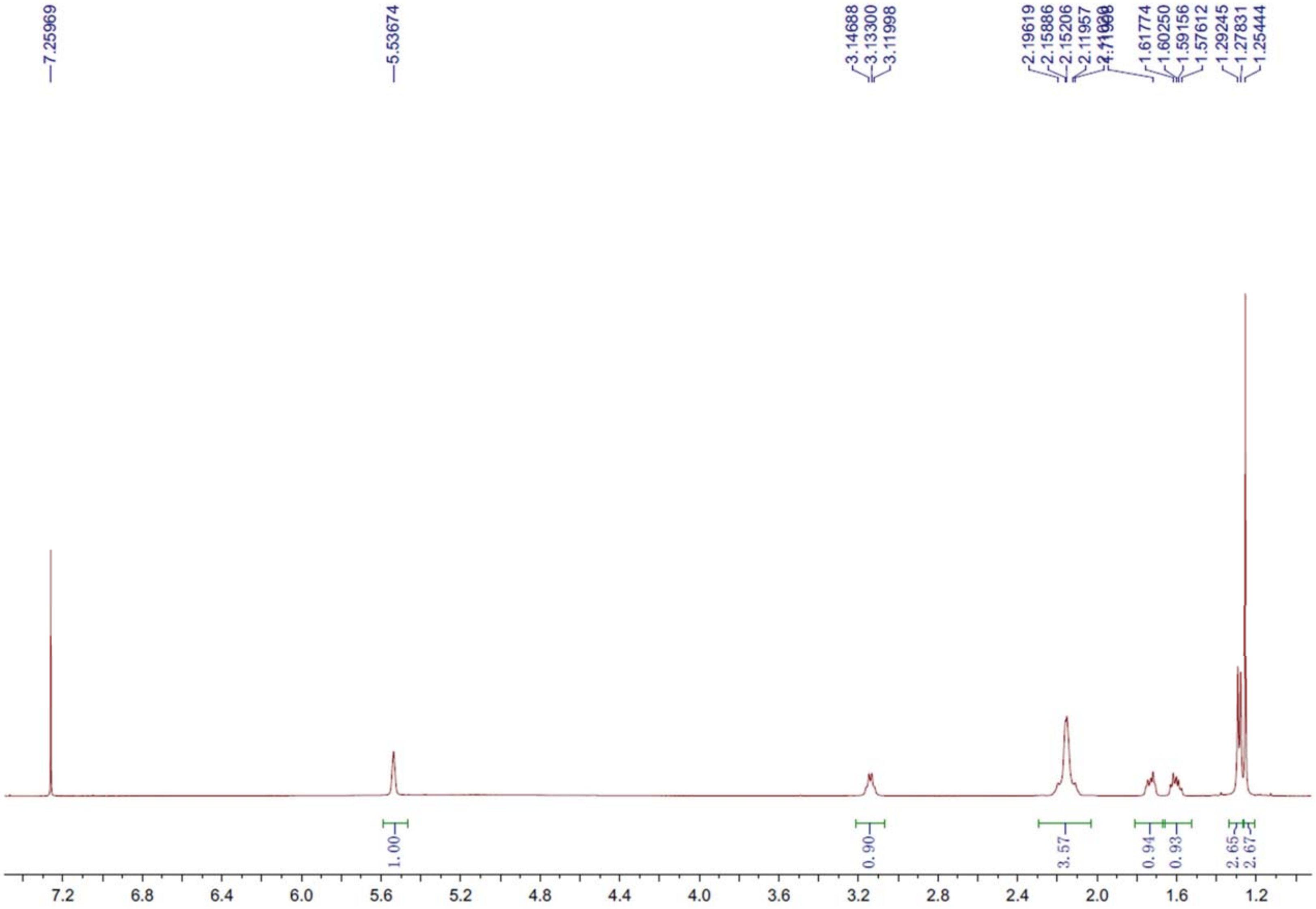

Figure S25. ^{1}H NMR spectrum of compound **4** in $CDCl_3$.

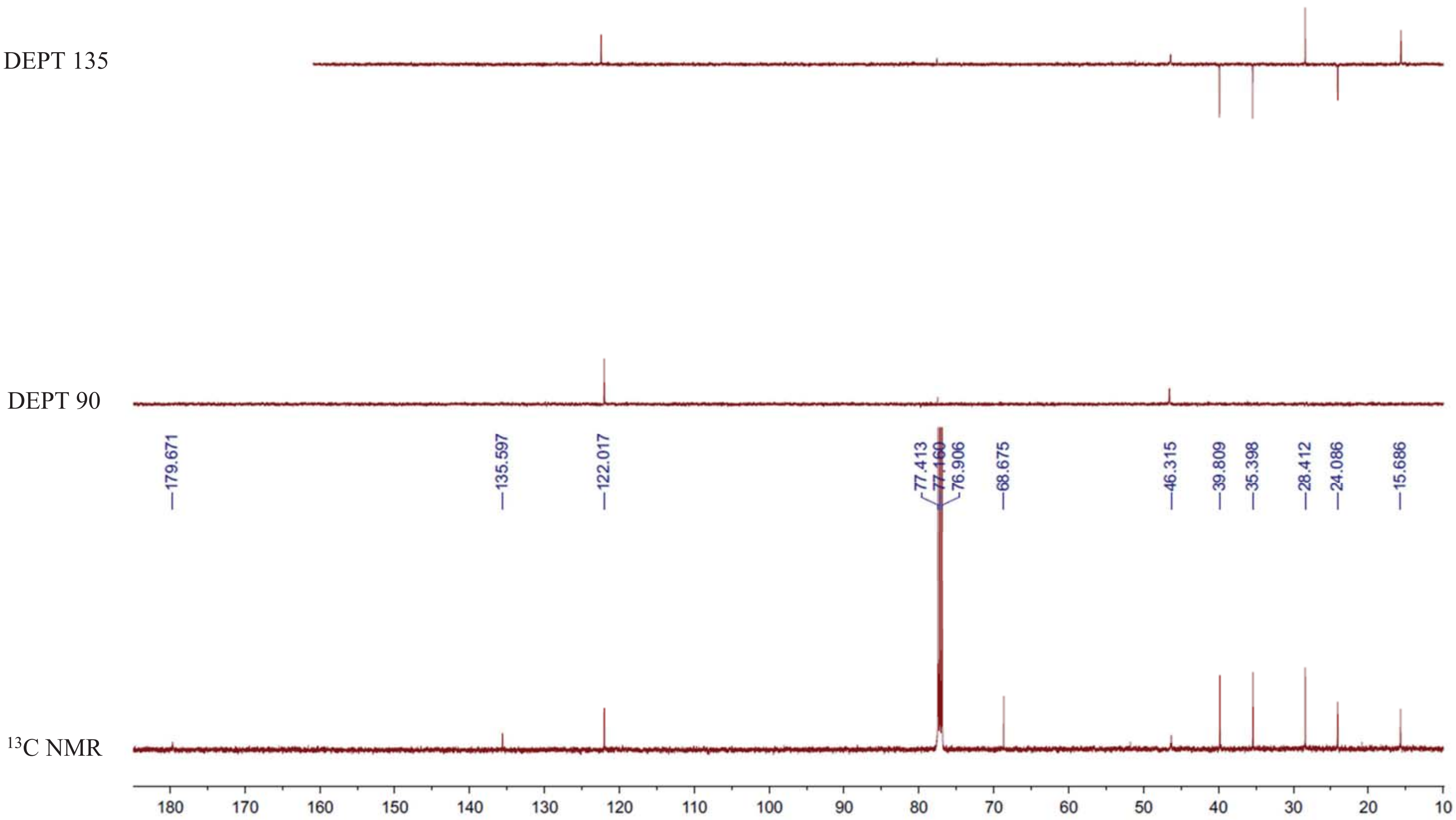

Figure S26. ^{13}C NMR and DEPT spectra of compound **4** in $CDCl_3$.

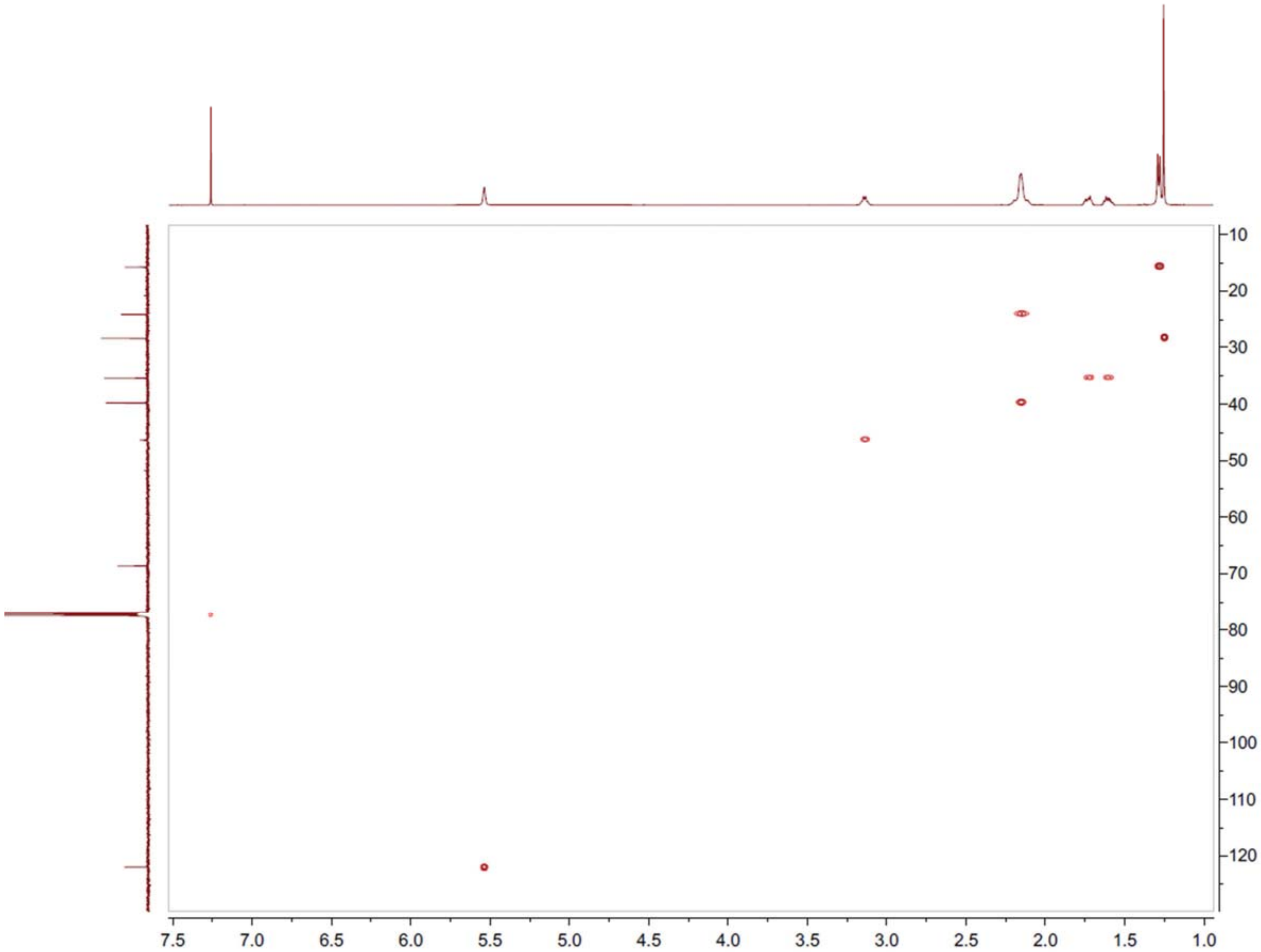

Figure S27. HSQC spectrum of compound **4** in $CDCl_3$.

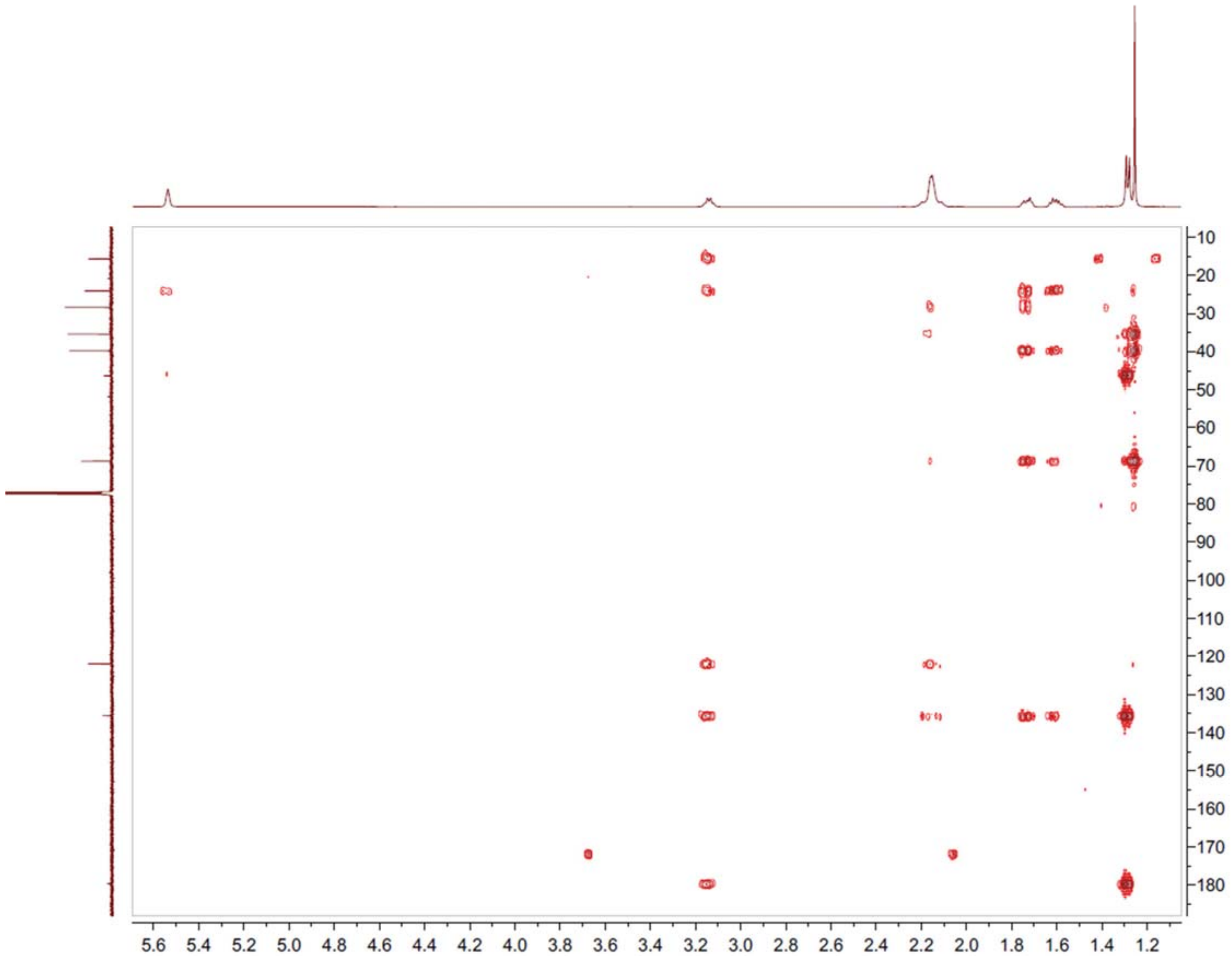

Figure S28. HMBC spectrum of compound **4** in $CDCl_3$.

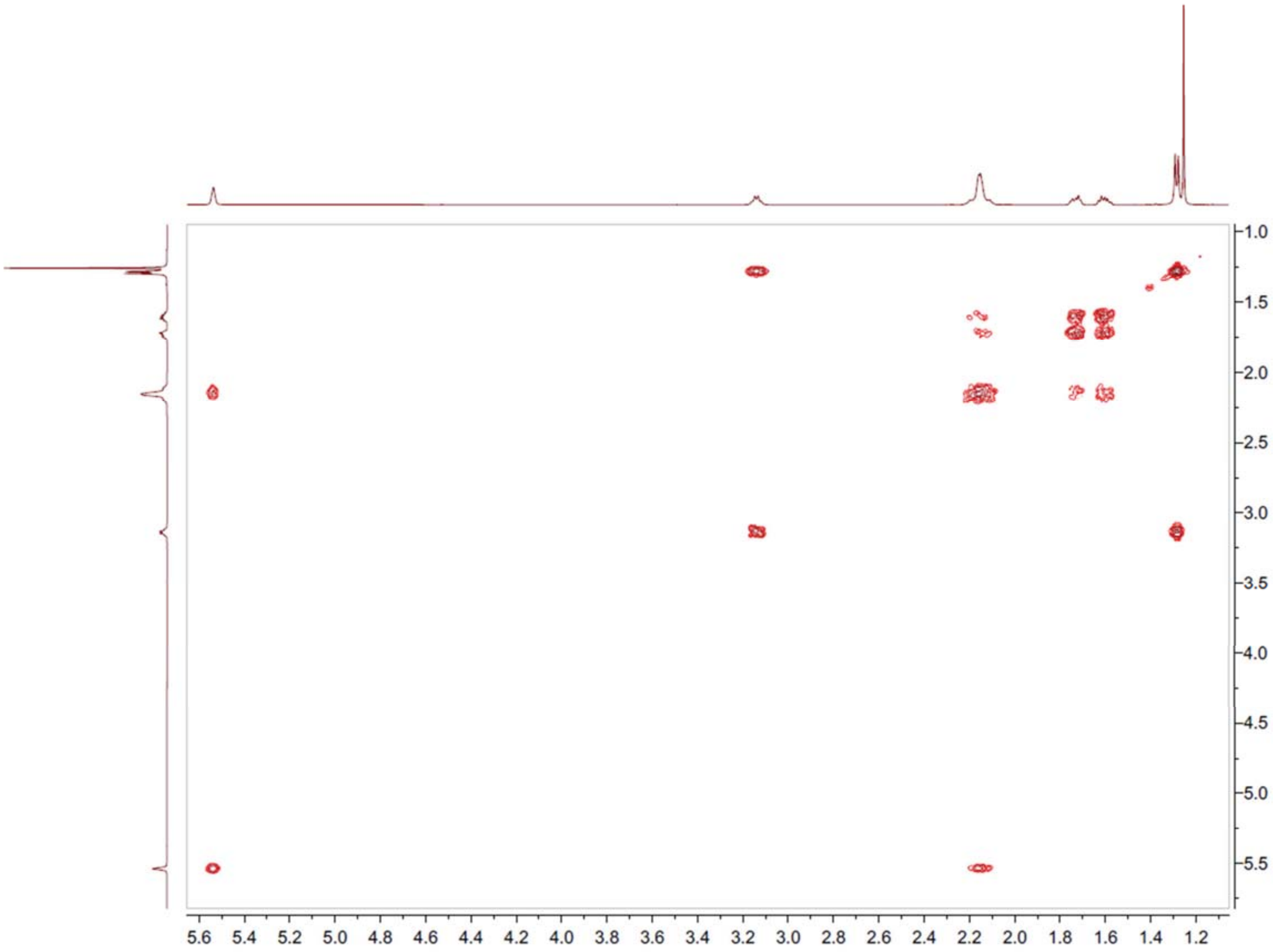

Figure S29. COSY spectrum of compound **4** in $CDCl_3$.

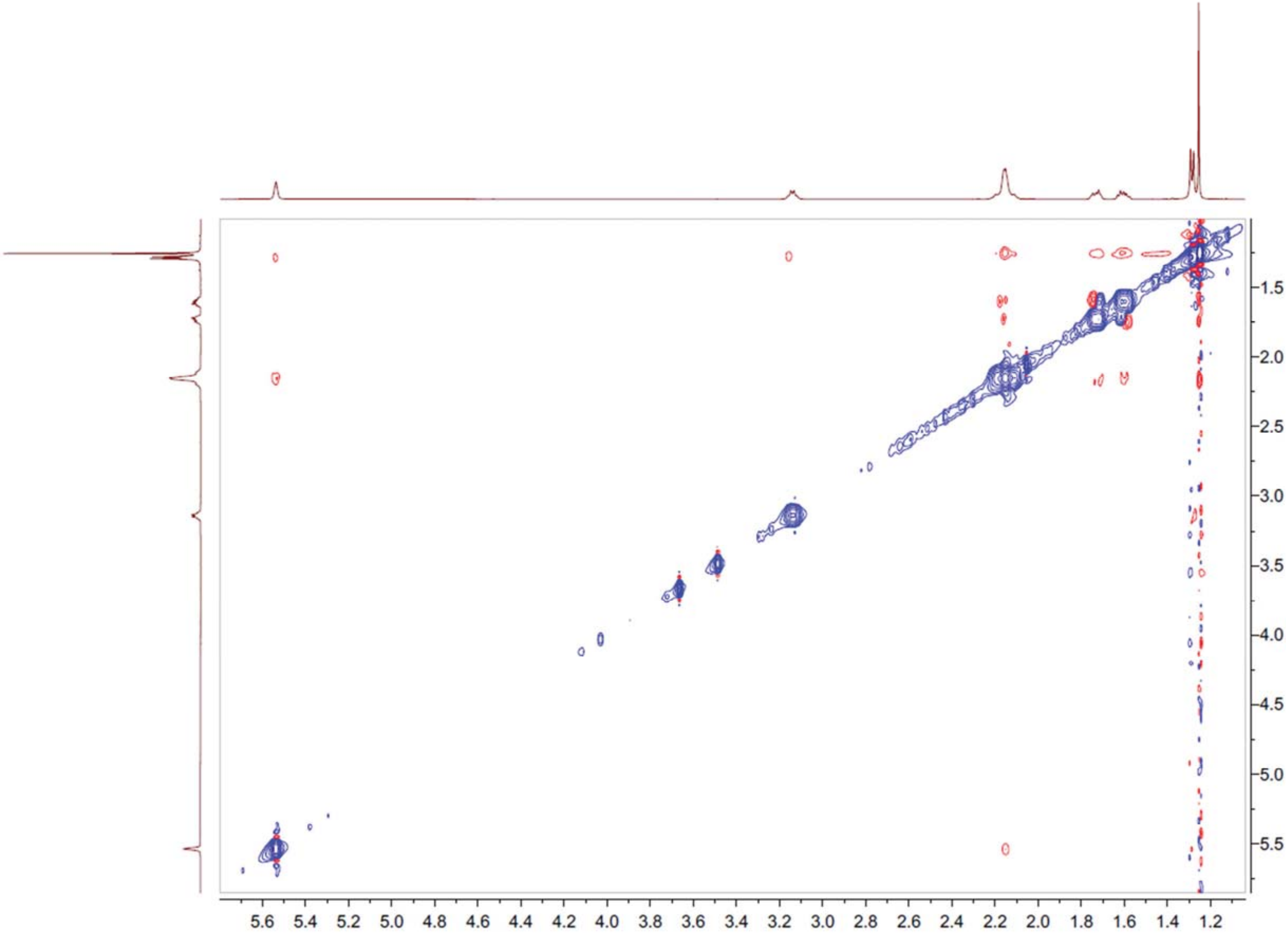

Figure S30. NOESY spectrum of compound **4** in $CDCl_3$.

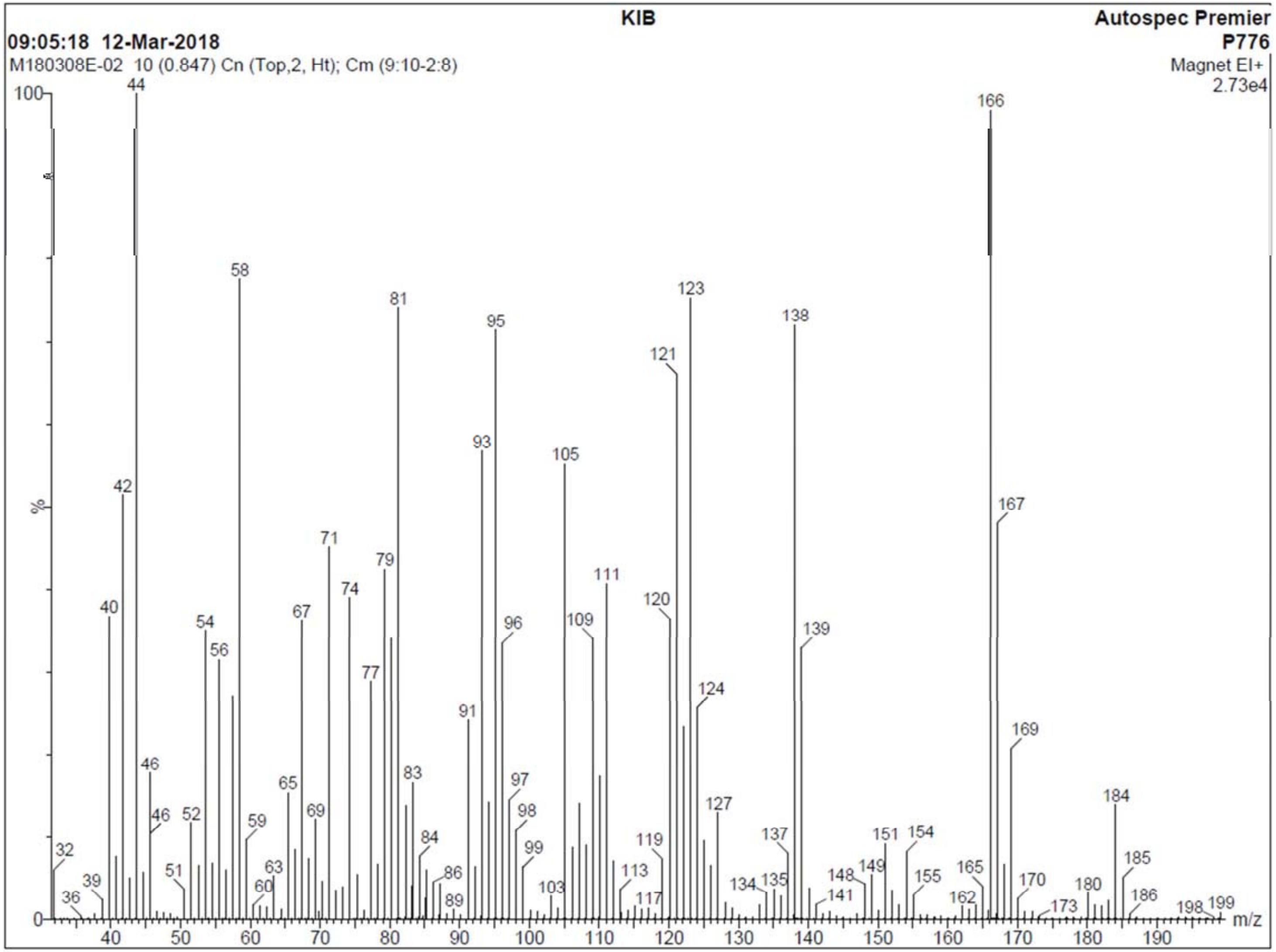

Figure S31. EIMS spectrum of compound **4**.

Single Mass Analysis
Tolerance = 10.0 PPM / DBE: min = -10.0, max = 120.0
Selected filters: None

Monoisotopic Mass, Odd and Even Electron Ions
12 formula(e) evaluated with 1 results within limits (up to 51 closest results for each mass)
Elements Used:
C: 0-200 H: 0-400 O: 2-4

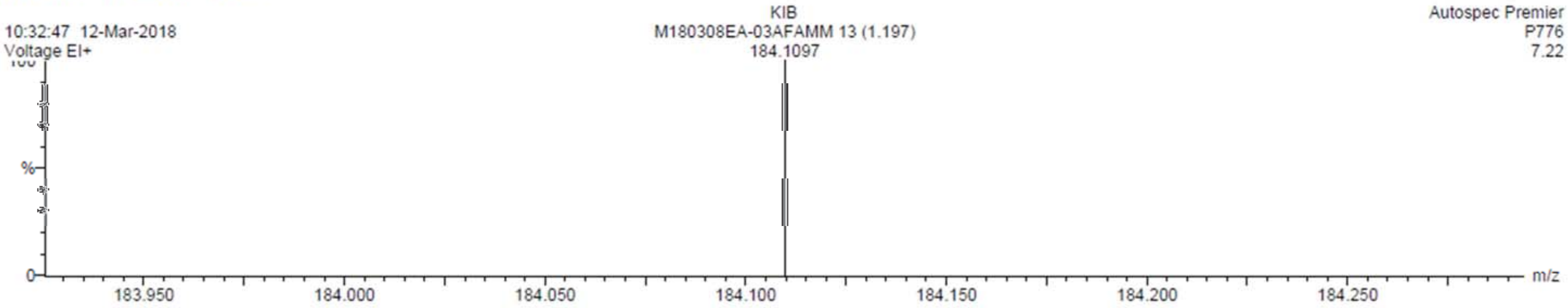

Minimum:				-10.0		
Maximum:		200.0	10.0	120.0		
Mass	Calc. Mass	mDa	PPM	DBE	i-FIT	Formula
184.1097	184.1099	-0.2	-1.1	3.0	5546025.5	C10 H16 O3

Figure S32. HREIMS spectrum of compound **4**.

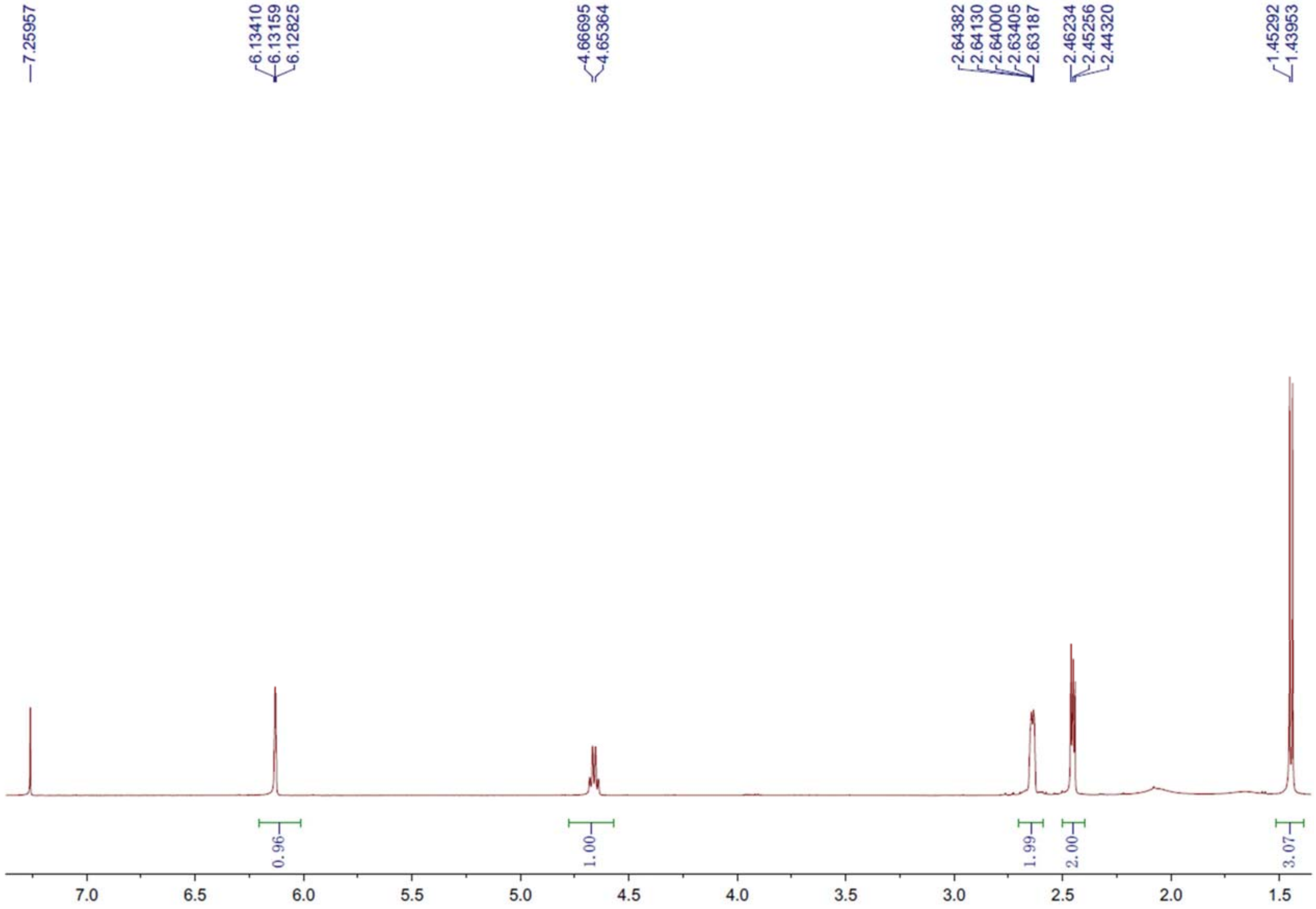

Figure S33. ^{1}H NMR spectrum of compound **5** in $CDCl_3$.

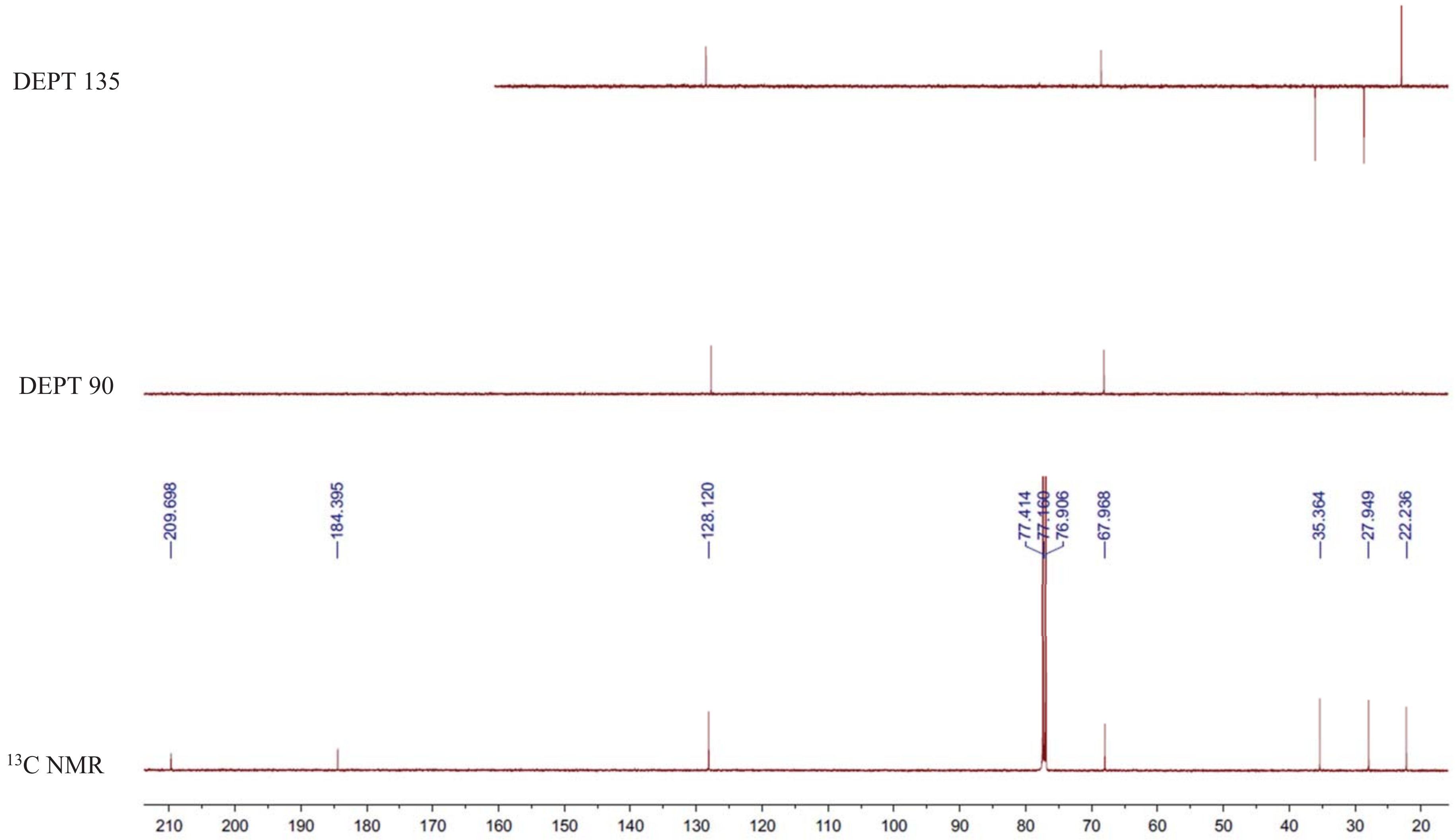

Figure S34. ^{13}C NMR and DEPT spectra of compound **5** in $CDCl_3$.

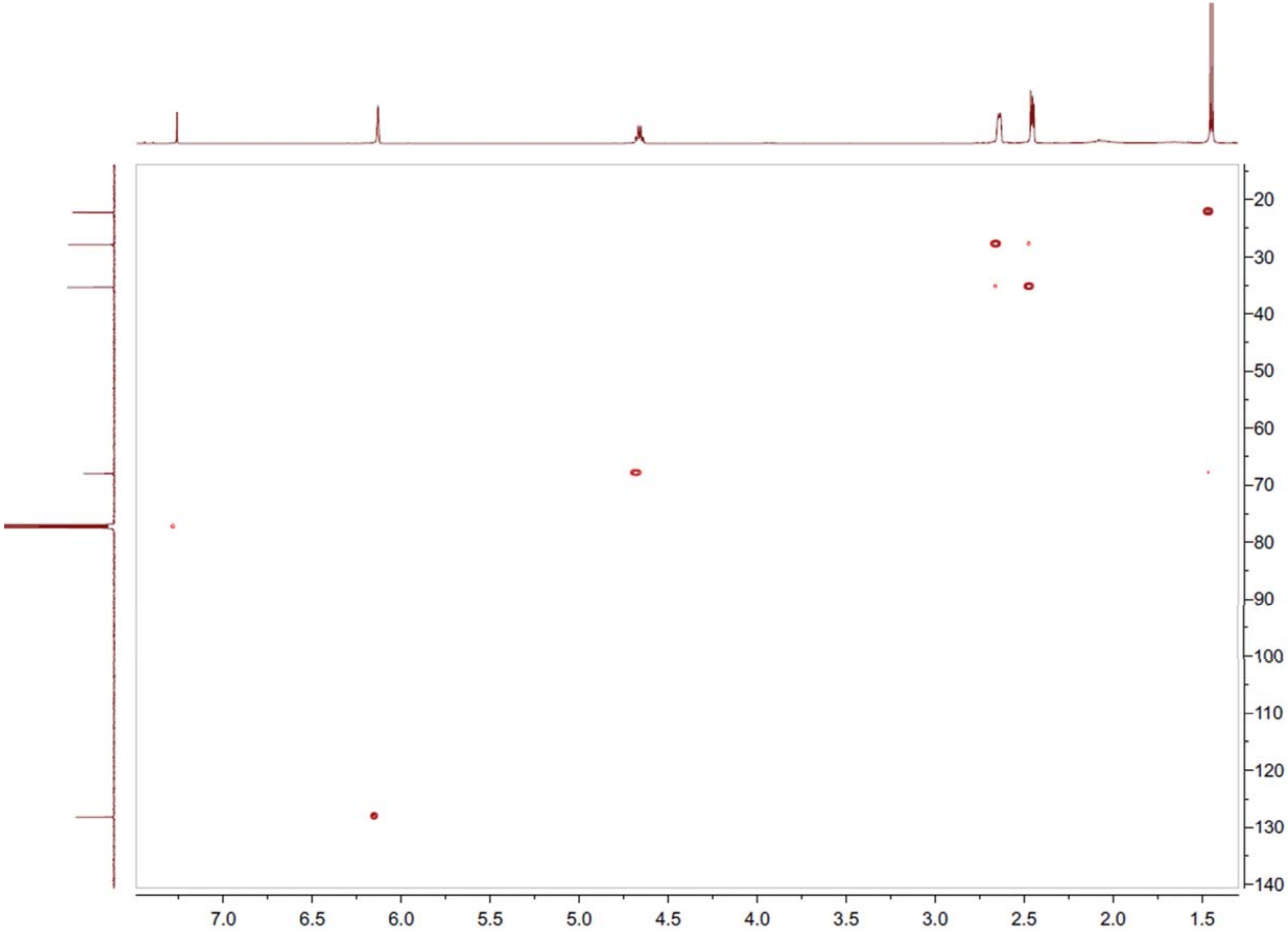

Figure S35. HSQC spectrum of compound **5** in $CDCl_3$.

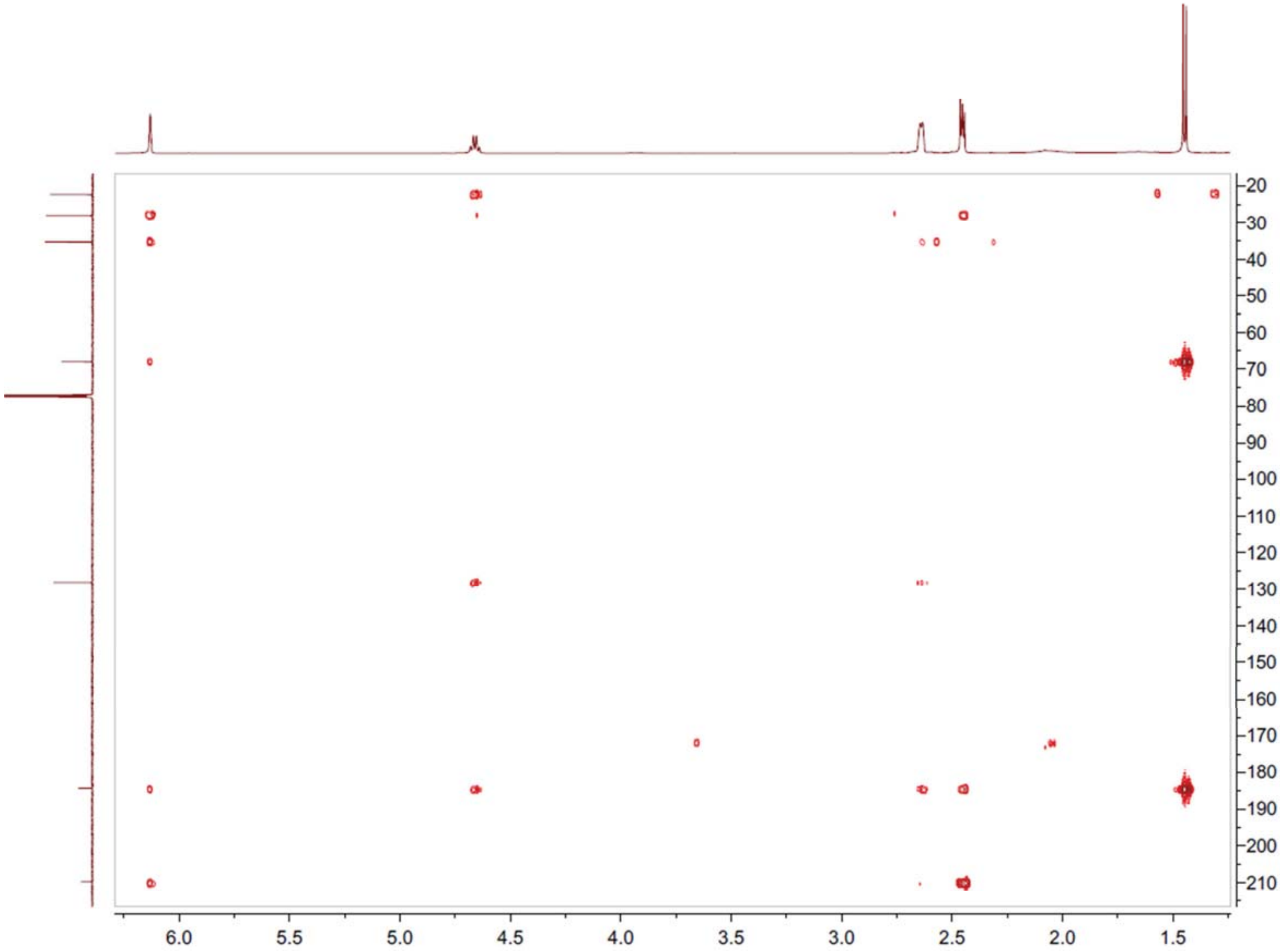

Figure S36. HMBC spectrum of compound **5** in $CDCl_3$.

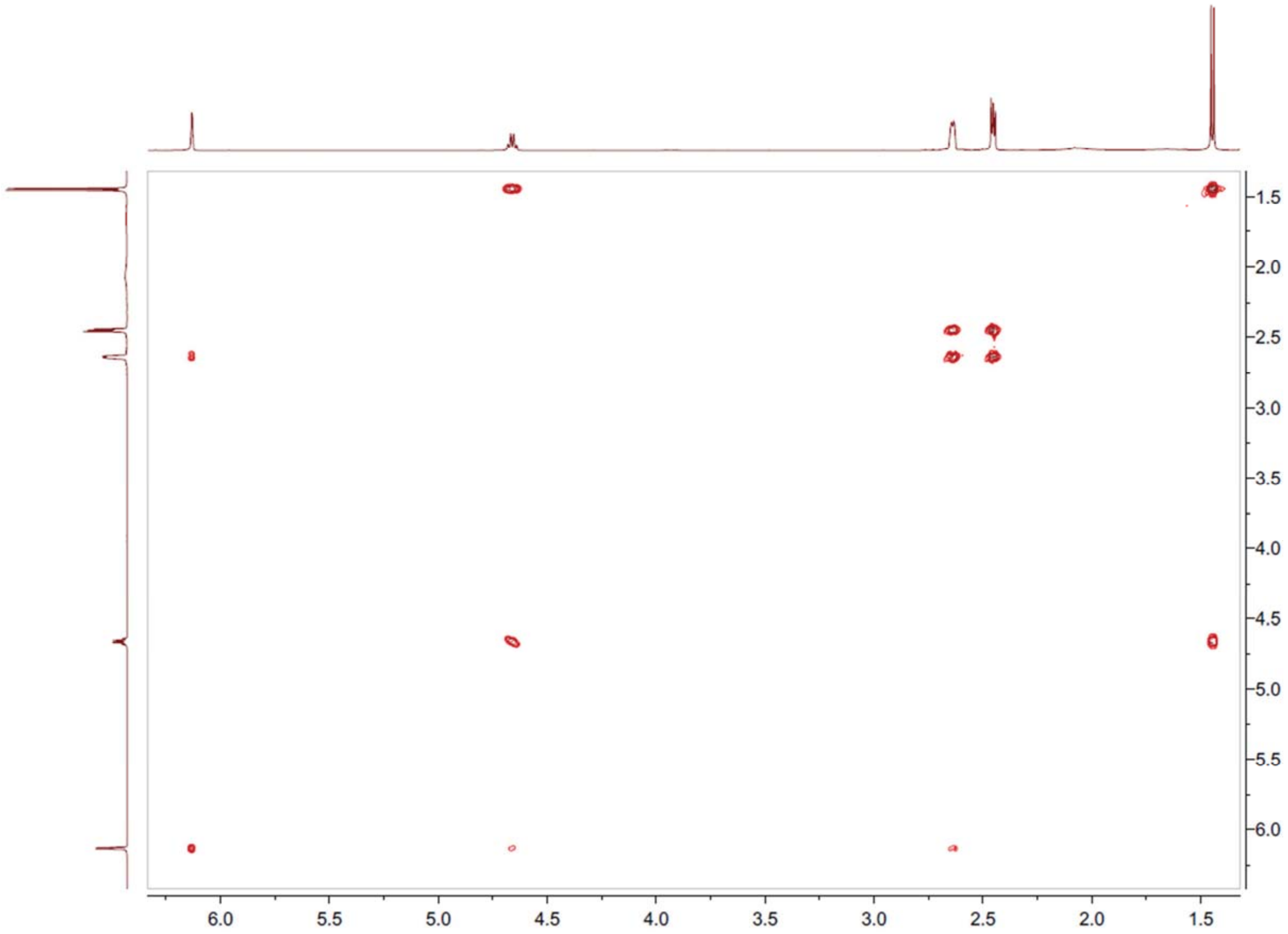

Figure S37. COSY spectrum of compound **5** in $CDCl_3$.

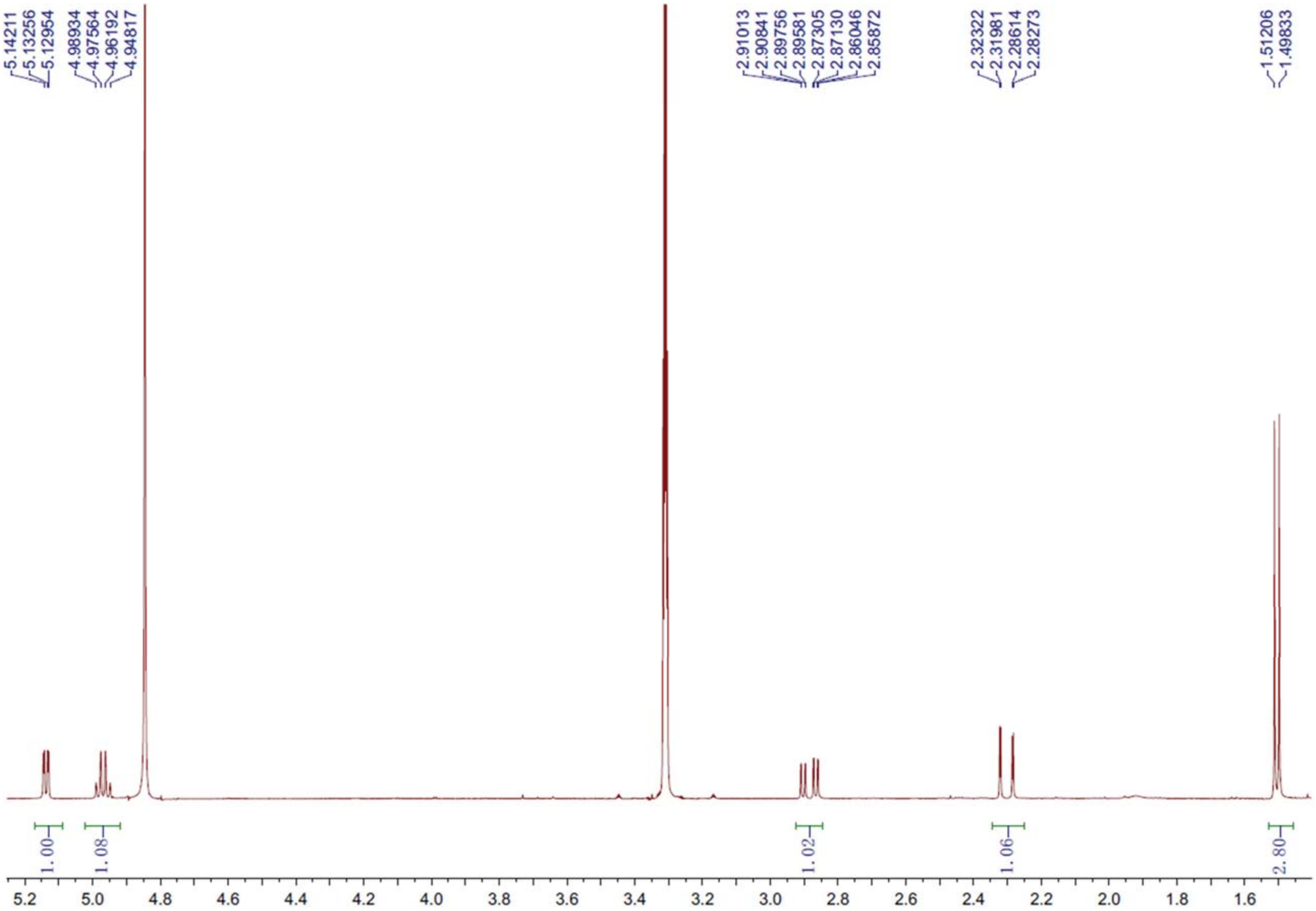

Figure S38. ^{1}H NMR spectrum of compound **6** in CD_3OD.

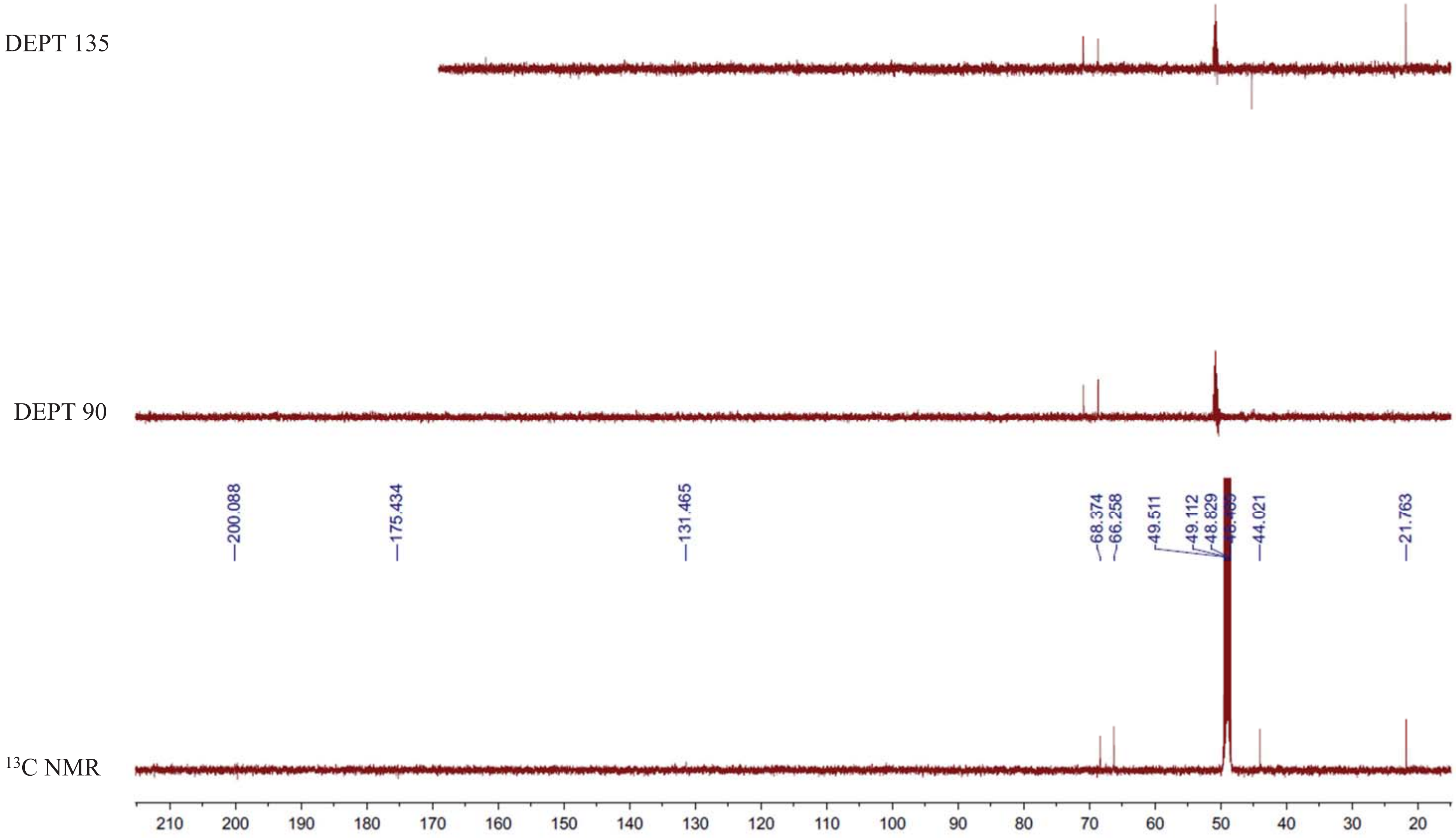

Figure S39. ^{13}C NMR and DEPT spectra of compound **6** in CD_3OD.

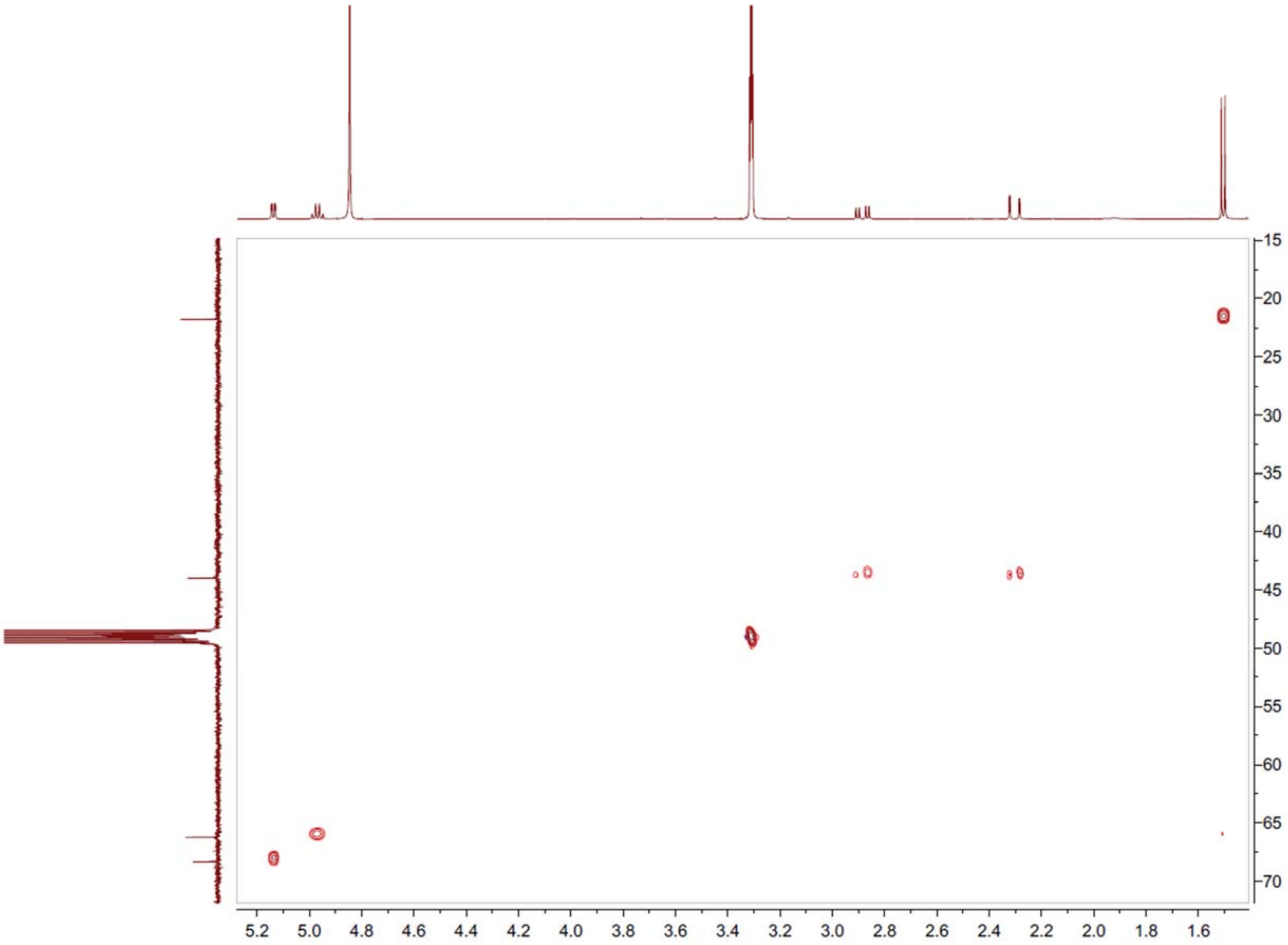

Figure S40. HSQC spectrum of compound **6** in CD_3OD.

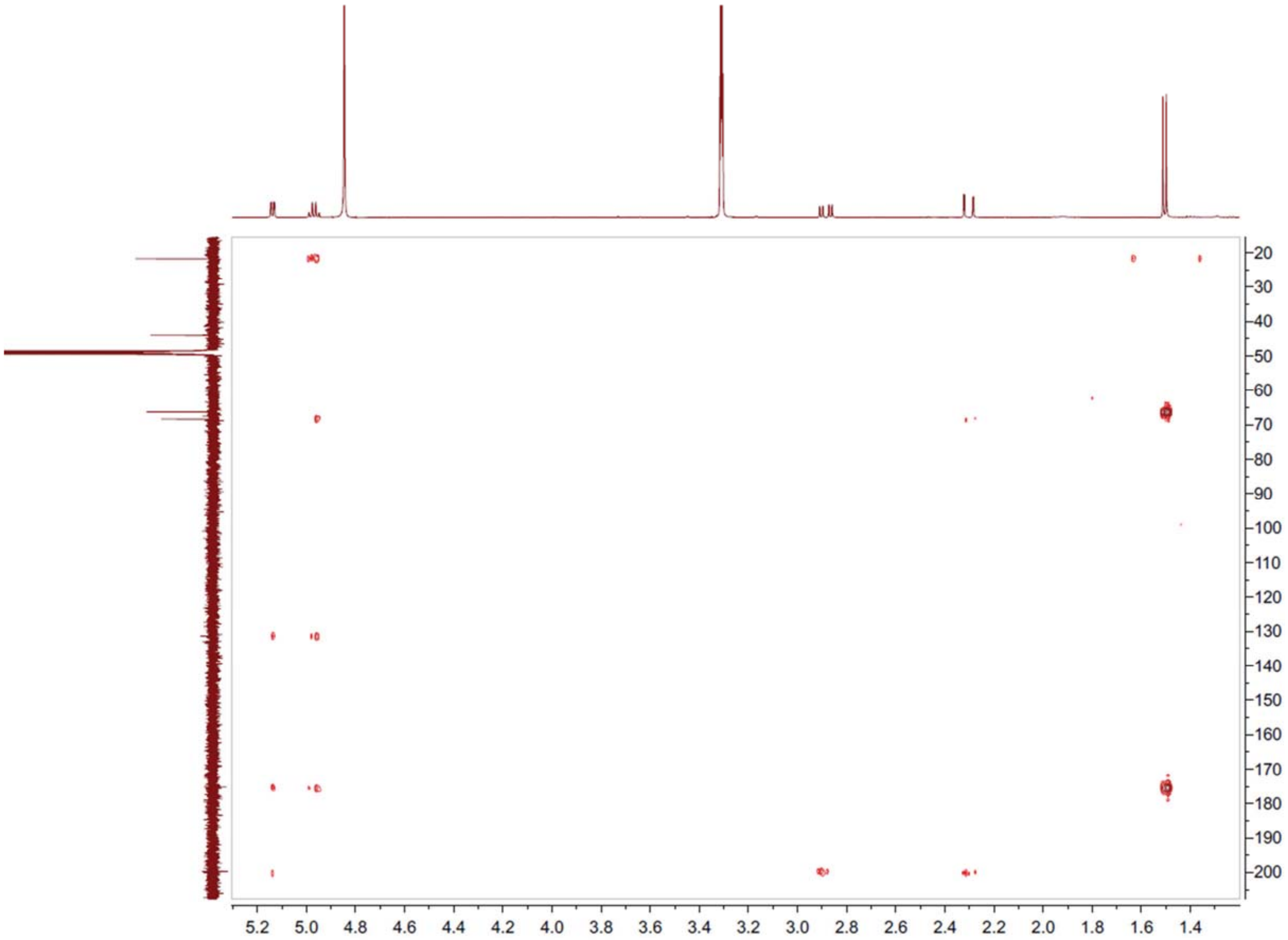

Figure S41. HMBC spectrum of compound **6** in CD_3OD.

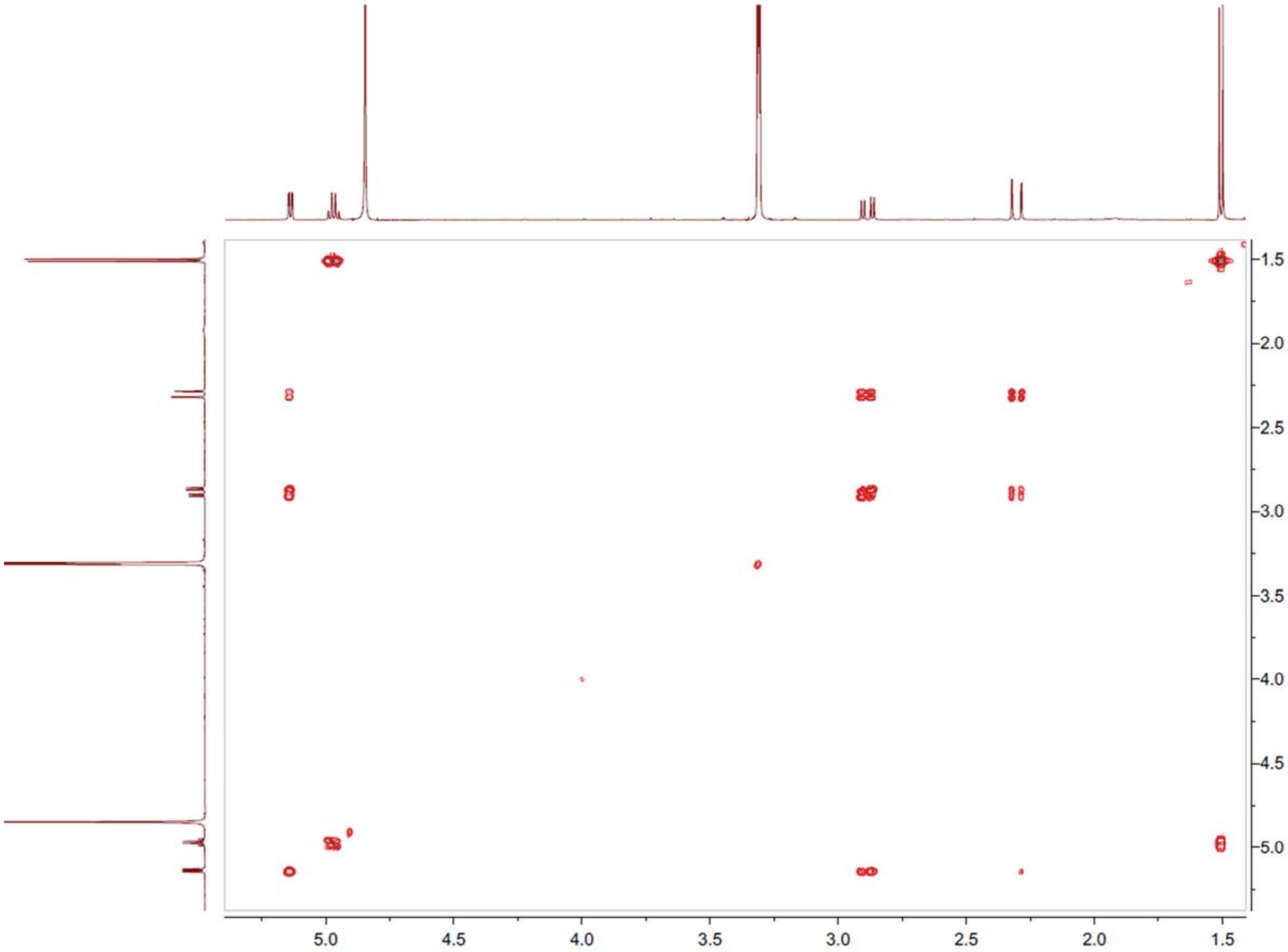

Figure S42. COSY spectrum of compound **6** in CD_3OD.

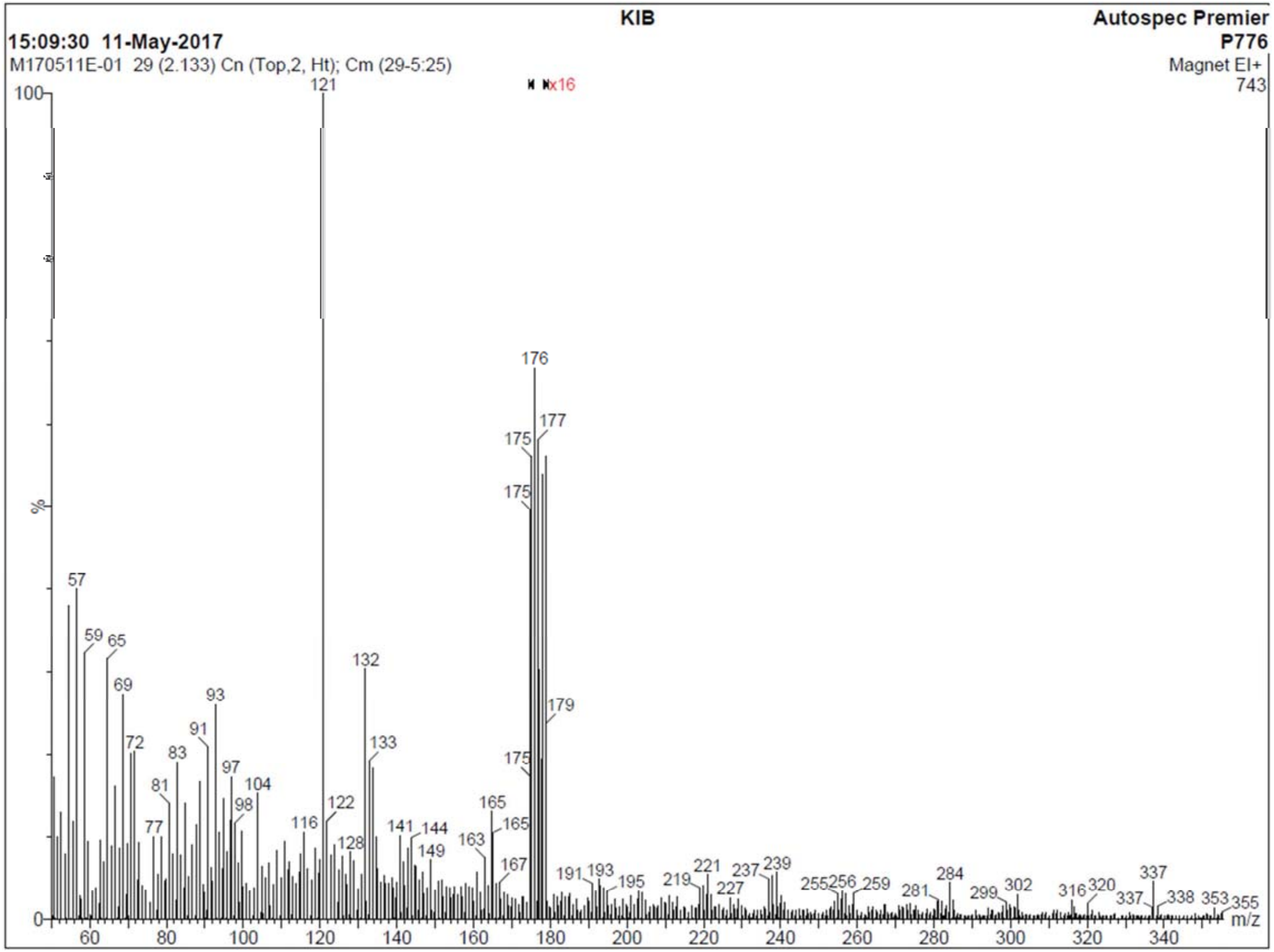

Figure S43. EIMS spectrum of compound **6**.

Single Mass Analysis
Tolerance = 10.0 PPM / DBE: min = -10.0, max = 120.0
Selected filters: None

Monoisotopic Mass, Odd and Even Electron Ions
10 formula(e) evaluated with 1 results within limits (up to 51 closest results for each mass)
Elements Used:
C: 0-200 H: 0-400 O: 2-4 Cl: 1-1

KIB
16:48:13 11-May-2017 M170511EA-01AFAMM 31 (2.849) Autospec Premier
Voltage EI+ P776
1

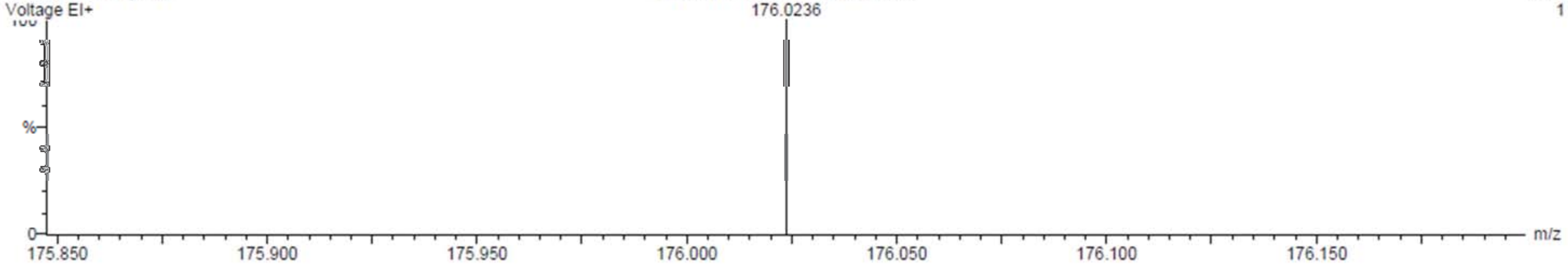

Minimum:				-10.0		
Maximum:		200.0	10.0	120.0		
Mass	Calc. Mass	mDa	PPM	DBE	i-FIT	Formula
176.0236	176.0240	-0.4	-2.3	3.0	5546026.0	C7 H9 O3 Cl

Figure S44. HREIMS spectrum of compound **6**.

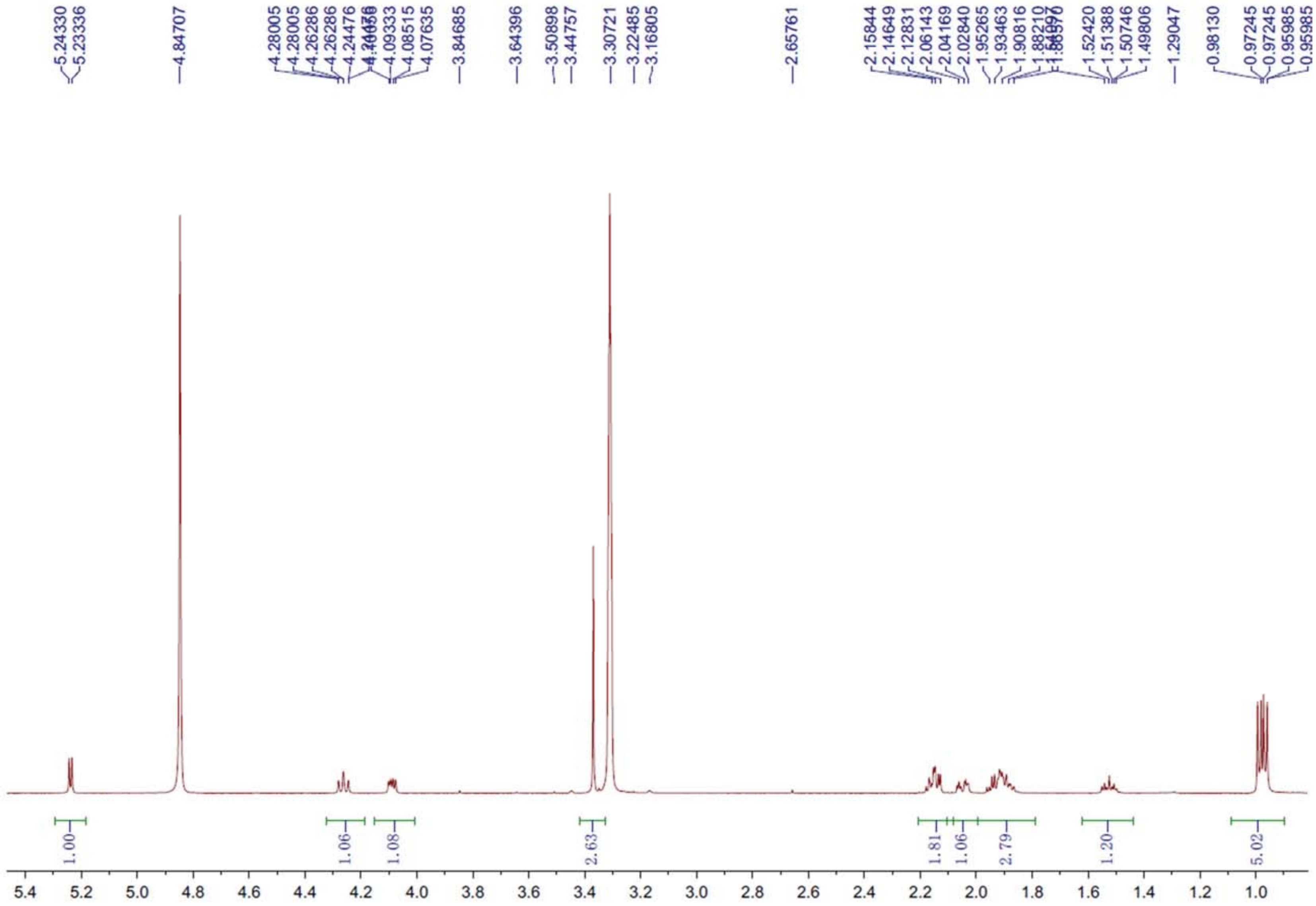

Figure S45. ^{1}H NMR spectrum of compound **7** in CD_3OD.

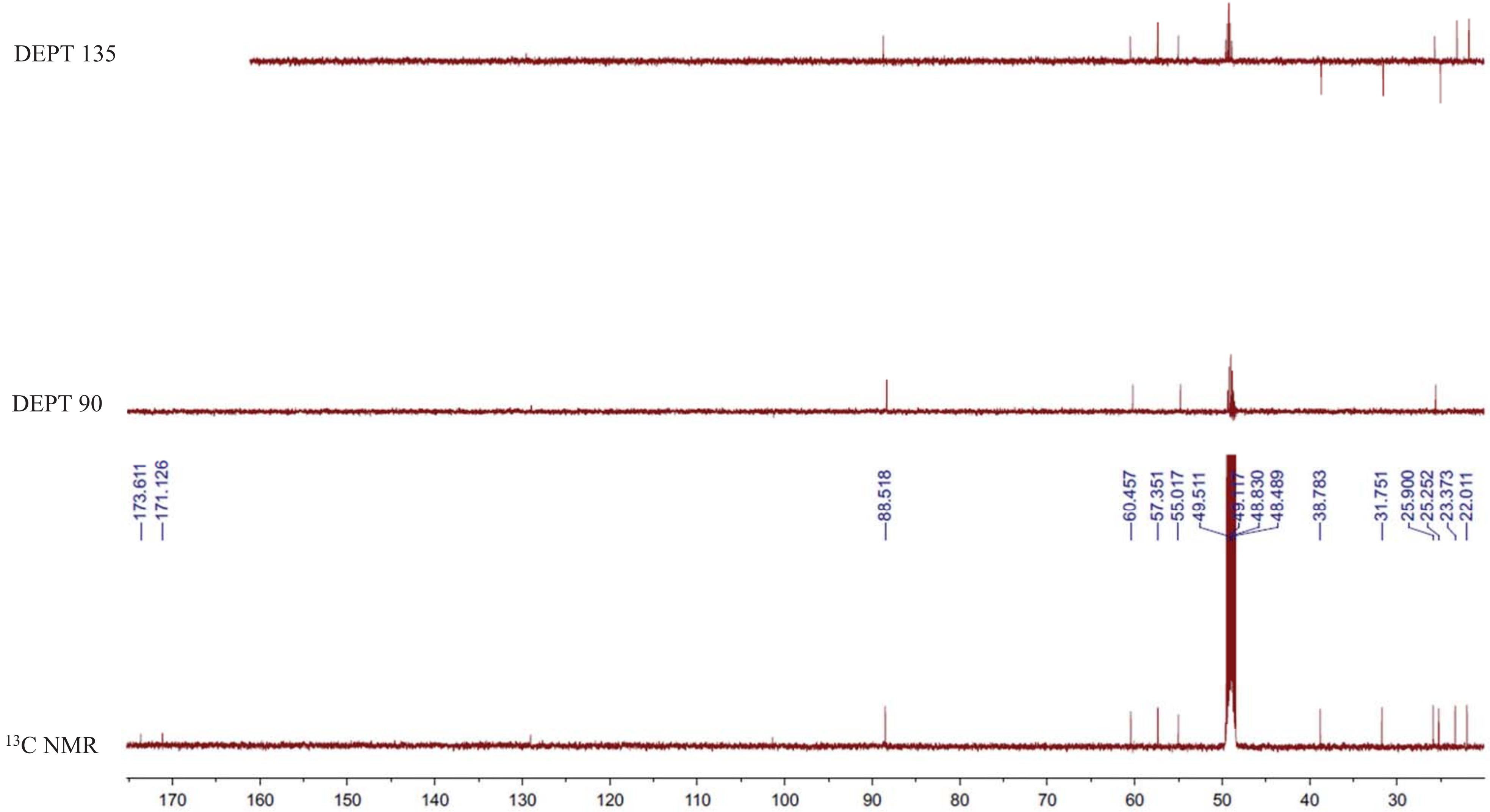

Figure S46. ^{13}C NMR and DEPT spectra of compound **7** in CD_3OD.

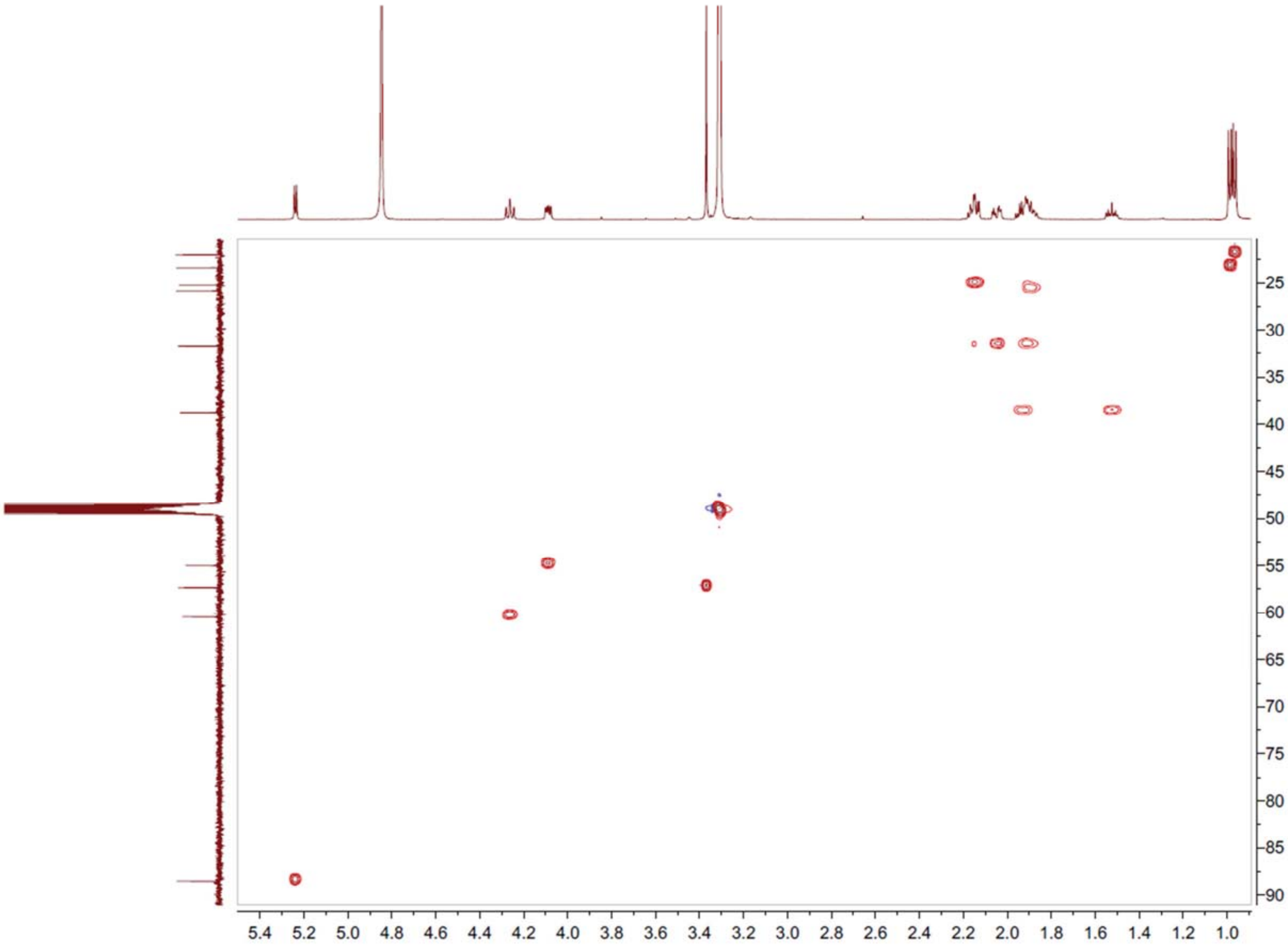

Figure S47. HSQC spectrum of compound **7** in CD_3OD.

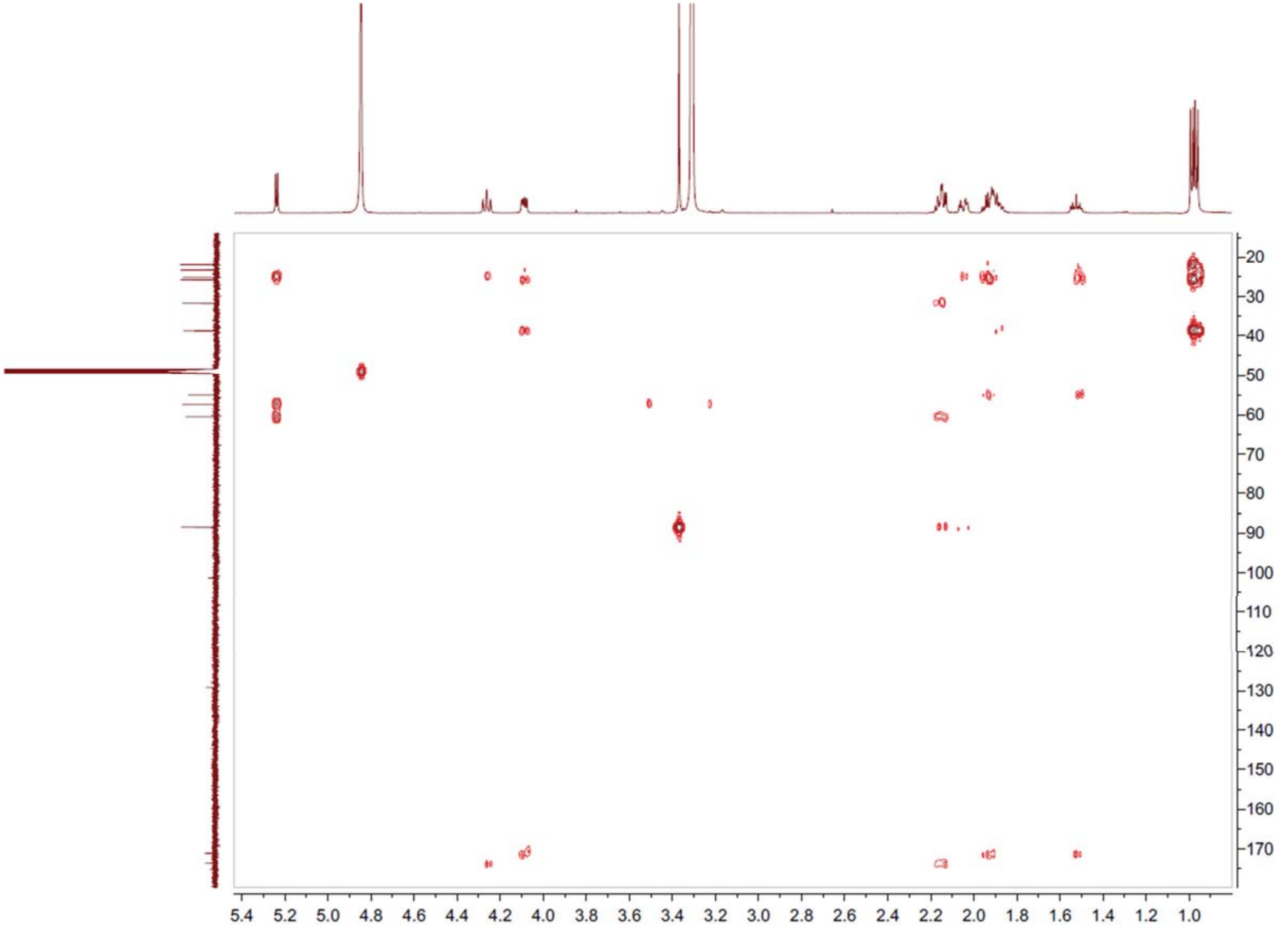

Figure S48. HMBC spectrum of compound **7** in CD_3OD.

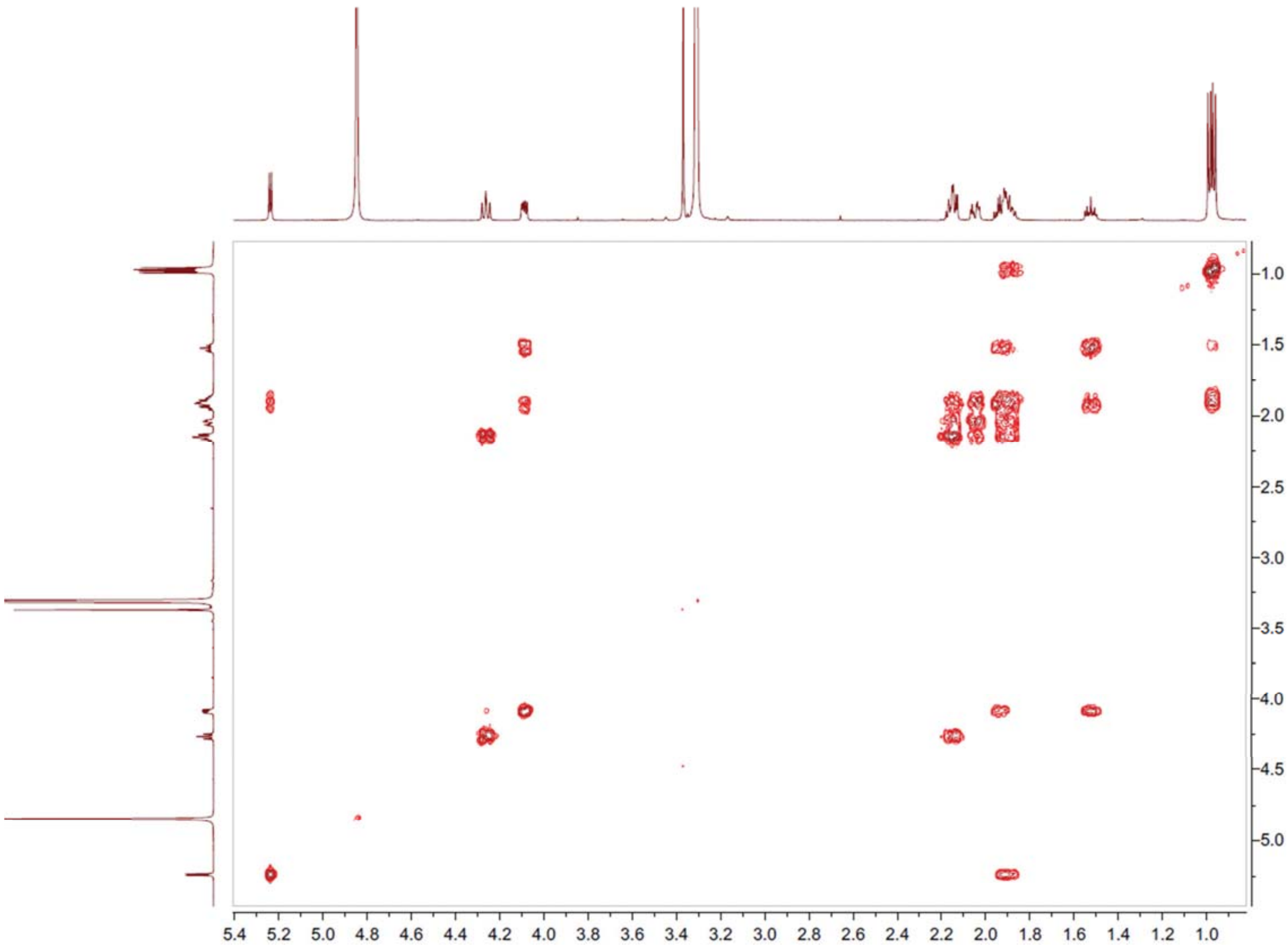

Figure S49. COSY spectrum of compound **7** in CD_3OD.

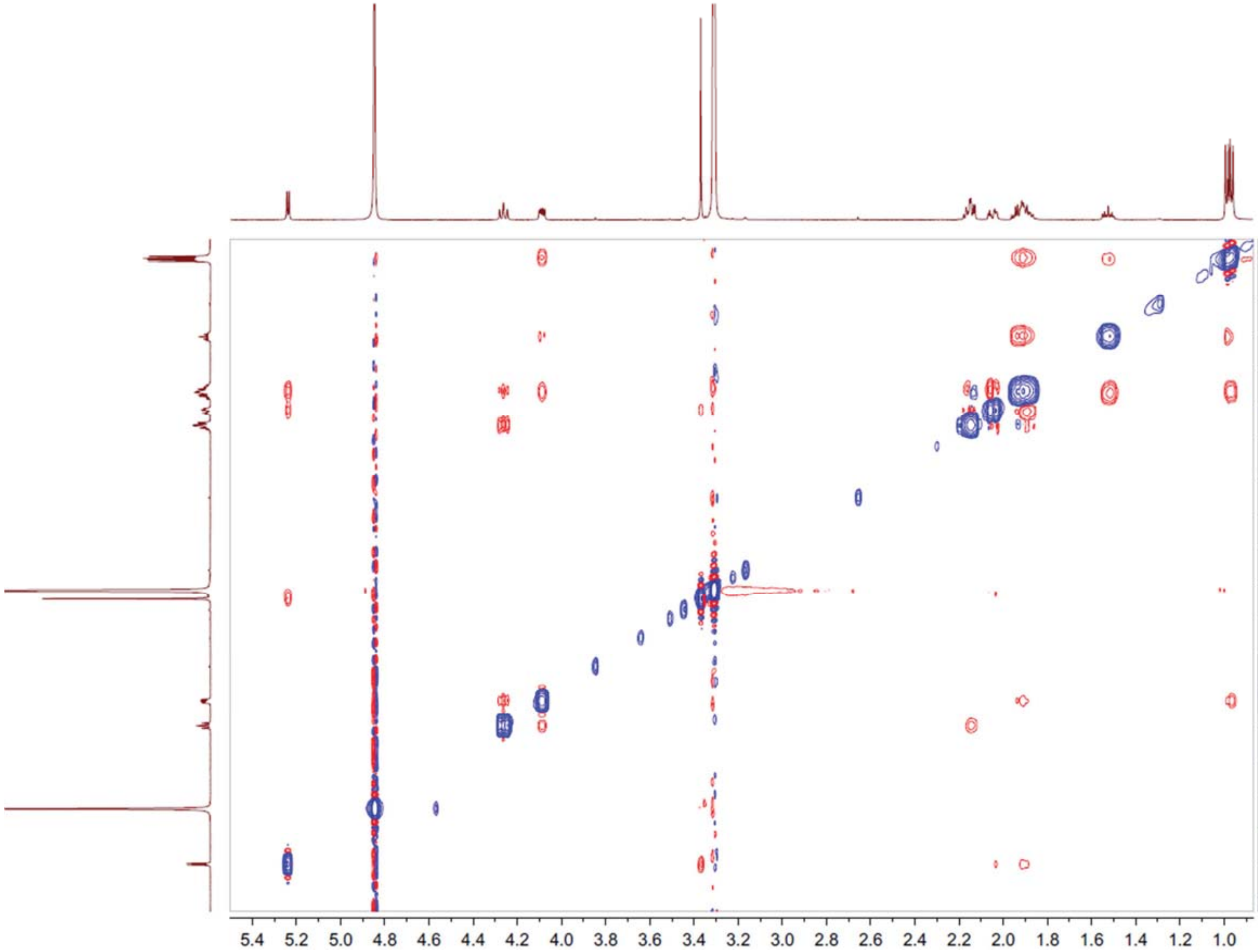

Figure S50. NOESY spectrum of compound **7** in CD_3OD.

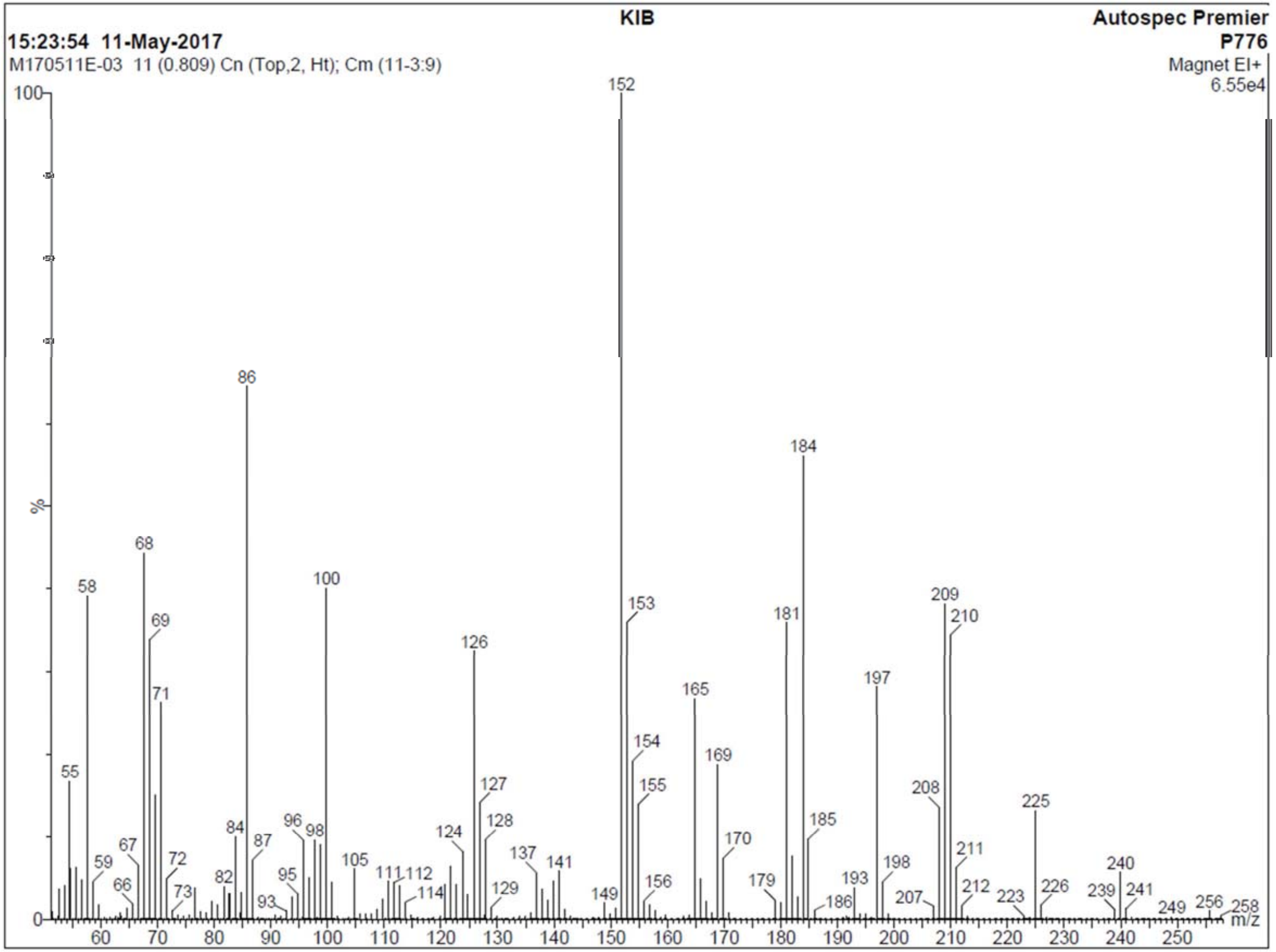

Figure S51. EIMS spectrum of compound **7**.

Single Mass Analysis
Tolerance = 10.0 PPM / DBE: min = -10.0, max = 120.0
Selected filters: None

Monoisotopic Mass, Odd and Even Electron Ions
13 formula(e) evaluated with 1 results within limits (up to 51 closest results for each mass)
Elements Used:
C: 0-200 H: 0-400 N: 2-2 O: 2-4

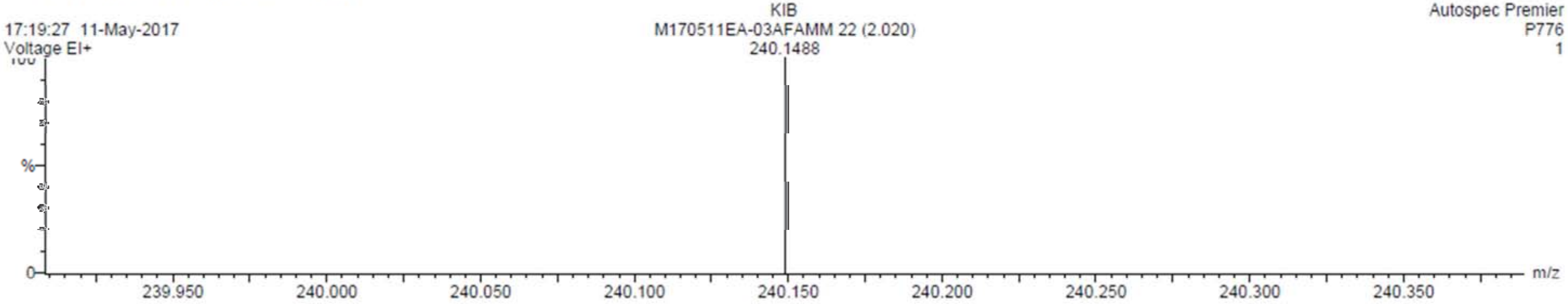

Minimum:				-10.0		
Maximum:		200.0	10.0	120.0		
Mass	Calc. Mass	mDa	PPM	DBE	i-FIT	Formula
240.1488	240.1474	1.4	5.8	4.0	5546026.0	C12 H20 N2 O3

Figure S52. HREIMS spectrum of compound **7**.

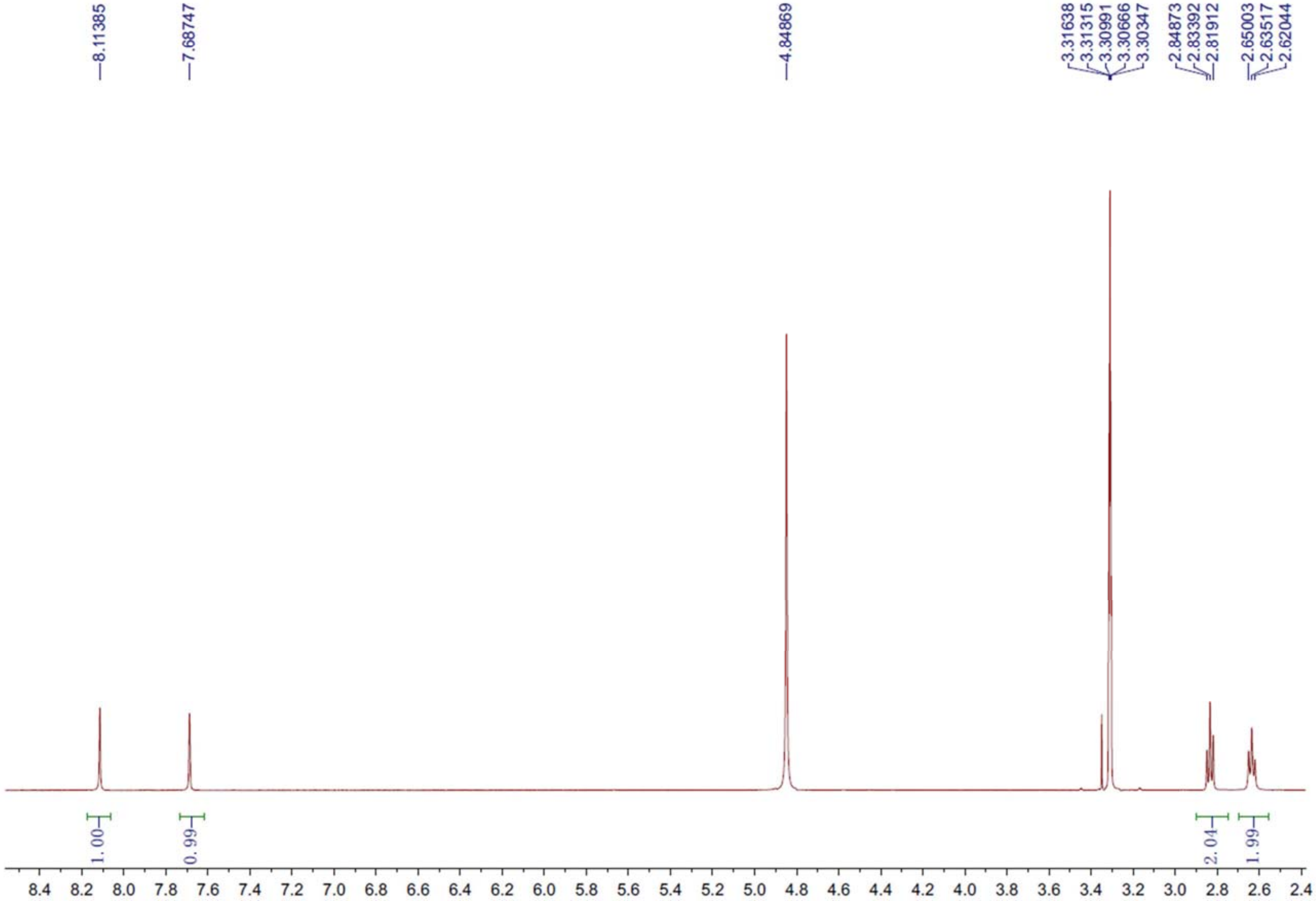

Figure S53. ^{1}H NMR spectrum of compound **8** in CD_3OD.

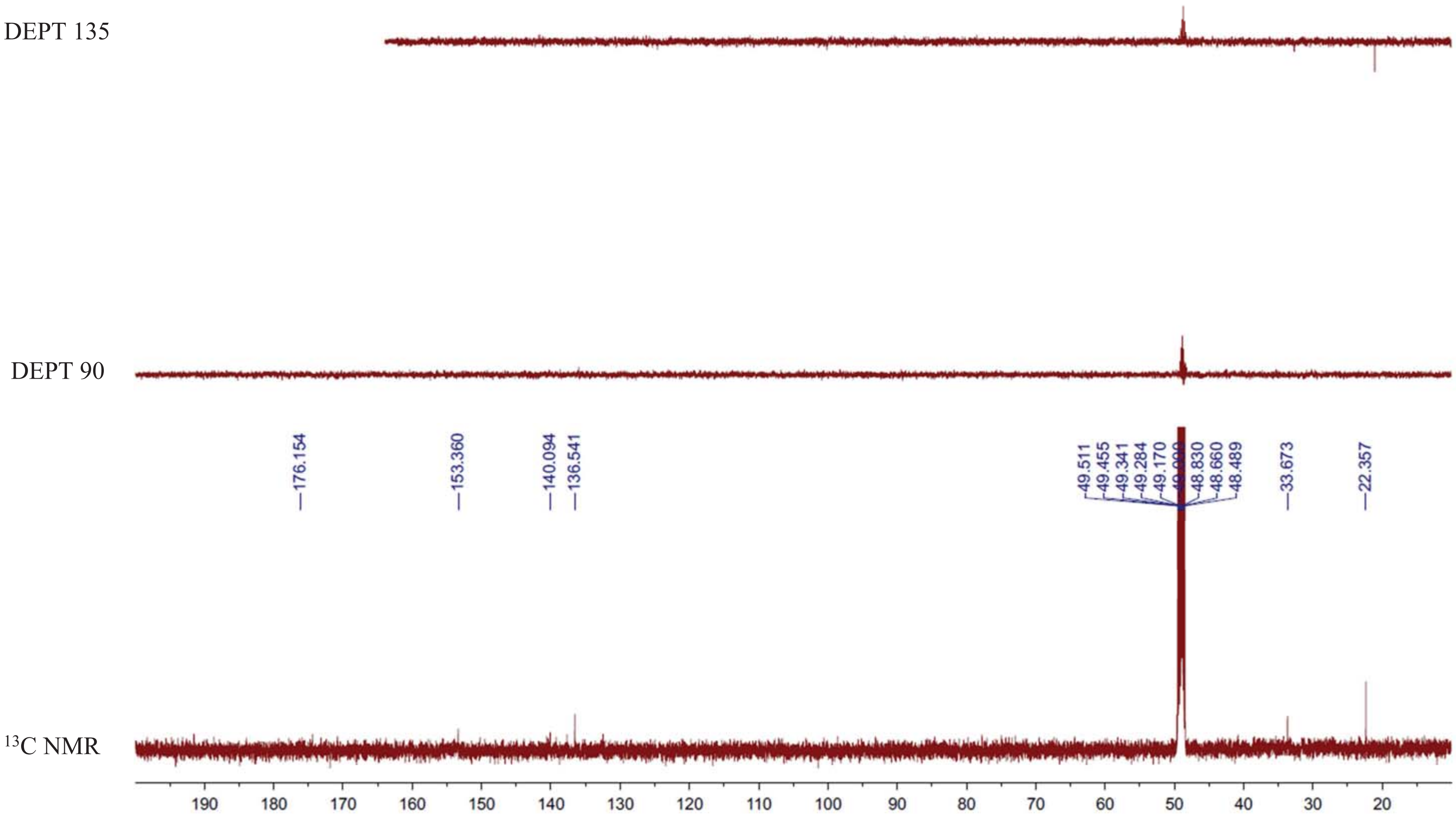

Figure S54. ^{13}C NMR and DEPT spectra of compound **8** in CD_3OD.

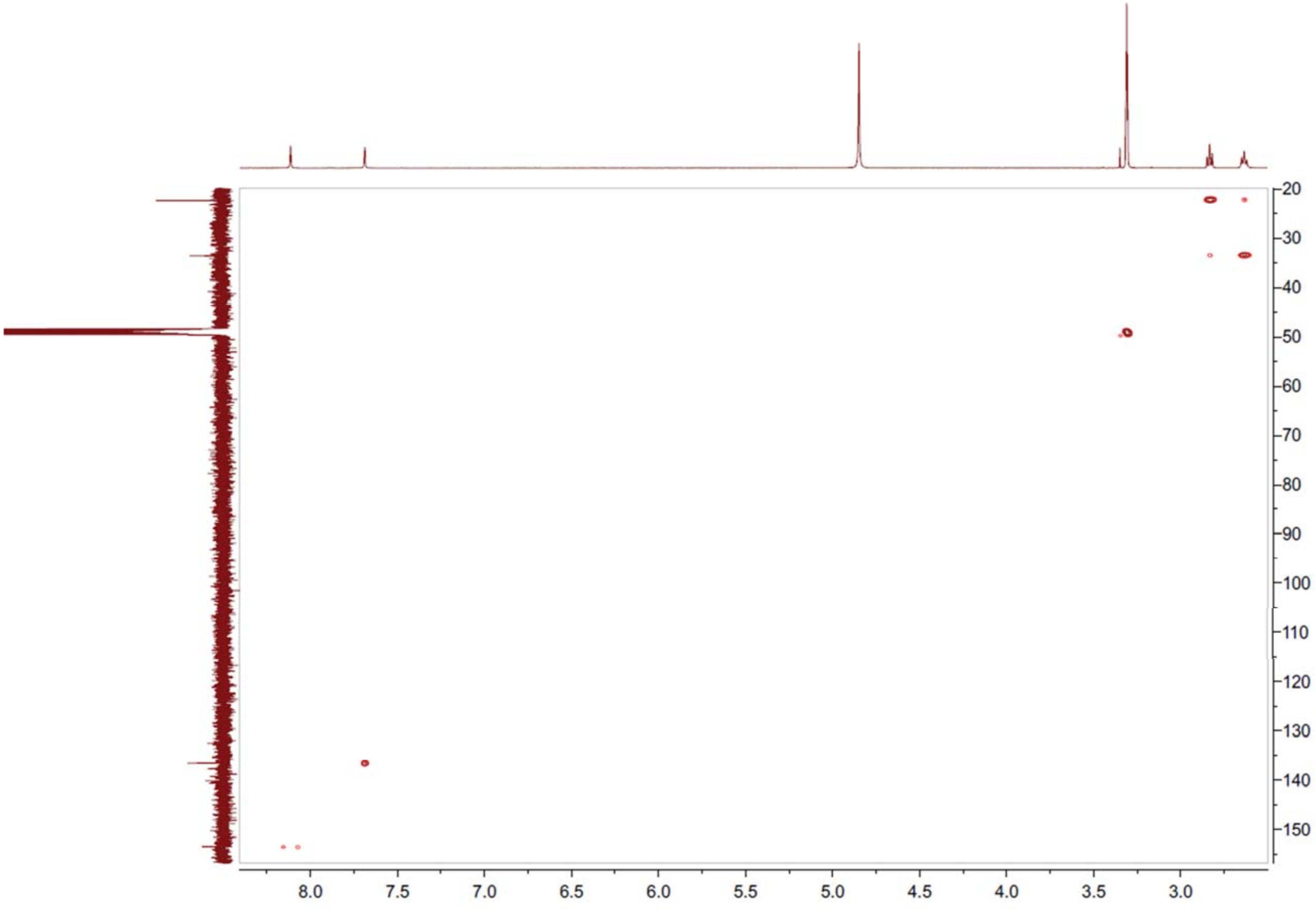

Figure S55. HSQC spectrum of compound **8** in CD_3OD.

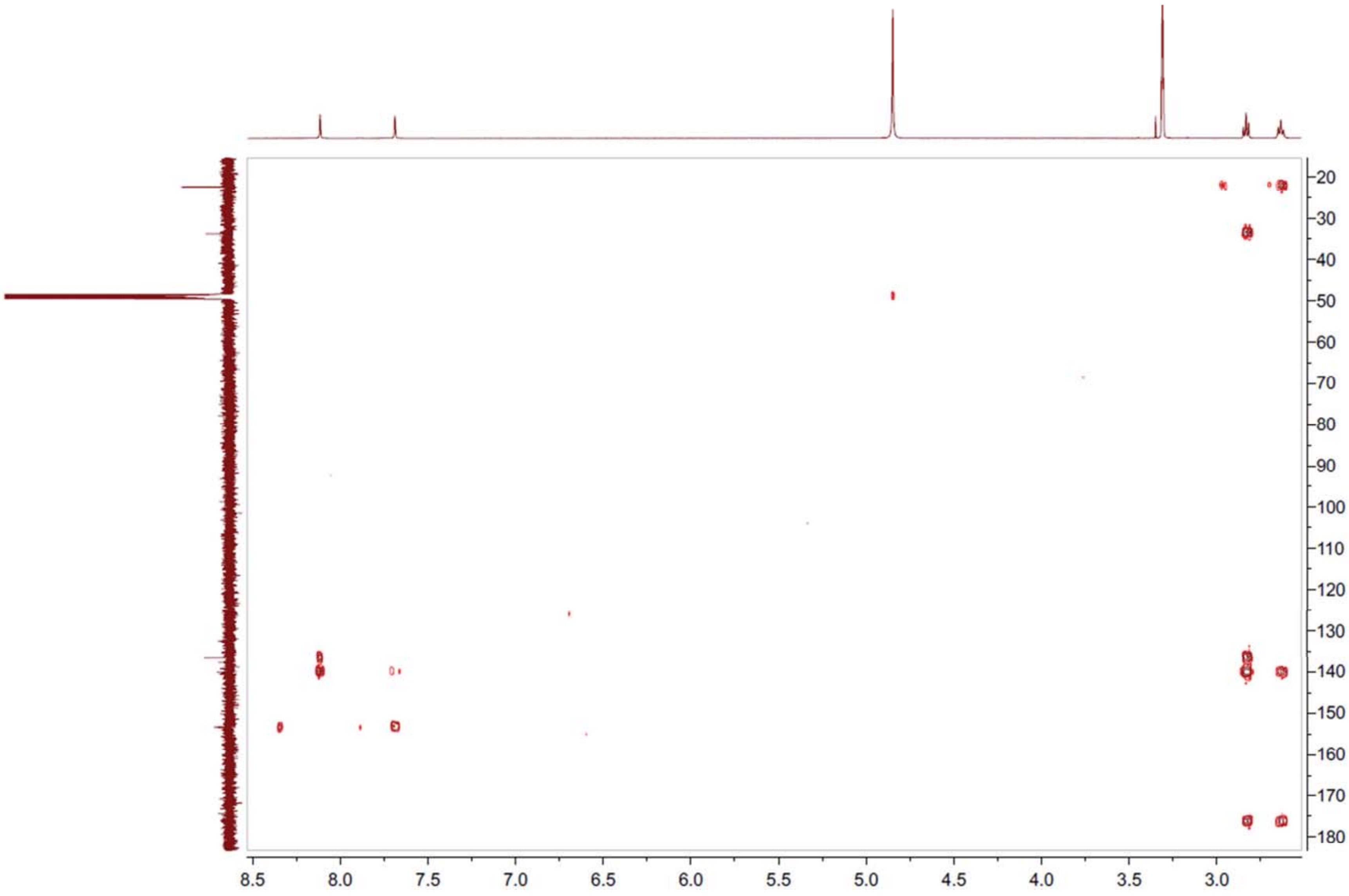

Figure S56. HMBC spectrum of compound **8** in CD_3OD.

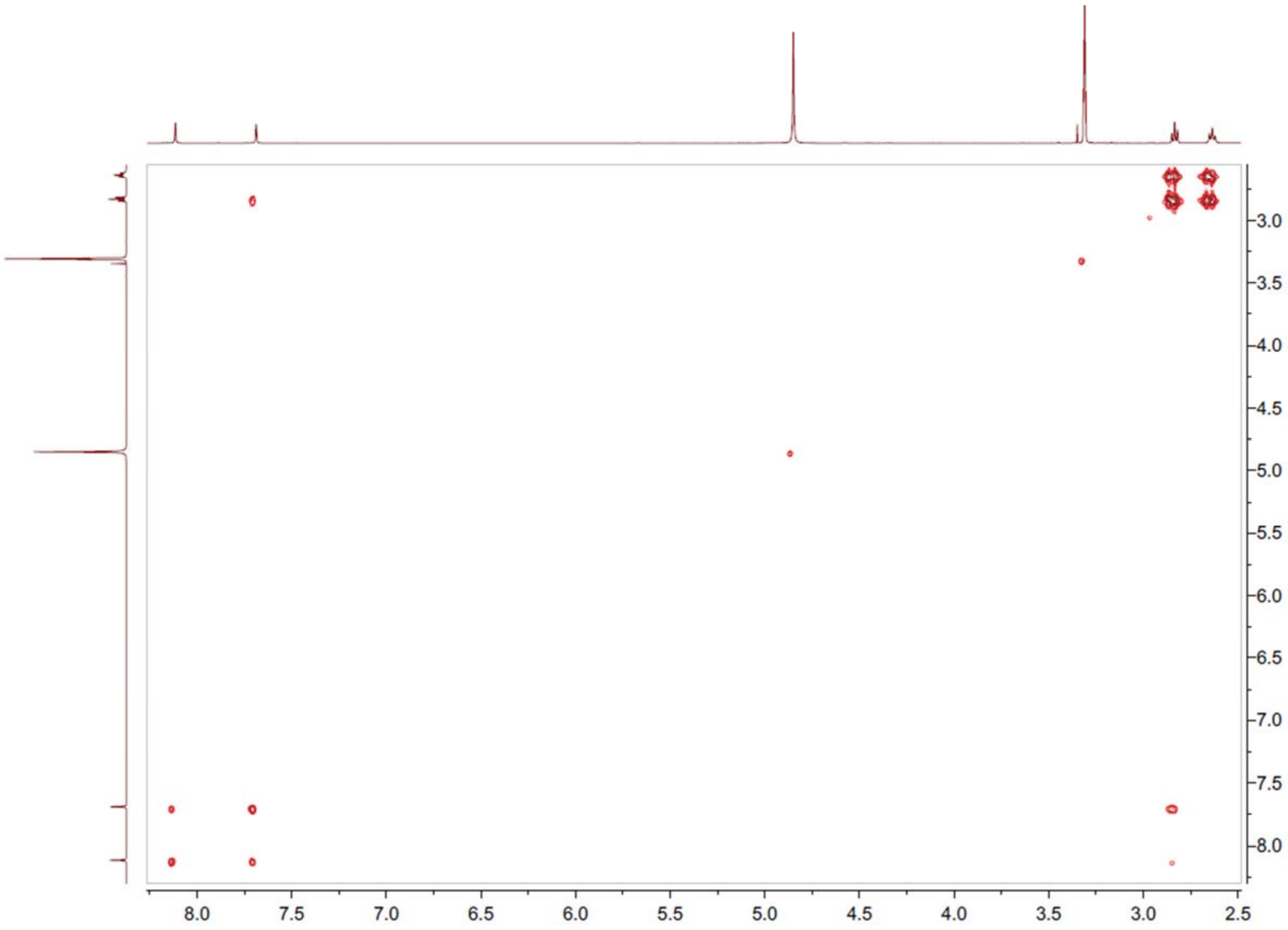

Figure S57. COSY spectrum of compound **8** in CD_3OD.

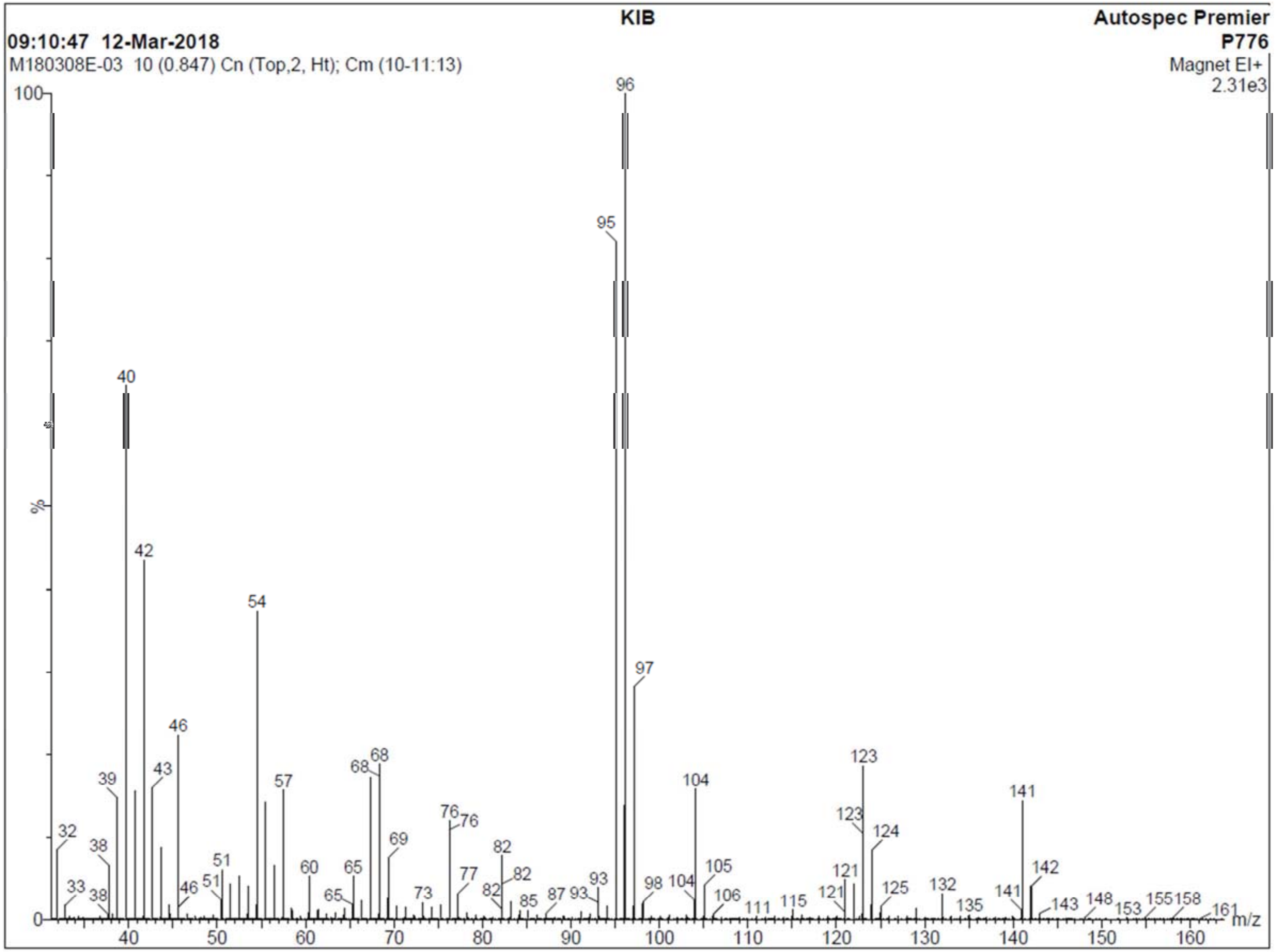

Figure S58. EIMS spectrum of compound **8**.

Single Mass Analysis
Tolerance = 10.0 PPM / DBE: min = -10.0, max = 120.0
Selected filters: None

Monoisotopic Mass, Odd and Even Electron Ions
10 formula(e) evaluated with 1 results within limits (up to 51 closest results for each mass)
Elements Used:
C: 0-200 H: 0-400 N: 1-1 O: 2-4

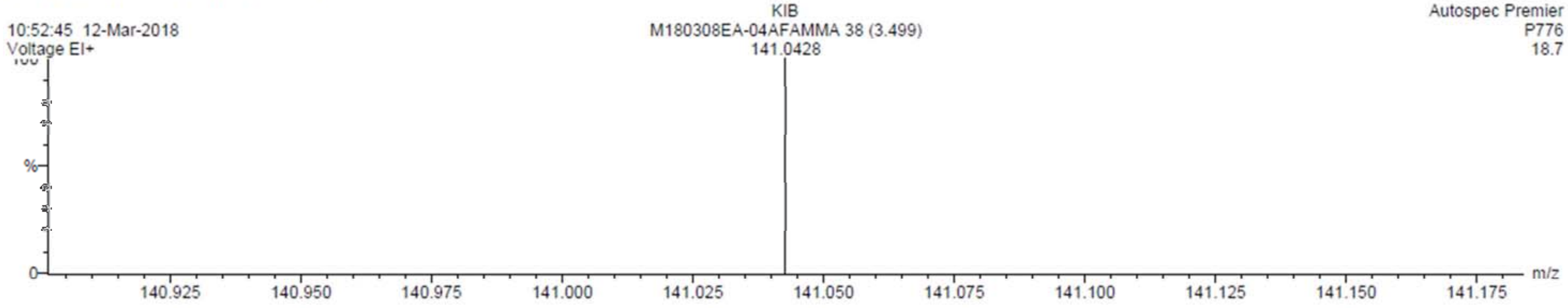

Minimum:				-10.0		
Maximum:		200.0	10.0	120.0		
Mass	Calc. Mass	mDa	PPM	DBE	i-FIT	Formula
141.0428	141.0426	0.2	1.4	4.0	5546025.5	C6 H7 N O3

Figure S59. HREIMS spectrum of compound **8**.

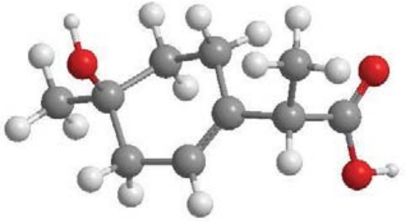

a (67.4%)

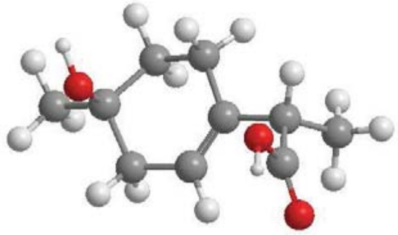

b (19.3%)

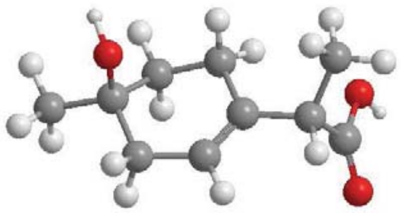

c (9.8%)

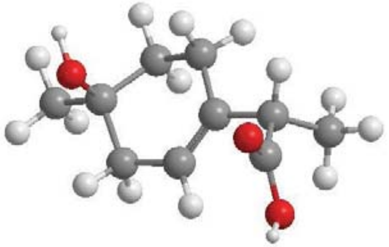

d (3.5%)

Figure S60. Energy-minimized conformers with populations of (3*S*,7*S*)-1-hydroxy-3-*p*-menthen-9-oic acid. (Regardless of rotations of methyl and hydroxy groups)

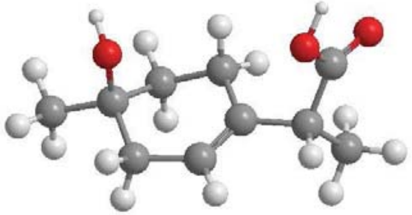

a (56.0%)

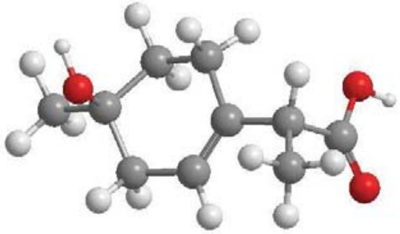

b (27.0%)

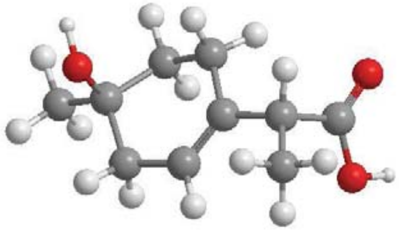

c (9.6%)

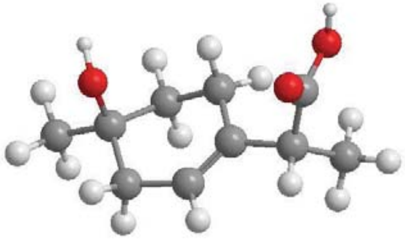

d (7.4%)

Figure S61. Energy-minimized conformers with populations of (3*S*,7*R*)-1-hydroxy-3-*p*-menthen-9-oic acid. (Regardless of rotations of methyl and hydroxy groups)

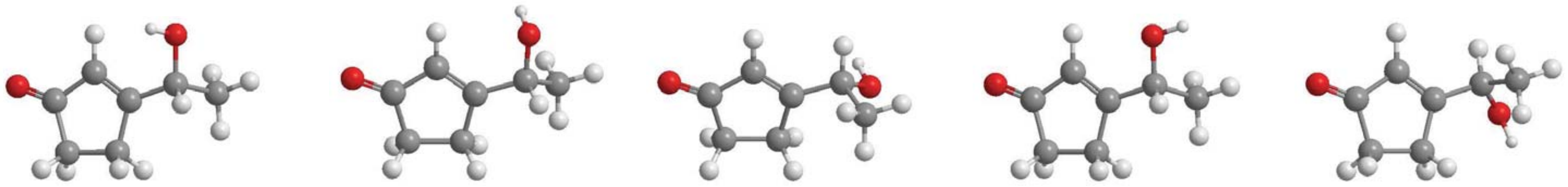

a (29.7) **b** (15.0%) **c** (14.1%) **d** (13.7%) **e** (6.2%)

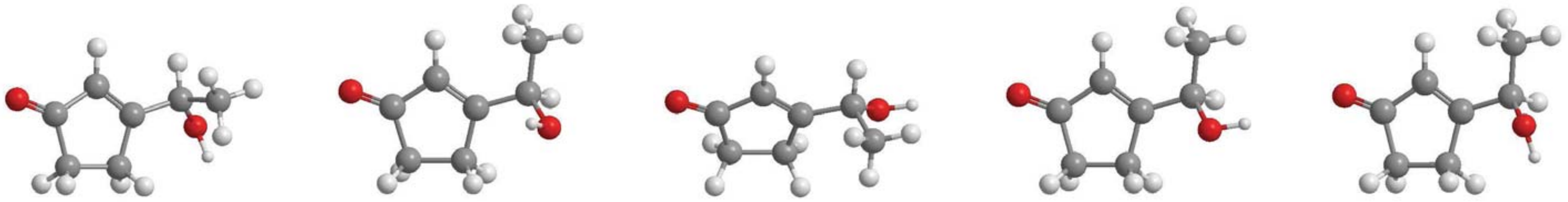

f (6.0%) **g** (5.5%) **h** (5.0%) **i** (2.9%) **j** (1.8%)

Figure S62. Energy-minimized conformers with populations of compound **5**.

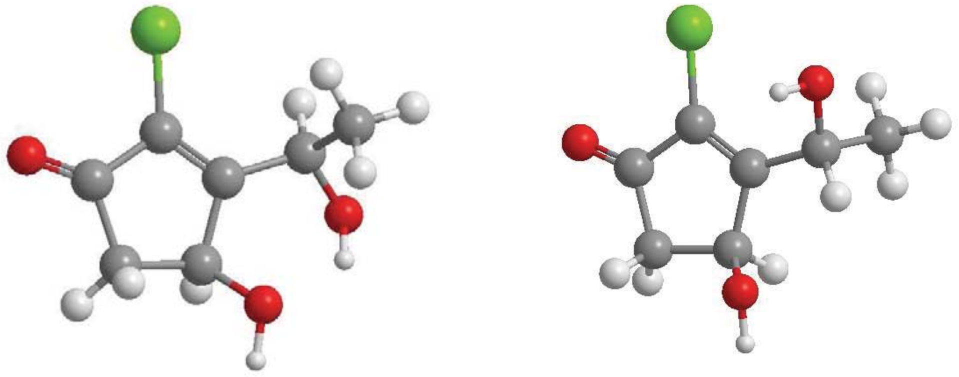

a (98.6%) **b** (1.4%)

Figure S63. Energy-minimized conformers with populations of (3*S*,6*R*)-3-hydroxytrichodenone C. (Regardless of rotations of methyl and hydroxy groups)

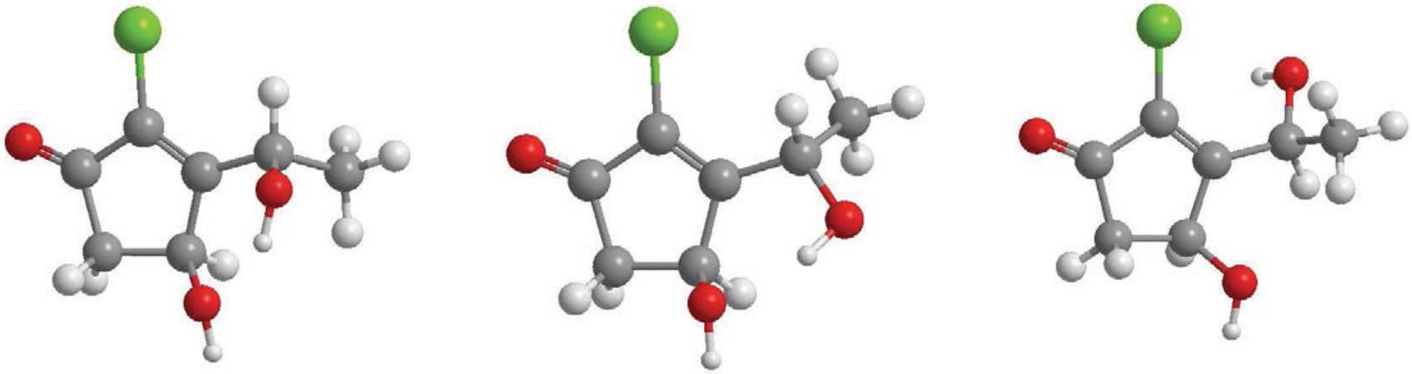

a (59.8%) **b** (39.0%) **c** (1.2%)

Figure S64. Energy-minimized conformers with populations of (3*S*,6*S*)-3-hydroxytrichodenone C. (Regardless of rotations of methyl and hydroxy groups)
